# Predictive evolution of metabolic phenotypes using model‐designed environments

**DOI:** 10.15252/msb.202210980

**Published:** 2022-10-06

**Authors:** Paula Jouhten, Dimitrios Konstantinidis, Filipa Pereira, Sergej Andrejev, Kristina Grkovska, Sandra Castillo, Payam Ghiachi, Gemma Beltran, Eivind Almaas, Albert Mas, Jonas Warringer, Ramon Gonzalez, Pilar Morales, Kiran R Patil

**Affiliations:** ^1^ European Molecular Biology Laboratory Heidelberg Germany; ^2^ VTT Technical Research Centre of Finland Ltd Espoo Finland; ^3^ Department of Bioproducts and Biosystems Aalto University Espoo Finland; ^4^ Department of Chemistry and Molecular Biology University of Gothenburg Gothenburg Sweden; ^5^ Departament Bioquímica i Biotecnologia, Facultat d'Enologia Universitat Rovira i Virgili Tarragona Spain; ^6^ Department of Biotechnology and Food Science NTNU – Norwegian University of Science and Technology Trondheim Norway; ^7^ Instituto de Ciencias de la Vid y delVino (CSIC, Gobierno de la Rioja, Universidad de La Rioja) Finca La Grajera Logroño Spain; ^8^ Medical Research Council (MRC) Toxicology Unit University of Cambridge Cambridge UK

**Keywords:** adaptive evolution, genome‐scale metabolic model, predictive evolution, *Saccharomyces cerevisiae*, wine aroma, Biotechnology & Synthetic Biology, Metabolism, Methods & Resources

## Abstract

Adaptive evolution under controlled laboratory conditions has been highly effective in selecting organisms with beneficial phenotypes such as stress tolerance. The evolution route is particularly attractive when the organisms are either difficult to engineer or the genetic basis of the phenotype is complex. However, many desired traits, like metabolite secretion, have been inaccessible to adaptive selection due to their trade‐off with cell growth. Here, we utilize genome‐scale metabolic models to design nutrient environments for selecting lineages with enhanced metabolite secretion. To overcome the growth‐secretion trade‐off, we identify environments wherein growth becomes correlated with a secondary trait termed tacking trait. The latter is selected to be coupled with the desired trait in the application environment where the trait manifestation is required. Thus, adaptive evolution in the model‐designed selection environment and subsequent return to the application environment is predicted to enhance the desired trait. We experimentally validate this strategy by evolving *Saccharomyces cerevisiae* for increased secretion of aroma compounds, and confirm the predicted flux‐rerouting using genomic, transcriptomic, and proteomic analyses. Overall, model‐designed selection environments open new opportunities for predictive evolution.

## Graphical abstract

EvolveX, a new algorithm enabling model‐guided design of chemical environments for targeted adaptive evolution, is applied to evolve a wine yeast strain for increased aroma secretion.

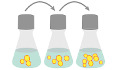

## Introduction

Adaptive evolution under environmental selection pressure can give rise to and optimize complex phenotypes (Darwin, 1859; Shoval *et al*, 2012; Locey & Lennon, 2016; Sunagawa *et al*, 2020). While this evolutionary process can involve numerous alternative paths at the level of genotype, phenotype evolution is often convergent (Barrick *et al*, 2009; Lassig *et al*, 2017). Adaptive evolution under controlled laboratory conditions can thus be used to obtain target phenotypes without explicit knowledge of the causative genotype. This method, known as adaptive laboratory evolution, is widely used for improving microbial strains; examples include temperature tolerance (Sandberg *et al*, 2014; Caspeta & Nielsen, 2015), simplified nutritional requirement (Bracher *et al*, 2017), and boosting photosynthetic capabilities (Antonovsky *et al*, 2016; Gassler *et al*, 2020).

While effective in optimizing complex traits and operationally simple, adaptive laboratory evolution is inherently limited to traits that are genetically linked to the fitness. Consequently, improving fitness‐neutral or costly trait requires artificial, non‐Darwinian, selection through screening of large numbers of variants. This is a considerable combinatorial challenge for complex multigenic traits, and, thus, application of artificial selection has yet been limited to single proteins or pathways with photometric readouts (Arnold, 1993; Wang *et al*, 2009; Lee *et al*, 2013; Chen *et al*, 2018; van Tatenhove‐Pel *et al*, 2020). Darwinian selection of a complex trait requires identification of an environmental condition where the trait becomes genetically growth‐linked (Agrawal & Stinchcombe, 2009), for example, increased antioxidant production could be selected under oxidative damage conditions (Reyes & Kao, 2018). However, such qualitative and sparsely known associations cannot be generalized, calling for predictive models of trait dependences.

Here, we ask whether first‐principle models could enable predicting environments under which a desired trait could be adaptively selected. We base our strategy on genome‐scale metabolic models, which allow predicting metabolic fluxes consistent both with the mass balance constraints and the fitness objectives of the cells (e.g., optimal growth; O'Brien *et al*, 2015; Varma & Palsson, 1994). In the context of laboratory evolution, genome‐scale metabolic models have well predicted fitness improvement and the associated metabolic flux changes (Ibarra *et al*, 2002; Szappanos *et al*, 2016; Strucko *et al*, 2018; Guzman *et al*, 2019). The genome‐scale metabolic models can also be used for predicting metabolic gene deletions that couple a desired production trait to growth (Burgard *et al*, 2003; Patil *et al*, 2005). After such model‐guided genome editing adaptive laboratory evolution has successfully been used to improve the growth‐coupled production rates (Burgard *et al*, 2003; Jantama *et al*, 2008; Brochado & Patil, 2013; Jensen *et al*, 2019; Pereira *et al*, 2021). We use these genome‐scale metabolic models to predict environment‐dependence of the coupling between metabolic traits, and that between metabolic traits and the cell fitness. This allowed us to generalize the design of evolution environments and Darwinian selection of target phenotypes.

## Results

### Evolution environment

Consider an application environment, for example, wine must wherein the manifestation of a target metabolic trait, for example, aroma production, is desired. We postulate that improvement of the desired trait in the application environment can be achieved through adaptive evolution in a distinct evolution environment, followed by the return to the application environment. To design the evolution environment, we take advantage of the observation that the coupling between metabolic traits is predictable as couplings between metabolic fluxes and dependent on the nutritional/chemical composition of the environment (Box 1).

Box 1 **Trait‐fitness dependences are predictable as flux couplings.**

The selection acting on a phenotypic trait is the covariance between the trait and the relative fitness, as described by Robertson‐Price identity (Robertson, 1966, 1968; Price, 1970; Rausher, 1992; equation 1).

(1) $$s=\mathrm{cov}\left(w,z\right)$$

where *s* is the selection differential, *w* fitness, and *z* the trait of interest.

When there is genetic covariance between the trait and relative fitness, evolutionary response to selection can occur (equation 2, the secondary theorem of selection).

(2) $$R=s_{g}={\mathrm{cov}}_{a}\left(w,z\right)$$

where *R* is the response to selection with units of the trait and fitness multiplied, *s*_*g*_ is the genetic selection differential, and *cov*_*a*_(*w*,*z*) is the additive genetic covariance.

Equation (2) generalizes to a multivariate form for multiple traits (Rausher, 1992).

(3) $$R={\mathrm{cov}}_{a}\left(w,z\right)$$

We now consider the case of metabolic traits, which can be represented and modeled as a set of metabolic fluxes (net reaction rates). Metabolic trait interdependencies under a given chemical environment can then be predicted using genome‐scale metabolic models as flux couplings (Burgard *et al*, 2004). Two metabolic reactions are coupled if a nonzero flux through one reaction implies a nonzero flux through the other. Flux covariance follows from flux coupling (Heinonen *et al*, 2019; preprint: Pradhan *et al*, 2019; Thommes *et al*, 2019). Importantly for modeling evolutionary adaptation, flux coupling implies genetic dependences between the corresponding enzyme‐coding genes (Notebaart *et al*, 2008).

To predict the relative response of a metabolic trait to selection, we use its coupling to the specific growth rate (proxy for mean fitness). Analogous to the secondary theorem of selection (equation 3), this gives:

(4) $$F_{v}=\frac{v}{\mu }$$

where ***F***_*v*_ is the relative unitless responses of single‐flux metabolic traits to selection, *v* the metabolic fluxes, and *μ* the specific growth rate. Thus, higher the flux per growth unit, stronger the selection.

**Figure 1:**
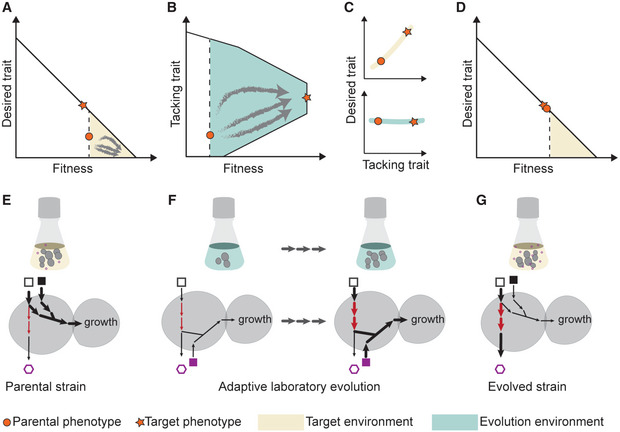
Darwinian selection in an absence of fitness advantage through an evolution environment and a tacking trait Current phenotype is represented with an orange circle, whereas the orange star represents the desired target phenotype.A
In the application environment (yellow), Darwinian selection (gray arrows) enriches cells with fitter phenotypes but with diminished desired trait.B
The tacking trait is chosen to be coupled with fitness in the evolution environment and can therefore be improved through Darwinian selection.C
The tacking trait is also characterized by direct coupling to the desired target trait in the application environment, even though not so in the evolution environment (green).D
Evolved cells with a strengthened tacking trait (through selection in the evolution environment) manifest an improved desired trait in the application environment.E–G
A simple metabolic network illustrating the evolution environment and the tacking trait. The desired trait is the production flux of a compound (open hexagon). The squares depict available nutrients, which differ between the target and evolution environments. The arrows represent metabolic fluxes, the thicker the arrow the higher the flux. The tacking trait (red arrows), which is part of the flux basis of the desired trait, is flux coupled to cell growth flux (i.e., proxy of mean fitness) in the evolution environment. Thus, the tacking trait can be improved through adaptive evolution in the evolution environment. Due to the flux coupling in the application environment, the improved tacking trait leads to the enhanced desired target trait (i.e., increased target compound secretion).

To search for a suitable evolution environment, that is, a defined chemical environment in which the adaptive evolution is to take place, we use the basis provided by the selection response relation (equations 3 and 4). Ideally, the evolution environment would be chosen such that there is a direct selection for the desired trait through flux coupling with the cell growth. This, however, will only rarely be possible as most desired traits, such as metabolite secretion, are in a trade‐off with cell growth due to a competition for metabolic precursors and co‐factors (Jouhten *et al*, 2016; Nielsen & Keasling, 2016; Fig 1A). We therefore aim at growth coupling of a secondary trait, which we term tacking trait. Tacking trait is here defined as a set of fluxes that are flux coupled (Burgard *et al*, 2004) to cell growth in the evolution environment, and with the desired trait in the application environment (Fig 1B). We note that it is neither necessary for the tacking trait to be coupled with the desired trait in the evolution environment, nor it is likely due to the trade‐off with growth. Further, the tacking trait is necessarily a proper subset of fluxes that must increase or decrease for the desired trait enhancement in the application environment. Due to the environment‐dependence of genetic correlations between traits (equation 3), the tacking trait and the evolution environment are intrinsically linked and need to be identified simultaneously.

A desired trait that does not pose a fitness advantage will not be under Darwinian selection in the application environment (Fig 1A). In our strategy, the evolution environment is designed such that the tacking trait becomes flux coupled to mean fitness (Fig 1B), allowing positive selection on *de novo* mutations enhancing the tacking trait. Upon switching to the application environment, in which the tacking trait is flux coupled with the desired trait, the latter is enhanced (Fig 1C and D). To illustrate this strategy, we consider a simple metabolic network (Fig 1E–G). The parental strain is well adapted to channel the nutrients to cell growth and thus produces only a little desired product (Fig 1E). In an appropriately selected evolution environment (Fig 1F), a different set of pathways are flux coupled with growth (Fig 1F). During the adaptive evolution, increased flux through these growth‐coupled pathways is selected for. While there is no increase of production in the evolution environment, the evolved strain exhibits, due to the direct coupling between the tacking and the target trait, improved production in the application environment (Fig 1G).

Under a prolonged cultivation, the desired trait may be negatively selected in the application environment. However, this is not an obstacle for the use of the proposed strategy in, for example, a biotechnological setting. A typical microbiological process involves only a few generations (below 10) and is thus unlikely to diminish the desired trait. The necessary condition will be to maintain and propagate the cell stock in a separate environment (in this case the evolution environment), which is a common practice in microbiology.

### Predicting evolution environment

To predict evolution environments satisfying the conditions laid out above, we devised an algorithm, termed EvolveX, based on genome‐scale metabolic models. The algorithm simultaneously identifies a tacking trait and evaluates the suitability of a set of nutrients for adaptively evolving the tacking trait (evolution environment).

EvolveX consists of four main steps. *Step 1*: For a given desired trait, its flux basis is determined. This is defined as the set of fluxes that must change for the enhancement of the desired trait in the application environment. *Step 2*: For the identified flux basis, a response to selection in the evolution environment is predicted. The subset of the flux basis with nonzero responses to selection forms the tacking trait. Note that, as the covariances of traits may change through evolution (Lande, 1980; Jones *et al*, 2007; Arnold *et al*, 2008), we define the flux basis (*Step 1*) in the ancestral state but predict the response to selection in a state that is expected to be approached during experimental evolution (Ibarra *et al*, 2002; Szappanos *et al*, 2016; McCloskey *et al*, 2018). *Step 3*: A minimum size (cardinality) of the subset of the flux basis having a stronger response to selection in the evolution environment than in the application environment is estimated. *Step 4*: A suitability score of an evolution environment is calculated by combining: (i) results of *Step 2*, indicating the strength of response to selection; (ii) results of *Step 3*, indicating the coverage of flux basis with desired selection; and (iii) the number of chemical components in the evolution environment (lower the number, higher the score). The last criterion is included to discount for the uncertainty in the knowledge of the organism's nutritional preferences. Further details of EvolveX implementation, which accounts for variability in flux estimates and normalizes the score to enable comparison across different growth rates, are provided in Materials and Methods.

### Model‐predicted evolution environments increase aroma production

To experimentally validate the applicability of model‐designed evolution environment, we set to improve secretion of aroma compounds by *Saccharomyces cerevisiae* in wine must. Wine must is characterized by high sugar content and relatively less assimilable nitrogen. As aroma synthesis diverts carbon and nitrogen away from the production of daughter cells, it cannot be adaptively selected in the application environment (wine must). Moreover, while the metabolic pathways that synthesize aroma compounds are known, their regulation is poorly understood, preventing facile engineering of aroma secretion (de Carvalho *et al*, 2017).

We targeted two main groups of aroma compounds: (i) phenylethyl alcohol and its acetate ester, phenylethylacetate, which have a rose and honey scent and raspberry‐like flavor; and (ii) branched‐chain amino acid‐derived higher alcohols (2‐methyl‐1‐butanol and 3‐methyl‐1‐butanol) and their acetate esters (2‐methylbutylacetate and isoamyl acetate; Swiegers *et al*, 2005; Carpena *et al*, 2021), which have a banana and pear scent and fruity flavor. All these aroma compounds derive from amino acids' (L‐phenylalanine and branched chain amino acids) carbon backbones and contain no nitrogen. The flux bases of the target aroma syntheses were defined as a minimum set of fluxes that have to increase for the particular target aroma generation to be enhanced. Similarly, flux bases could include fluxes that should be negatively selected for desired trait development.

**Figure 2:**
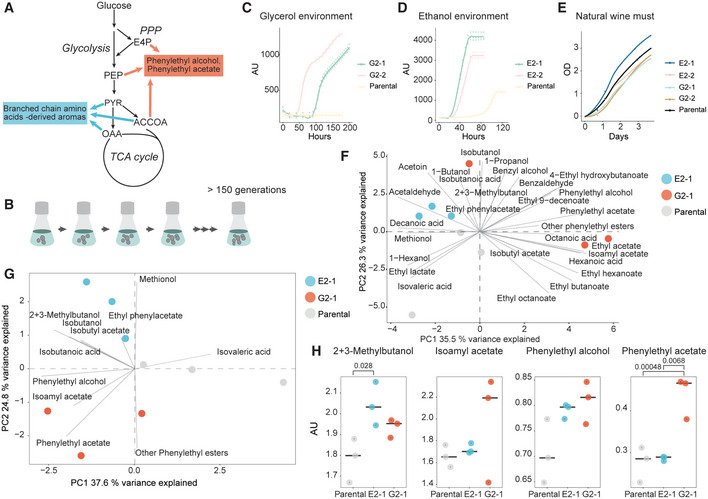
Aroma production changes detected in evolved yeast strains Origin of aroma compounds in the yeast central metabolism: branched‐chain amino acid‐derived compounds (esp. 2‐methyl‐1‐butanol, 3‐methyl‐1‐butanol, isoamyl acetate, and 2‐methylbutylacetate), and aromatic amino acid‐derived compounds (esp. phenylethyl alcohol and phenylethyl acetate). Acetate esters of higher alcohols share an acetyl‐CoA (ACCOA) precursor.Parental wine strain of *S. cerevisiae* was adaptively evolved in both ethanol environment and glycerol environment for over 150 generations.Evolved single colony isolates had improved growth in glycerol environment compared to parental. The growth of isolates G2‐1 and G2‐2 and the parental characterized in three biological replicates as backscattered light (AU—arbitrary units).Evolved single colony isolates had improved growth in ethanol environment compared to parental. The growth of isolates E2‐1 and E2‐2 and the parental characterized in three biological replicates as backscattered light (AU—arbitrary units).Evolved single colony isolates maintained similar to parental growth ability characterized in single biological replicates as carbon loss in natural wine must fermentations.Principal components analysis of quantified 28 volatile aroma compounds in natural wine must fermentations, with the parental (gray) and evolved strains in three biological replicates. Evolved strain from the ethanol evolution environment (ethanol, arginine, glycine), E2‐1, in light blue, and that from the glycerol evolution environment (glycerol, phenylalanine, threonine), G2‐1, in orange.Principal components analysis of aromatic and branched amino acid‐derived volatile compound profiles of natural wine must fermentations, with the parental (gray) and evolved strains (E2‐1 in light blue, G2‐1 in orange) in three biological replicates.Changes in selected aroma compound abundances in wine must fermentations. AU—arbitrary units. E2‐1 (light blue) was selected in the ethanol environment, and G2‐1 (orange) was selected in the glycerol environment. 2 + 3‐methylbutanol (a combined pool of 2‐methyl‐1‐butanol and 3‐methyl‐1‐butanol) and isoamyl acetate (acetate ester of 3‐methyl‐1‐butanol) were the desired target aromas of the ethanol environment, deriving from branched‐chain amino acids. Phenylethyl alcohol and its acetate ester, phenylethyl acetate, were the desired target aromas of the glycerol environment. Medians over three biological replicates are shown with black lines. Significant differences in means (Tukey's test; *n* = 3; *P* value < 0.05) are indicated with *P* values.

To identify a suitable evolution environment for enhancing the target aroma generation and corresponding tacking traits, we assessed all 1,540 combinations of up to three carbon and nitrogen sources, chosen from 22 common constituents of yeast growth media. All combinations were ranked for their suitability for positively selecting the flux bases of the target aroma generation (via the tacking traits) using the EvolveX score (Table EV1). High‐scoring environments were assessed for literature evidence of feasibility of *S. cerevisiae* growth. Two of the high‐scoring environments, which were among the top 20 of 1,171 growth‐supporting solutions, were selected for experimental validation. Evolution environment containing glycerol, phenylalanine, and threonine as sole carbon and nitrogen sources was chosen for phenylethyl alcohol and phenylethylacetate production. In this environment, hereafter called glycerol environment (Fig 2A), 7 fluxes (out of 20 in the flux basis) formed the tacking trait of phenylethyl alcohol and phenylethylacetate production (Table EV2). For branched‐chain amino acid‐derived aromas, ethanol environment (Fig 2A), containing ethanol, arginine, and glycine, was selected for experimental validation. In the ethanol environment, 11 fluxes (out of 44 in the flux basis) formed the tacking trait (Table EV2). The two tacking traits included two common fluxes (transketolase 1, ribulose 5‐phosphate epimerase). However, only eight common fluxes were predicted to be positively selected in the two evolution environments while 57 fluxes were predicted to be selected only in one of the two evolution environments (Table EV3). Notably, the glycerol environment and in the ethanol environment were predicted to expose positive selection on 17 (out of 29) and 20 (out of 44) common fluxes with intuitive control environments glycerol and ammonium and ethanol and ammonium, respectively (Table EV3). Thus, the EvolveX designed glycerol and ethanol evolution environments act as appropriate controls to each other.

In each of the two selected environments, three replicate populations of a diploid wine yeast strain (selected based on capability of growing in both environments) were independently evolved asexually for over 150 generations (Fig 2B). Growth improvement was observed in both evolution environments (Fig 2C and D, Table EV4). In the selected isolates evolved in ethanol environment, an increase in maximum specific growth rate of over two‐fold was estimated (Fig 2D). Aroma production and growth physiology of single colony isolates were assessed in natural wine must fermentations (without any aroma precursor supplementation). All evolved isolates maintained their fermentation performance in the natural wine must (Fig 2E, Table EV5), indicating their suitability for use in wine fermentations.

Mass‐spectrometry analysis of the volatile compounds (28 quantified, Table EV6) in wine must fermentations with the parental strain and evolved isolates provided a view on the changes in volatiles following evolution. In principal components analysis, the strains did not cluster by their history (Fig 2F), supporting that the volatile metabolite production was not universally impacted following laboratory evolution. However, the principal components analysis considering only the aromatic and branched chain amino acid's derived compounds, the aroma profiles clustered by the evolution environment and separately from the parental (Fig 2G). The first principal component (PC1, 37.6% of total variance) distinguished parental from the evolved strains. In accordance with the model predicted overlap of the tacking traits (transketolase and ribulose 5‐phosphate 3‐epimerase fluxes; Table EV2) and fluxes under selection (Table EV3), common separation from the parental aroma profile was expected. Further attesting the model, the isolates selected in the ethanol and glycerol evolution environments were separated mainly by the target aroma compounds (PC2, 24.8% of total variance, Fig 2G and H). While for target aroma compound isoamyl acetate, we could not validate the model predictions (i.e., level similar to parental in fermentations with E2‐1), phenylethylacetate was specifically increased in the wine must fermentations with the isolates selected in the glycerol environment (Fig 2H). Similarly, the combined pool of branched‐chain amino acid‐derived aroma compounds 2‐methyl‐1‐butanol and 3‐methyl‐1‐butanol was increased only for the isolate selected in the ethanol environment. Together, evolved isolates featured increased aroma formation in wine must according to the EvolveX predictions.

### Evolved strains exhibit molecular changes in accord with the model predictions

To understand the genetic basis of the evolved phenotypes, we sequenced the whole genomes of the evolved populations. In addition, we sequenced isolates from the evolved populations at two intermediate stages, an earlier (~100 generations) and a later stage (~214 and ~165 generations for ethanol and glycerol environment, respectively).

**Figure 3:**
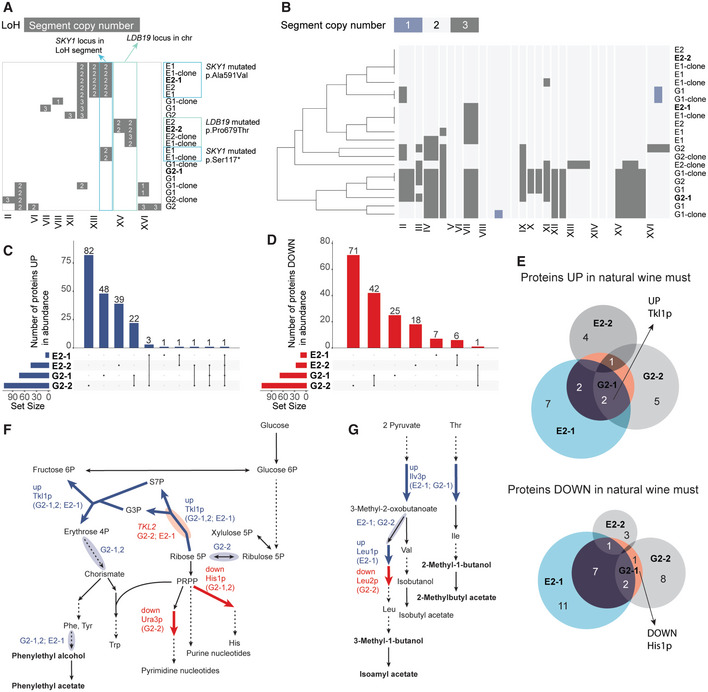
Molecular changes detected in evolved yeast strains Loss‐of‐heterozygosity (LoH) coincided with single nucleotide variants (SNVs, marked on the top and right side of the panel) in evolved populations and clones from the ethanol environment, suggesting the necessity of the SNVs being homozygous for the evolved phenotype. The evolved clones (“‐clone”) and populations are named according to their selection environment: “G”—glycerol selection environment. “E”—ethanol selection environment. The number after the letter stands for the evolution status: 1—the time of first isolation of clones, 2—the time of second isolation of clones. The clones for which we determined protein and transcript alterations are indicated in bold.Evolved populations and clones from the glycerol environment exhibited large copy number variations. Shown are the genome segment copy numbers along the chromosomes (chr). Vertical lines mark ends of contigs.Upset plot of sets of proteins higher in abundance (limma, *n* = 3 (biological replicates), *P* value < 0.01, −1 > log2fc > 1) in the evolved isolates than in the parental strain in the respective evolution environments (G1‐2, G2‐2: glycerol environment; E1‐2, E2‐2 ethanol environment) shows partly shared solutions underlying improved fitness.Upset plot of sets of proteins lower in abundance (limma, *n* = 3 (biological replicates), *P* value < 0.01, −1 > log2fc > 1) in the evolved isolates than in the parental strain in the respective evolution environments (G1‐2, G2‐2: glycerol environment; E1‐2, E2‐2 ethanol environment) shows proportionally large overlaps between the isolates evolved in the same environment.The evolved clones fermenting natural wine must (application environment) revealed both shared and evolution environment‐specific protein abundance changes up and down in comparison to the parental strain (limma, *n* = 3 (biological replicates), *P* value < 0.01, −1 > log2fc > 1). Clones for which we quantified the aroma production are shown in color (E2‐1 in light blue, G2‐1 in orange). Clones from the glycerol environment (G2‐1, G2‐2) featured higher abundance of Tkl1p (transketolase) and lower abundance of His1p (ATP phosphoribosyltransferase).Changes in protein (limma; *n* = 3 (biological replicates), *P* value < 0.01, −1 > log2fc > 1) and transcript abundances (Wald test; *n* = 3 (biological replicates), fdr < 0.05, −1 > log2fc > 1) are centered on the pathways leading to the target aroma compounds phenylethyl alcohol and phenylethyl acetate. The changes consistent with the model predictions are indicated with colored arrows (protein‐level) and clouds around the arrows (transcript‐level).Proteomic and transcriptomic changes in evolved clones, marked as in (F), for pathways leading to the branched chain amino acid‐derived target aroma compounds.

In the case of ethanol environment, copy number variants (CNVs) analysis revealed triplications of chromosome VII in several evolved populations and isolates (Table EV7). Further, recurrently in several populations and clones single nucleotide variants (SNVs) were found in *SKY1*, which encodes a serine/threonine kinase involved in the regulation of polyamine transport (missense p.Ala591Val, frameshift p.Leu64fs, stop gain p.Ser117*; Table EV8). We also observed a loss‐of‐heterozygosity segment in the contig containing the *SKY1* locus. *SKY1* deletion gives yeast tolerance to high spermine concentrations (Erez & Kahana, 2001), a degradation product of arginine, which was one of the three components in the ethanol environment. In clones where *SKY1* mutations were not detected, we found paired missense mutations in genes encoding the ubiquitin ligase Rsp5 (p.Arg355Gly) and its target‐guide and adapter Ldb19 (p.Pro679Thr), which drive the endocytosis of plasma membrane‐localized amino‐acid transporters. Ldb19 variant was accompanied with a loss‐of‐heterozygosity in the contig containing the locus (Fig 3A). Thus, in the ethanol environment, mutations in genes involved in arginine utilization were enriched in accord with the selection regime.

The clones and the populations selected in the glycerol environment had only few SNVs, and no genes showed recurrent SNVs (Table EV8). However, CNVs were prevalent, with multiple triplicated segments observed in several cases (Fig 3B, Table EV7). This extensive variation meant that no particular genes or pathways could be directly linked with either growth or aroma production. Indeed, many of the duplicated genes could be dosage compensated (preprint: Muenzner *et al*, 2022). Therefore, we next resorted to analyzing changes at the transcriptomic and proteomic levels. The evolved cells were characterized both in the application environment (wine must, same batch as was used for determining the aroma profiles) and in their corresponding evolution environments (Tables EV9 and EV10). In all cases, the overlap between transcript‐level and protein‐level changes was below 6%, indicating major role of post‐transcriptional regulation in both the improved aroma generation in wine must and in the improved fitness in the evolution environments.

The fitness improvement in the glycerol environment was associated with a differential abundance of 48 and 78 metabolic enzymes in G2‐1 and G2‐2, respectively (Table EV10). In total, 139 and 224 proteins were found in differential abundance compared to the parental strain (limma; *n* = 3, *P value* > 0.01, −1 > log2fc > 1) in G1‐2 and G2‐2, respectively. Sixty‐six of the proteins were shared (Fig 3C and D) marking the shared solutions in fitness improvement. Many protein down‐regulations were shared between G2‐1 and G2‐2 (Fig 3C). The metabolic enzymes with increased abundance were enriched in respiratory pathways in accord with the strong selection pressure predicted by EvolveX (Table EV3). A significant overlap was found between the enzymes predicted to be positively selected and the proteins present in higher abundance in the clones evolved in the glycerol environment (hypergeometric test; G2‐1 *P* value 0.000022, G2‐2 *P* value 0.0024). The proteins present in higher abundance in G2‐2 overlapped significantly also with the tacking trait (*P* value 0.021). In addition, glycolytic enzymes (Cdc19, Pdc6, and Tdh1) became less abundant in G2‐1, suggesting increased respiratory activity relative to glycolysis.

The fitness improvement in the ethanol environment was also associated with few, focused, enzyme abundance changes (12 in E2‐1 and 31 in E2‐2; limma; *n* = 3, *P* value > 0.01, −1 > log2fc > 1, Table EV10). In total, 19 and 68 proteins were found in differential abundance compared to the parental strain in E1‐2 and E2‐2, respectively. Only eight of these were shared between E2‐1 and E2‐2 (Fig 3C and D), underscoring the multiple evolutionary solutions to fitness improvement. The metabolic enzymes present in higher abundance in E2‐2 significantly overlapped with the enzymes predicted to be positively selected in the ethanol environment (hypergeometric test *P* value 0.050). Consistent with arginine as the nitrogen source in this evolution environment, the changes included decreased abundance of arginine biosynthetic pathway enzymes (Arg1 and Arg8 in E2‐1, and Arg5,7 in E2‐2). Strain E2‐2 further had decreased abundance of proline oxidase, Put1, involved in the utilization of one of the four nitrogen atoms in arginine. Several transporters had higher abundance in E2‐2: arginine permease (Can1), monocarboxylate transporter (Jen1), methionine permease (Mup1), and hexose transporter (Hxt6). The endocytosis of all these transporters is mediated by Rsp5‐Ldb19 (Nikko & Pelham, 2009; Becuwe & Leon, 2014; Guiney *et al*, 2016), which was mutated in the E2‐2. Overall, in both evolution environments, the protein abundance changes were limited to the key growth‐linked pathways predicted by EvolveX—respiration in the glycerol environment, and arginine metabolism in the ethanol environment.

In the application environment (wine must), the improved aroma generation was accompanied by changes in expression of around 50–200 genes (Table EV9). Genes connected to the tacking traits and flux basis were affected, including chorismate synthesis, aromatic amino transferase, and the Ehrlich pathway. In G2‐2, a significant overlap was detected between the corresponding flux basis of the desired trait and the genes found up‐regulated (hypergeometric test; *P* value 0.0052). At protein level, abundance changes (limma; *P* value > 0.01, −1 > log2fc > 1) were observed in 9 to 32 proteins in the evolved isolates (Fig 3E, Table EV10). A few changes in metabolic enzymes centered on the supply of precursors to the target aroma compounds were observed (2 to 10 enzymes, Fig 3F and G). Significant overlap was detected in proteins found in higher abundance in the evolved clones and the tacking traits of the both aroma profiled evolved clones (G2‐1 *P* value 0.011, G2‐2 *P* value 0.017, E2‐1 *P* value 0.046). In E2‐1, also the flux basis excluding the tacking trait overlapped significantly with the proteins in higher abundance than in the parental strain (*P* value 0.0064). All evolved strains except E2‐2 exhibited increased levels of transketolase (Tkl1) consistent with increased precursor supply to aroma biosynthesis as per model prediction. The clones from the glycerol environment showed decreased levels of His1, which competes with Tkl1 for the precursor ribose 5‐phosphate (Fig 3F). Another competing pathway, Orotidine‐5′‐phosphate decarboxylase (Ura3), involved in purine nucleotide synthesis, was also less abundant. In the ethanol environment, increased Tkl1 abundance was accompanied by those of dihydroxyacid dehydratase (Ilv3) and isopropylmalate isomerase (Leu1; Fig 3G). Both Ilv3 and Leu1 are involved in branched‐chain amino acid biosynthesis and higher activities were predicted by the model. Leu2, which follows Leu1 in the leucine biosynthesis pathway, had decreased abundance on one of the clones in accord with the model predictions (Fig 3G). Overall, the protein abundance changes in evolved cells were centered on the aroma synthesis pathways consistent with the model predictions.

## Discussion

The EvolveX algorithm, with roots in the laws of thermodynamics as captured by genome‐scale metabolic models, allowed us to predict the environment‐dependent trait–fitness correlations. Our theory and results thus bring predictive evolution, which has yet mostly based on empirical correlations, in the realm of first‐principles modeling. Previously, adaptive evolution of fitness‐beneficial traits in one niche has been shown to facilitate exaptation, that is, the predisposition to fitness improvement in another niche (Szappanos *et al*, 2016). In contrast, we propose and show that, in an appropriately chosen evolution environment, a trait without a fitness benefit in the application environment can adaptively evolve.

The increased phenylethyl alcohol and phenylethyl acetate generation we observed occurred in an evolution environment containing the direct aroma precursor phenylalanine. In contrast, no target aroma precursor was included in the evolution environment for the branched‐chain amino acid‐derived aroma compounds. This demonstrates the utility of metabolic modeling in identifying nonintuitive evolution environments. While we designed the evolution environments in this study using carbon and nitrogen sources, enzyme inhibitors and substrate analogs can also be used to expand the search space for the evolution environments. Furthermore, while the flux bases in this study included only fluxes that should be positively selected, fluxes that should be negatively selected for developing a desired trait could also be included in the design of evolution environments. The evolution environments were not optimized here also for specificity to changes in the target compounds. While the variance in aromatic and branched‐chain amino acid‐derived aromas (including the target compounds) reflected the evolution environment of the strains, the variance in other volatile compounds can be thought to exemplify both the metabolic couplings and redundant solutions of adaptive evolution. Optimizing the evolution environment choice for the specificity of the desired trait development could be achieved by extending the EvolveX scoring scheme to flux bases that include also fluxes that should not be change in either direction.

A key question for generalization of the proposed strategy is which traits will be accessible through changing the growth environment. By applying the EvolveX algorithm to all reactions in of the yeast metabolism, we predict that 149 reactions can be targeted through environments composed of common nutrients; this coverage can be substantially expanded (to 273) by using enzyme inhibitors in the evolution environment. Instead of enzyme inhibitors, possible metabolic gene deletions/mutants can be used to expand the coverage. EvolveX thus extends the design of growth‐product coupling (Burgard *et al*, 2003; Jantama *et al*, 2008; Brochado & Patil, 2013; Jensen *et al*, 2019; Pereira *et al*, 2021) from genotype‐dependent trait‐fitness dependences to also considering environment‐dependence of the trait selection.

The use of model‐designed evolution environment maintains the key advantage of adaptive laboratory evolution, namely, circumventing the need to know, except for the basic metabolic network structure, the genetic and regulatory basis of the traits of interest. Indeed, the omics analysis of the improved aroma generation traits in our case study revealed complex genotype–phenotype relationships. Improving these traits using rational strain improvement would currently be challenging (Hassing *et al*, 2019). As genome‐scale metabolic models are becoming easier to reconstruct (Pitkanen *et al*, 2014; Machado *et al*, 2018; Wang *et al*, 2018; Seaver *et al*, 2021), our approach can be readily applied to any organism amenable for experimental evolution. Commonly a sufficient quality network is obtained in automatic model reconstruction though the accuracy is the most dependent on the success of protein functional annotation still challenging for less well‐characterized metabolic enzymes. In this study, we used the *S. cerevisiae* reference strain genome‐scale metabolic model to represent our wine yeast parental strain. This demonstrates that the method is not sensitive to differences beyond central pathways when the target compounds originate from the central pathways too. While the choice of the parental wine strain was made based on growth in our selected evolution environments, the EvolveX method is applicable to any strain that can divide in the evolution environment.

The theory of the tacking traits and the evolution environments is generalizable to nonmetabolic traits if the dependency between the fitness and other traits can be quantitatively modeled. This could be done by using, for example, signaling networks. Thus, in a broader view, our theory provides means for understanding complex adaptive processes through systematizing the joint environment‐genetic dependences between fitness and other traits.

## Material and Methods

### Reagents and Tools table

**Table:** 

Reagent/Resource	Reference or Source	Identifier or Catalog Number
**Experimental Models**		
*Saccharomyces cerevisiae*	Lallemand (Blagnac, France)	N/A
**Chemicals, Enzymes and other reagents**		
Arginine	Sigma Aldrich	Cat # A5006
Glycine	Sigma Aldrich	Cat # G7126
Threonine	Sigma Aldrich	Cat # T8625
Phenylalanine	Sigma Aldrich	Cat # P2126
Ethanol	Sigma Aldrich	Cat # 1009861000
Glycerol	Sigma Aldrich	Cat # G5516
Turbo DNAse	Thermo Fisher	Cat # AM2238
RapiGest SF Surfactants	Waters	Cat # 186001861
TMT10plex™ Isobaric Label Reagent	Thermo Fisher	Cat # 90110
**Software**		
Matlab (R2017b v. 9.3.0, R2019b, v. 9.7.0)	https://www.mathworks.com/products/matlab.html	N/A
R (v. 3.6.3, v. 4.0.3, v. 4.1.2)	https://www.r‐project.org	N/A
Python (v. 3.6, v. 3.6.13)	https://www.python.org/	N/A
IBM ILOG CPLEX (v. 12.8.0, v. 12.10.0)	https://www.ibm.com/products/ilog‐cplex‐optimization‐studio	N/A
GATK4 (v. 4.1.0.0)	https://gatk.broadinstitute.org/hc/en‐us	N/A
**Other**		
RNAeasy kit	Qiagen	Cat # 74104
NEBNext® Ultra™ II Directional RNA Library Preparation Kit	New England Biolabs (NEB)	Cat # E7760S
NEBNext Poly(A) mRNA Magnetic Isolation Module	New England Biolabs (NEB)	Cat # E7490L
NEBNext DNA Ultra2 Library Preparation Kit	New England Biolabs (NEB)	Cat # E7103
HiSeq2500 platform	Illumina	N/A
TRACE GC Ultra	Thermo Fisher Scientific	N/A
ISQ mass detector	Thermo Fisher Scientific	N/A
TriPlus autosampler	Thermo Fisher Scientific	N/A
Ultrospec® 2100 UV–Vis spectrophotometer	Biochrom, Harvard Bioscience	N/A
Qubit	Thermo Fisher Scientific	N/A
GeneVac EZ‐2 plus evaporating system	SP Scientific	N/A

### Methods and Protocols

#### EvolveX algorithm

The flux bases of the desired traits were determined as minimum sets of fluxes requiring up‐ or down‐regulation (respective to current metabolic phenotype) for the desired metabolic traits to arise. Such flux sets were determined by solving a mixed‐integer linear programming (MILP) problem after which the required flux change directions either up or down were assigned. The MILP problem was formulated similarly to (Shlomi *et al*, 2005; equation 5).

(5) $$\begin{array}{l} \min\sum_{i=1}^{m}y_{i}\\ s.t.\\ S\cdot v=0\\ v_{\mathrm{lb}}\leq v\leq v_{\mathrm{ub}}\\ v_{\text{trait}}\geq \alpha \cdot v_{\text{trait},\max}\;\alpha \in \left[0,1\right]\\ v_{i}-y_{i}\left(v_{\mathrm{ub},i}-w_{\mathrm{ub},i}\right)\leq w_{\mathrm{ub},i}\\ v_{i}-y_{i}\left(v_{\mathrm{lb},i}-w_{\mathrm{lb},i}\right)\geq w_{\mathrm{lb},i}\;y_{i}\in \left[0,1\right]\\ w_{\mathrm{ub},i}=w_{\max,i}+\delta ¦w_{\max,i}¦+\varepsilon \\ w_{\mathrm{lb},i}=w_{\min,i}-\delta ¦w_{\min,i}¦-\varepsilon \end{array}$$

where ***S*** is the stoichiometric matrix, ***v*** the vector of metabolic fluxes, ***v***_lb_ and ***v***_ub_ the flux lower and upper bounds, respectively, *y*_*i*_ are binary variables representing whether a reaction flux *i* requires up‐ or downregulation for the rise of the target trait *v*_*trait*_ at level α of maximum, *v*_*trait*,*max*_. *w*_*ub,i*_, *and w*_*lb,i*_ are the flux thresholds of wild‐type metabolic phenotype derived from the wild‐type minimum fluxes *w*_*min*,*i*_ and maximum fluxes *w*_*max,i*_ and parameters *δ* and *ε*.

As equally small, alternative, flux sets may exist, they were enumerated by introducing an additional constraint $\sum_{j=1}^{n}y_{j}<n$ rendering previous solutions infeasible.

An evolved metabolic state was predicted by minimizing the total nutrient uptake flux for synthesizing an arbitrary unit of growth while the cells were exposed to inhibitors and metabolic effectors present in the evolution environment (equation 6).

(6) $$\begin{array}{l} \min\;c\cdot v\\ s.t.\\ S\cdot v=0\\ v_{\mu }=10\\ v_{n}\leq -1\;n\in N\\ v_{\mathrm{inh}}=0,\;\mathrm{inh}\in I\\ v_{\mathrm{lb}}\leq v\leq v_{\mathrm{ub}} \end{array}$$

where ***c*** defines the nutrient uptakes possible in the particular evolution environment with −1, *v*_*μ*_ is the growth flux, *v*_*n*_ are the uptake fluxes from environment *N*, and *I* is the set of metabolic reactions inhibited by the inhibitors present in the evolution environment.

The total response to selection of the desired trait (in worst‐case scenario) was predicted as the sum of flux couplings of its flux basis with growth. For the subsets of the flux basis associated with flux change directions up and down, the minimum and maximum growth coupled fluxes, respectively, were summed under the constraint of minimized total nutrient uptake flux for an arbitrary unit of growth (equation 7). Thus, the concept of flux coupling (Burgard *et al*, 2004) was used to identify which reactions in the flux bases are growth coupled in the evolution environment.

(7) $$\begin{array}{l} \min\sum_{¦u¦=1}^{k}v_{¦u¦}+\sum_{¦d¦=1}^{l}\left(-v_{¦d¦}\right)\\ s.t.\\ S\cdot v=0\\ v_{\mu }=10\\ v_{n}\leq -1\;n\in N\\ v_{\mathrm{inh}}=0\;\mathrm{inh}\in I\\ v_{\mathrm{lb}}\leq v\leq v_{\mathrm{ub}}\\ c\cdot v\geq r_{\text{uptake},\max}\\ 0\leq v_{¦u¦}\leq v_{¦u¦,\mathrm{ub}},\;¦u¦\in U,v_{¦u¦,\mathrm{ub}}=\max\left(v_{u,\mathrm{lb}},v_{u,\mathrm{ub}}\right)\\ 0\leq v_{¦d¦}\leq v_{¦d¦,\mathrm{ub}},\;¦d¦\in D,v_{¦d¦,\mathrm{ub}}=\max\left(v_{d,\mathrm{lb}},v_{d,\mathrm{ub}}\right)\\ v_{u}-v_{¦u¦}\leq 0,\;u\in H\\ -v_{u}-v_{¦u¦}\leq 0\\ v_{d}-v_{¦d¦}\leq 0,\;d\in L\\ -v_{d}-v_{¦d¦}\leq 0\\ -v_{d}-M\cdot d_{¦d¦}+v_{¦d¦}\leq 0\\ v_{d}+M\cdot d_{¦d¦}+v_{¦d¦}\leq M,\;d_{¦d¦}\in \left[0,1\right] \end{array}$$

where *r*_*uptake*,*max*_ is the optimal state from equation (3), *H* and *L* are the sets of fluxes (and *k* and *l* the sizes of sets) belonging to the desired trait's flux basis requiring up‐ and down‐regulation, respectively, and *U* and *D* are sets of absolute flux variables representing the fluxes belonging to the desired trait's flux basis requiring up‐ and down‐regulation, respectively. *v*_*¦u¦,ub*_ and *v*_*¦d¦,ub*_ are the upper bounds of the absolute flux variables whose values were derived as maximum absolute value of the flux bounds *v*_*lb*_ and *v*_*ub*_. *M* is a parameter for which a value of 20,000 was used. It is double the maximum flux upper bound. *d*_*¦d¦*_ is a binary variable introduced for each reversible flux belonging to the flux basis of the desired trait and requiring down‐regulation.

The minimum subset size (i.e., worst‐case scenario) of the flux basis of the desired trait having stronger response to selection in the particular evolution environment than in common laboratory growth conditions (or application environment) was estimated under the constraint of minimized total nutrient uptake flux for an arbitrary unit of growth and under the constraint of worst‐case total response to selection of the flux basis of the desired trait (equation 8).

(8) $$\begin{array}{l} \min\sum_{t=1}^{p}b_{t}\\ s.t.\\ S\cdot v=0\\ v_{\mu }=10\\ v_{n}\leq -1\;n\in N\\ v_{\mathrm{inh}}=0\;\mathrm{inh}\in I\\ v_{\mathrm{lb}}\leq v\leq v_{\mathrm{ub}}\\ c\cdot v\geq r_{\max}\\ 0\leq v_{¦u¦}\leq v_{u,\mathrm{ub}}\;¦u¦\in U,v_{¦u¦,\mathrm{ub}}=\max\left(v_{u,\mathrm{lb}},v_{u,\mathrm{ub}}\right)\\ 0\leq v_{¦d¦}\leq v_{d,\mathrm{ub}}\;¦d¦\in D,v_{¦d¦,\mathrm{ub}}=\max\left(v_{d,\mathrm{lb}},v_{d,\mathrm{ub}}\right)\\ v_{u}-v_{¦u¦}\leq 0\;u\in H\\ -v_{u}-v_{¦u¦}\leq 0\\ v_{d}-v_{¦d¦}\leq 0\;d\in L\\ -v_{d}-v_{¦d¦}\leq 0\\ -v_{d}-M\cdot d_{¦d¦}+v_{¦d¦}\leq 0\\ v_{d}+M\cdot d_{¦d¦}+v_{¦d¦}\leq M,\;d_{¦d¦}\in \left[0,1\right]\\ \sum_{¦u¦=1}^{k}v_{¦u¦}+\sum_{¦d¦=1}^{l}\left(-v_{¦d¦}\right)\leq s_{\min}\\ v_{¦u¦}+\left(1+\gamma \right)w_{\max,¦u¦}-v_{\mathrm{ub},¦u¦}\leq \left(1+\gamma \right)w_{\max,¦u¦}\\ -v_{¦d¦}+\left(\gamma -1\right)w_{\min,¦d¦}-v_{\mathrm{lb},¦d¦}\leq \left(\gamma -1\right)w_{\min,¦d¦} \end{array}$$

where *s*_*min*_ is the minimum combined response to selection of the desired trait's flux basis determined above, and *γ* is a threshold parameter for stronger response to selection than in the common laboratory conditions represented by maximum and minimum growth couplings *w*_*max,¦u¦*_ and *w*_*min,¦d¦*_ respectively.

The suitability of an evolution environment for adaptively evolving a desired metabolic trait was evaluated by deriving a weighted sum of (i) the total flux couplings to growth of the desired trait's flux basis, (ii) the minimum subset size of the desired trait's flux basis with higher/lower predicted responses to selection than in common laboratory growth conditions (or application environment), and (iii) the number of chemical components in the evolution environment (equation 9). The lower the score for the evolution environment, the better suited it was considered for adaptively evolving desired traits.

(9) $$\text{score}=\frac{10^{3}\cdot k-s_{\min}}{\left(k+l\right)}+\frac{10^{3}\cdot \left(k+l-b_{\min}\right)}{\left(k+l\right)}+z$$

where *b*_*min*_ is the minimum subset size (i.e., worst‐case scenario) of the desired trait's flux basis having stronger response to selection than in common laboratory growth conditions (or application environment), and *z* is the number of components in the particular evolution environment.

#### Model simulations

*Saccharomyces cerevisiae* consensus genome‐scale metabolic model v. 7.6 (Herrgard *et al*, 2008; Aung *et al*, 2013) was used with few revisions including augmenting the model with missing Ehrlich pathway (Hazelwood *et al*, 2008) reactions and conditional constraints for predicting parental and evolved flux states. For implementing the model revisions, Matlab R2017b v. 9.3.0 and Cobra toolbox v.3.0 (cloned 29.03.2018; Heirendt *et al*, 2019) were used. EvolveX algorithm was implemented and run in Matlab R2017b v. 9.3.0 with IBM ILOG CPLEX v. 12.8.0 functions “cplexlp” and “cplexmilp.” The prediction of response to selection in environments with glycerol and ammonium and ethanol and ammonium was performed using Matlab R2019b v. 9.7.0 with IBM ILOG CPLEX v. 12.10.0. For the determination of reaction coverage with positive response to selection (response to selection as above difference to synthetic defined medium with glucose and ammonium as the sole carbon and nitrogen sources, respectively, under carbon catabolite repressed respiration > 0.001) during respirative metabolism in environments of all combinations of two or three common nutrients out of 22 in total with or without seven inhibitors of eukaryotic central metabolic enzymes and a transporter (i.e., inhibitors of glucose 6‐phosphate isomerase, hexokinase, phosphoglucomutase, succinate dehydrogenase, pyruvate transporter, ATP synthase, pyruvate kinase) EvolveX response to selection prediction was implemented and run in *Python* v. 3.6.13 importing packages *cplex* v. 12.8.0.0, *cobra* v. 0.22.0, *numpy* v. 1.19.5, and *pandas* v. 1.1.5.

#### Strains and culture media

Both evolution environments were created by modifying the defined minimal yeast growth medium by Verduyn *et al* (1992). The evolution environment media contained 6.6 g/l K_2_SO_4_, 3 g/l KH_2_PO_4_, 0.5 g/l (MgSO_4_)7H_2_O, and the vitamins and trace elements as in Verduyn *et al* (1992). The vitamin solution was composed of 50 mg/l of d‐biotin, 200 mg/l of para‐amino benzoic acid, 1.0 g/l of nicotinic acid, 1.0 g/l of Ca‐pantothenate, 1.0 g/l of pyridoxine‐HCl, 1.0 g/l of thiamine‐HCl, and 25 mg/l of *myo*‐inositol and the trace minerals solution of 3 g/l of FeSO_4_·7H_2_O, 4.5 g/l of ZnSO_4_·7H_2_O, 4.5 g/l of CaCl_2_·6H_2_O, 0.84 g/l of MnCl_2_·2H_2_O, 0.3 g/l of CoCl_2_·6H_2_O, 0.3 g/l of CuSO_4_·5H_2_O, 0.4 g/l of NaMoO_4_·2H_2_O, 1 g/l of H_3_BO_3_, 0.1 g/l of KI, and 15 g/l of Na_2_EDTA·2H_2_O. The nitrogen and carbon sources were specific for a particular evolution environment medium. The ethanol environment contained 7.5 g/l of ethanol, 1.7 g/l of arginine, and 0.8 g/l of glycine. The glycerol environment contained 5 g/l of glycerol, 5 g/l of phenylalanine, and 1.2 g/l of threonine. The pH of the media were set to 6 and the media were sterile filtered.

Initially, 12 different *S. cerevisiae* wine strains (commercial and vineyard isolates) were tested for their ability to grow in the two selected evolution environments. The majority of the strains were able to grow sufficiently in ethanol environment after 4 days of culture; however, only four strains were able to grow in glycerol environment even after a week of culture had passed. We selected as a parental strain for adaptive laboratory evolution a commercial diploid wine strain *S. cerevisiae* obtained from Lallemand, which was able to grow in both evolution environments.

Precultures and cells for genomic DNA extraction were grown overnight in rich medium (YPD) containing 10 g/l of yeast extract, 20 g/l of peptone, and 20 g/l of glucose sterilized through autoclaving. Single strain isolations were performed using YPD or synthetic wine must mimicking medium (WMM) plates containing 2% agar as solidifying agent. The WMM composition was 100 g/l of glucose, 100 g/l of fructose, 5 g/l of citric acid, 0.5 g/l of malic acid, 0.25 g/l of MgSO_4_, 0.75 g/l of KH_2_PO_4_, 0.5 g/l of K_2_SO_4_, 0.155 g/l of CaCl_2_, 0.2 g/l of NaCl, 0.15 g/l of NH_4_Cl, 2 ml/l of anaerobic factors (1.5 g/l of ergosterol, 0.5 g/l of oleic acid, 50 g/l of Tween 80, and 5 g/l of ethanol), 3.5 ml/l of amino acid solution (1.95 g/l of tyrosine, 17.5 g/l of tryptophan, 3.25 g/l of isoleucine, 4.42 g/l of aspartic acid, 11.95 g/l of glutamic acid, 44.5 g/l of arginine, 4.8 g/l of leucine, 7.54 g/l of threonine, 1.82 g/l of glycine, 49.92 g/l of glutamine, 14.56 g/l of alanine, 4.42 g/l of valine, 3.12 g/l of methionine, 3.77 g/l of phenylalanine, 7.8 g/l of serine, 4.57 g/l of histidine, 2.11 g/l of lysine, 2.7 g/l of cysteine, and 59.93 g/l of proline), 5 ml/l of vitamin solution (2 g/l of *myo*‐inositol, 0.15 g/l of Ca‐pantothenate, 0.025 g/l of thiamine‐HCl, 0.2 g/l of nicotinic acid, 0.036 g/l of pyridoxine‐HCl, and 0.03 g/l of biotin), and 5 ml/l of trace elements solution [4 g/l of MnSO_4_, 4 g/l of ZnSO_4_, 1 g/l of CuSO_4_, 1 g/l of KI, 0.4 g/l of CoCl_2_, 1 g/l of H_3_BO_3,_ and 1 g/l of (NH_4_)Mo_7_O_24_] and the pH was set to 3.3.

#### Adaptive laboratory evolution

The adaptive laboratory evolution experiment was initiated by inoculating both evolution environments in triplicate from overnight single colony precultures of the parental *S. cerevisiae* strain on YPD to starting OD_600_ of 0.2. The adaptive laboratory evolution was performed for the triplicate lineages on each evolution environment as a serial transfer experiment with 7 ml of liquid cultures in 50‐ml shake flasks at 30°C with shaking at 180 rpm. The shake flasks were capped with cotton plugs for enhanced aeration. When culture turbidity was visually observed, the lineages were transferred to fresh medium initially to a starting OD_600_ of 0.2 and later, after the growth had improved to OD_600_ of 0.1, measured with a spectrophotometer (Ultrospec 2100, Biochrom). Intermediate lineage samples were collected to 30% w/v glycerol and stored at −80°C.

The adaptive laboratory evolutions were initially performed for approximately 107 generations in ethanol environment and 100 generations in glycerol environment after which single colonies were picked and the isolates performing the best in the corresponding evolution environment characterized as described below. The number of generations was calculated back using the following formula [Log_10_(*A*_*f*_/*A*_*i*_)]/0.3, where *A*_*f*_ is the OD_600_ before the transfer and *A*_*i*_ is the OD_600_ that was initially inoculated. A second round of adaptive laboratory evolution was initiated with the individual strains when they showed positive aroma profile development in the characterization (two lineages in ethanol environment, one lineage in glycerol environment), and with the last population stocks in case aroma profile improvement was not yet observed (one lineage in ethanol environment, two lineages in glycerol environment). The second round of evolution was continued approximately for additionally 97 and 65 generations in evolution environments with ethanol and glycerol, respectively. Then, single colonies were again picked and the isolates performing the best in the corresponding evolution environment characterized as described below.

#### Characterization of evolved strains

For discarding nongenetic adaptation and for ensuring wine fermentation performance, single colonies were picked from the evolved lineages following growth on WMM + 2% agar plates for 48 h. Nine single colonies were isolated from each lineage and cultured overnight in liquid cultures on WMM. From these overnight cultures, stocks were prepared to 30% w/v Glycerol and stored at −80°C. The overnight cultures on WMM were also used to inoculate corresponding evolution environment as in adaptive laboratory evolution. Cell growth was monitored with turbidity (OD_600_) measurements. One arbitrarily selected strain from each evolution environment was characterized in wine fermentation mimicking conditions.

Yeasts were maintained at 4°C on YPD plates (2% glucose, 2% peptone, 1% yeast extract, and 2% agar), or as glycerol stocks at −80°C. Inocula were grown on YPD for 48 h at 25°C, washed and suspended in water.

Natural white must from the 2017 harvest was kept frozen. This must contained 215.8 g/l of sugar, density 1,088.3 g/l. Enough volume for the experiment was thawed and pasteurized. In the pasteurization process the natural must was heated to 105°C and then it was allowed to cool down inside the closed autoclave. The same batch of natural white must was used for the aroma profiling and RNA‐sequencing and proteomics experiments.

Aliquots of 25 ml of pasteurized grape must were inoculated at 0.2 final OD_600_. Fermentation was carried at 25°C in Falcon tubes (50 ml nominal volume), capped with air locks, and performance monitored by weight loss. After 8 days, weight was constant, and samples were centrifuged and supernatants were kept frozen for HPLC analysis of sugars and the main fermentation byproducts (i.e., ethanol, glycerol, acetate) of the cultures (Table EV5) and GC–MS analysis of volatile compounds (Table EV6). All experiments were performed in triplicate.

#### Determination of metabolite concentration

The concentration of glucose, fructose, glycerol, ethanol, and acetic acid was determined using a Surveyor Plus liquid chromatograph (Thermo Fisher Scientific, Waltham, MA) equipped with a refraction index and a photodiode array detector (Surveyor RI Plus and Surveyor PDA Plus, respectively) on a 300 × 7.7 mm PL Hi‐Plex H+ (8 μm particle size) column (Agilent Technologies, Santa Clara, CA) and 4 × 3 mm ID Carbo‐H guard (Phenomenex, Torrance, CA). The column was maintained at 50°C and 1.5 mM of H_2_SO_4_ were used as the mobile phase at a flow rate of 0.6 ml/min. Prior to injection in duplicate, the samples were filtered through 0.22 μm pore size nylon filters (Micron Analitica).

#### Analysis of volatile compounds

Samples for gas chromatography–mass spectrometry (GC–MS) analysis contained 2,000 μl of sample, 1 g of NaCl, and 20 μl of internal standard, in 20‐ml flasks. Internal standard contained 1,000 ppm each of 4‐methyl 2‐pentanol and heptanoic acid, and 100 ppm 1‐nonanol, in water, prepared from 10,000 ppm individual solutions in ethanol. Sample was preincubated for 10 min at 45°C, followed by 30 min at 45°C with 50/30 μm DBV/CAR/PDMS SPME fiber (Stableflex, SUPELCO, Bellefonte, PA). Fiber was desorbed for 5 min at 250°C.

GC–MS was carried out in a Thermo TRACE GC Ultra apparatus coupled to a Thermo ISQ mass detector, equipped with a Thermo TriPlus autosampler. Gas chromatography was carried in a Thermo Scientific fused‐silica capillary column TG‐WAXMS A (30 m long; 0.25 mm OD; 0.25 μm film thickness). Chromatographic conditions were as follows: 5 min at 40°C, 3°C/min up to 200°C, 15°C/min up to 240°C, 10 min at 240°C. Helium was used as carrier gas at a flow rate of 1 ml/min, operating in split mode (ratio 30). Total analysis time was 71 min. Detection was performed with the mass spectrometer operating in the Full Scan mode (dwell time 500 ms), with 70 eV ionization energy, and source and quadrupole temperatures of 250°C. Detection was stopped during the time interval for ethanol elution. Peak identification was made by comparison of ion spectra with NIST mass spectral library. For each compound, including internal standards, the sum of the areas of the peaks of selected characteristic ions was obtained. Area of each compound was referred to one selected internal standard.

#### Whole genome sequencing of populations and isolates

Genomic DNA was extracted from parental strains grown in YPD, lineages grown in evolution environments, and single colony isolates grown in YPD using Phenol–Chloroform‐based extraction. Specifically, total volume of 7 ml overnight cultures was centrifuged at 1,100 *g* for 3 min and the pellets were washed with sterile ddH_2_O. The cells were resuspended in 2 ml of TrisEDTA solution (0.1 M Tris and 0.1 M EDTA) and transferred to Eppendorf tubes, with 1.5 U lyticase. The pellets were then incubated at 37°C for 30 min. Next the spheroplasted cells were centrifuged at 550 *g* for 2 min (Eppendorf centrifuge), the supernatant was removed and the cells were resuspended in 400 μl of breaking buffer which contained 10 mM of Tris, 1 mM of EDTA, 100 mM of NaCl, 2% Triton X‐100, and 1% SDS. The cell suspensions were transferred to FastPrep Cap tubes with 200 μl of glass beads (400 nm acid washed, Sigma) and the cells were broken with three rounds of bead beating at 4.5 Mhz/s for 20 s with 1 min cooling intervals or through vortexing only. The cell lysates were transferred to a new tube that contained 400 μl of phenol–chloroform/isoamyl alcohol and 400 μl of TE buffer (Tris 50 mM, EDTA 20 mM) and were centrifuged briefly until an emulsion was formed. The emulsions were centrifuged at 20,000 *g* for 5 min at room temperature. The aqueous phase of each tube was transferred to a new Eppendorf, it was mixed with 1 ml of cold 100% ethanol and incubated at room temperature for 10 min to help precipitation. In the next step, the tubes were centrifuged at 20,000 *g* for 5 min at room temperature, the ethanol was removed and the DNA pellet was resuspended at 400 μl of TE buffer with 2 μl of RNAse solution (20 mg/ml) and incubated for 15 min at 37°C, followed by a second incubation step at 65°C for 15 min, in order to deactivate the RNAse. The DNA solution was mixed with 400 μl of phenol–chloroform/isoamyl alcohol and the extraction step was performed again as described above. DNA was precipitated from the aqueous phase with 1 ml of cold 100% ethanol and centrifugation at 20,000 *g* for 5 min at room temperature. The pellet was left to dry for 30 min at 55°C, next was resuspended with 50 μl of H_2_O and was left overnight at 4°C for the pellet to dissolve completely.

The quality of the extracted DNA was evaluated with electrophoresis in a 1% [w/v] agarose gel. DNA concentrations were measured using a Qubit (Thermo Fisher Scientific, USA). Equal amounts of DNA from all samples were used for library preparation, which was done with the NEBNext DNA Ultra2 Library Preparation Kit (New England Biolabs). The preparation of the library was performed on an automated liquid handling system (Hamilton Robotics), the quality of the library was tested on a 2100 BioAnalyzer (Agilent Technologies), and the DNA concentration was measured using a Qubit. Sequencing was performed at the Genomics Core Facility (EMBL Heidelberg) with use of the HiSeq2500 platform (Illumina, San Diego, USA) and the run produced 250 bp paired‐end reads.

The sequenced samples are listed in Table EV11, and the raw reads are deposited in ENA database (https://www.ebi.ac.uk/ena/browser/home) in study PRJEB40761 with accession numbers ERS5457098 and ERS5290477–ERS5290502 for the parental and evolved samples and in study PRJEB41108 with accession numbers ERS5293678–ERS5293725 for the Panel of Normals (PoN)‐samples.

#### Whole genome sequence data analysis

The quality of the obtained reads was checked using Fastqc v. 0.11.4 (Andrews, 2010). Adapter removal and low‐quality read filtering was performed using cutadapt v. 1.9.1 (Martin, 2011). The trimmed reads were aligned to *S. cerevisiae* EC1118 reference genome (Novo *et al*, 2009) with the Burrows‐Wheeler Aligner v. 0.7.12 *mem* (Li & Durbin, 2009) using default parameters. The alignments were processed (added read groups, sorted, reordered, and indexed) and duplicate reads were marked using Picard Tools v. 1.129 (Van der Auwera *et al*, 2013; preprint: Poplin *et al*, 2018). Single nucleotide variant (SNV), and insertion–deletion (indel) variant calling was performed against the parental sample with GATK4 v. 4.1.0.0 (Van der Auwera *et al*, 2013; preprint: Poplin *et al*, 2018) *Mutect2* using the *S. cerevisiae* EC1118 as the reference and default parameters. PoN for the variant calling was compiled of 47 wild‐type *S. cerevisiae* strains (winery isolates and commercial wine strains, including the parental) sequenced on the same platforms as the actual samples. Variant calling was first performed for the wild‐type strains by running *Mutect2* in tumor‐only mode and then the panel of normal was created with GATK4 v. 4.1.0.0 (Van der Auwera *et al*, 2013; preprint: Poplin *et al*, 2018) *CreateSomaticPanelOfNormals*. The variant calls were filtered using GATK4 v. 4.1.0.0 (Van der Auwera *et al*, 2013; preprint: Poplin *et al*, 2018) *FilterMutectCalls* using default thresholds and by keeping the variants at PoN sites.

Copy number variant (CNV) analysis was performed on read counts and B‐allele frequencies (baf) using GATK4 v. 4.1.0.0 (Van der Auwera *et al*, 2013; preprint: Poplin *et al*, 2018) tools. First, the read counts were binned for 1,000 bp intervals with *CollectReadCounts*. These read counts were denoised with *DenoiseReadCounts* using the parental sample as a matched normal. The allelic counts were collected using *CollectAllelicCounts* and combined with the binned read counts for modeling the CNV segments using *ModelSegments* with number‐of‐changepoints‐penalty‐factor of eight. CNVs were called using *CallCopyRatioSegments*. In major copy number aberrations, the default centralization of the log_2_ copy ratios to median across all contigs, misplaced the zero level to deviate from the conserved copy number. As there were major differences in the copy number aberrations between the samples, the copy ratios were re‐normalized to the level of FN393086.1 contig with conserved copy number across samples. The contig was identified using the minor allele frequencies and the copy ratio differences between modeled segments. After the re‐normalization, CNV's were called for segments longer than 10 kb with −0.6 ≤ log2 copy ratio ≥ 0.3. Each CNV call was evaluated against baf data. The calls were corrected conservatively if the baf data did not support the log2 copy ratio call. The population sample calls were corrected only if the call could not be explained even by partial loss‐of‐heterozygosity, identified as baf zero/one segments. The log2 copy ratios of modeled segments, their copy number calls, and the loss‐of‐heterozygosity identified in the samples are provided in Table EV7. Heatmap of contig median copy ratios was plotted using R v. 4.0.3 (R Core Development Team, 2021) *gplots* package v. 3.1.1 (Warnes *et al*, 2020).

#### Small scale fermentation of natural wine must (microvinification) for transcriptomics and proteomics analysis

A single colony of the parental strain, two evolved isolates originating from the ethanol environment and two evolved isolates originating from the glycerol environment were grown overnight in 50 ml Falcon® tubes with 15 ml of YPD. The overnight grown cells were washed three times with PBS and diluted to an initial OD_600_ of 0.1 in 55 ml of natural white must from the 2017 harvest (see above, the same natural white must batch used as for aroma profiling). For the microvinification process, 50‐ml Erlenmeyer flaks were used, filled to the maximum, in order to create microanaerobic conditions. Maintaining the anaerobic conditions meant that the growth could not be estimated based on changes in the optical density, but it was correlated with the observed weight loss, which occurs from the release of CO_2_, the end product of carbon metabolism. Release of CO_2_ is possible through a small needle which is pierced through rubber plugs, which in turn were sterilized and used to seal the Erlenmeyer flaks, while a small piece of gauge prevents anything from the environment to fall inside the flask through the needle. The growth stage of the cultures was estimated based on weight loss which correlates to the consumption of glucose and release of CO_2_ as suggested by (Harsch *et al*, 2010). For this reason, the initial weight of the cultures was measured and followed once every day until no more weight loss was observed, at which point the cultures had entered stationary phase. After the establishment of the growth kinetics with weight loss, same cultures as described above were prepared, weight loss was once again followed and the cells were harvested at mid exponential phase for RNA‐sequencing and proteomics analysis.

#### RNA‐sequencing sample preparation and data analysis

All the RNA samples were prepared according to the following procedure. Total volume of 20 ml from each culture was transferred to a 50 ml Falcon® filled with ice and was immediately centrifuged at 1,100 *g* for 3 min at 4°C (Eppendorf centrifuge). Next the supernatant was discarded and the cell pellet was snap frozen into liquid nitrogen and stored at −80°C, until the extraction. Total RNA from the pellets was extracted with the RNAeasy kit (Qiagen) according to the manufacturer's recommendations. In brief, 594 μl of RTL buffer plus 6 μl of β‐Mercaptoethanol were used to resuspend the frozen cell pellet which was left on ice. The resuspended cells were transferred to an ice cold FastPrep Cap tube which contained 600 μl glass beads (400 nm acid washed, Sigma). The cells were then lysed with 2 cycles of bead beating, each cycle lasted 10 s at 6 Mz/s with 15 s cooling interval. Cell lysates were transferred to a new tube and were centrifuged for 2 min at full speed (Eppendorf centrifuge) and the supernatant was carefully mixed with 1 volume of 70% HPLC‐grade ethanol. Next, the total volume of the sample was transferred to an RNAeasy column and the manufacturer's instructions were followed. Total RNA was eluted with 60 μl of RNAse free water and Turbo DNAse (Invitrogen Ambion) was used to digest leftover DNA according to the manufacture instruction. Finally, one more step of RNA clean‐up was performed with the same kit.

RNA library was prepared using the NEBNext® Ultra™ II Directional RNA Library Preparation Kit for Illumina: polyA transcripts capture. Briefly, barcoded stranded mRNA‐seq libraries were prepared from high‐quality total RNA samples (~200 ng/sample) using the NEBNext Poly(A) mRNA Magnetic Isolation Module and NEBNext Ultra II Directional RNA Library Prep Kit for Illumina (New England Biolabs (NEB), Ipswich, MA, USA) implemented on the liquid handling robot Beckman i7. Obtained libraries that passed the QC step were pooled in equimolar amounts; 2 pM solution of this pool was loaded on the Illumina sequencer NextSeq 500 and sequenced uni‐directionally, generating ~500 million reads, each 85 bases long.

The quality of the obtained RNA‐sequencing reads was assessed and summarized with Fastqc v. 0.11.5 (Andrews, 2010). Adapter trimming, to remove the standard lllumina TrueSeq Index adapter sequences, was performed using cutadapt v. 2.3 (Martin, 2011). Subsequently, quality read filtering and trimming was performed with FaQCs v. 2.08 (Lo & Chain, 2014), with the following parameters: *‐q 20 ‐min_L 30 ‐n 3*. After trimming and filtering steps total number of reads were, in average, 31 million. Trimmed reads were then aligned to the reference genome of *S. cerevisiae* EC1118 (EnsemblFungi: annotation number GCA_000218975.1) using STAR v. 2.5.2a (Dobin *et al*, 2013). On average, 85% of reads uniquely mapped to an annotated feature in the reference genome. Only uniquely mapped reads were then used to generate the gene level count tables with HTSeq v. 0.9.1 (Anders *et al*, 2015). Statistical analysis was performed with R v. 3.6.1 (R Core Development Team, 2019). Differential expression analysis, including multiple testing correction and independent filtering, was performed with Bioconductor package: DESeq2 v. 1.12.0 (Love *et al*, 2014). False discovery rate (fdr) was calculated with fdrtool v. 1.2.15 (Strimmer, 2008) using the raw *P* values returned by DESeq2. Genes with fdr < 0.05 and log2 fold change (log2fc) > 1 or < −1 were considered as significantly differentially expressed. Unless specified, all packages were used with default parameters.

#### Proteomics sample preparation and data analysis

For the extraction of total proteome 10 mL of each culture were transferred into ice‐cold 15‐ml Falcon® tubes which were centrifuged immediately at 1,100 *g* for 3 min at 4°C (Eppendorf centrifuge). The supernatant from the centrifugation was discarded and the cell pellets were washed once with 1 ml of cold PBS buffer. The washed pellets were snapped frozen with liquid nitrogen and stored at −80°C. For the extraction, the cell pellets were lysed with 0.1% RapiGest (Waters) in 100 mM of ammonium bicarbonate, followed by mechanical disruption with three rounds of sonication (1 cycle: 10 s sonication and 10 s rest on ice per round). Sonication was followed by 2 cycles of bead beating (200 μl glass beads, 400 nm acid washed, Sigma), each cycle lasting 20 s at 4 Mz/s with 1 min cooling intervals between the cycles.

Reduction of disulfide bridges in cysteine containing proteins was performed with dithiothreitol (56°C, 30 min, 10 mM in 50 mM HEPES, pH 8.5). Reduced cysteines were alkylated with 2‐chloroacetamide (room temperature, in the dark, 30 min, 20 mM in 50 mM HEPES, pH 8.5). Samples were prepared using the SP3 protocol (Hughes *et al*, 2019) and trypsin (sequencing grade, Promega) was added in an enzyme to protein ratio 1:50 for overnight digestion at 37°C. Peptides were labeled TMT10plex (Werner *et al*, 2014) Isobaric Label Reagent (Thermo Fisher) according the manufacturer's instructions. For further sample clean up an OASIS® HLB μElution Plate (Waters) was used. Offline high pH reverse phase fractionation was carried out on an Agilent 1200 Infinity high‐performance liquid chromatography system, equipped with a Gemini C18 column (3 μm, 110 Å, 100 × 1.0 mm, Phenomenex; Reichel *et al*, 2016), resulting in 12 fractions.

After fragmentation, the peptides were separated using an UltiMate 3000 RSLC nano LC system (Dionex) fitted with a trapping cartridge (μ‐Precolumn C18 PepMap 100, 5 μm, 300 μm i.d. × 5 mm, 100 Å) and an analytical column (nanoEase™ M/Z HSS T3 column 75 μm × 250 mm C18, 1.8 μm, 100 Å, Waters). Trapping was carried out with a constant flow of trapping solution (0.05% trifluoroacetic acid in water) at 30 μl/min onto the trapping column for 6 min. Subsequently, peptides were eluted via the analytical column running solvent A (0.1% [v/v] formic acid in water) with a constant flow of 0.3 μl/min, with increasing percentage of solvent B (0.1% [v/v] formic acid in acetonitrile) from 2 to 4% in 4 min, from 4 to 8% in 2 min, then 8 to 28% for a further 37 min, in another 9 min. From 28 to 40%, and finally 40–80% for 3 min followed by re‐equilibration back to 2% B in 5 min. The outlet of the analytical column was coupled directly to an Orbitrap QExactive™ plus Mass Spectrometer (Thermo) using the Nanospray Flex™ ion source in positive ion mode.

The peptides were introduced into the QExactive plus via a Pico‐Tip Emitter 360 μm OD × 20 μm ID; 10 μm tip (New Objective) and an applied spray voltage of 2.2 kV. The capillary temperature was set at 275°C. Full mass scan was acquired with mass range 375–1,200 m/z in profile mode with resolution of 70,000. The filling time was set at maximum of 100 ms with a limitation of 3 × 10^6^ ions. Data‐dependent acquisition (DDA) was performed with the resolution of the Orbitrap set to 17,500, with a fill time of 50 ms and a limitation of 2 × 10^5^ ions. A normalized collision energy of 32 was applied. Dynamic exclusion time of 20 s was used. The peptide match algorithm was set to “preferred” and charge exclusion “unassigned,” charge states 1, 5–8 were excluded. MS data were acquired in profile mode.

The acquired data were processed using IsobarQuant (Franken *et al*, 2015) and Mascot v. 2.2.07. A Uniprot *S. cerevisiae* proteome database (UP000002311) containing common contaminants and reversed sequences was used. The search parameters were the following: Carbamidomethyl (C) and TMT10 (K; fixed modification), Acetyl (N‐term), Oxidation (M), and TMT10 (N‐term; variable modifications). A mass error tolerance of 10 ppm was set for the full scan (MS1) and for MS/MS (MS2) spectra of 0.02 Da. Trypsin was selected as protease with an allowance of maximum two missed cleavages. A minimum peptide length of seven amino acids and at least two unique peptides were required for a protein identification. Differential abundance was performed with limma (Ritchie *et al*, 2015). Protein with *P* value < 0.01 and −1 > log2fc > 1 were considered significantly differentially abundant. GO‐process enrichments were determined using https://www.yeastgenome.org/goTermFinder with the metabolic enzymes annotated in *S. cerevisiae* consensus genome‐scale metabolic model v. 7.6 (Herrgard *et al*, 2008; Aung *et al*, 2013) at *P* value 0.1. Hypergeometric test was performed to compute the significance of the overlaps of the model‐predicted flux bases, tacking traits, and fluxes positively selected in a particular evolution environment and the transcripts found in higher abundance (fdr < 0.05, log2fc > 1) and proteins found in higher abundance (*P* value < 0.01, log2fc > 1) in the evolved clones than in parental strain. The test was performed using R v. 4.1.2 function *phyper* (R Core Development Team, 2020) and overlaps with *P* value < 0.05 were reported.

#### Data analysis and visualization

Growth profiles were analyzed by first smoothing the log‐transformed data (i.e., backscattered light or CO_2_ loss) using univariate spline with polyorder = 3. Then, the maximum growth rate was calculated as the maximum value of the smoothed curve derivative. The highest biomass level was calculated as the maximum of the Euler's number elevated to the smoothed values over time. Finally, the lag phase was estimated as the time before 10 or 25% (glycerol environment) of the maximum smoothed value is reached after having passed the minimum level of the smoothed curve. This growth profile analysis was performed using python 3.6 and the *UnivariateSpline* function from the *scipy* library v. 1.1.0.

Principal component analysis was performed using R v. 4.0.3 (R Core Development Team, 2021) and *factoextra* package v. 1.0.7 (Kassambara & Mundt, 2017). Data processing was performed using *readr* package v. 1.4.0 (Wickham & Hester, 2020), *dplyr* package v. 1.0.2 (Wickham *et al*, 2020) and *tidyr* package v. 1.1.2 (Wickham, 2020). For statistical plotting *ggplot2* package v. 3.3.2 was used, the euler diagrams were generated using *eulerr* package v. 6.1.0 (Larsson, 2020), CNV heatmap was plotted using heatmap.2 function from *gplots* package v. 3.1.1 (Warnes *et al*, 2020), using *hclust* as the clustering function with *complete linkage* method, and the color schemes were obtained from RColorBrewer package v. 1.1–2 and v. 1.1–3 (R v. 4.1.2; Neuwirth, 2014), and *ggsci* package v. 2.9 (Xiao, 2018). Tukey's test was performed with R v. 4.1.2 function *TukeyHSD*.

## Author contributions

**Paula Jouhten:** Conceptualization; software; formal analysis; investigation; visualization; methodology; writing – original draft; writing – review and editing. **Dimitrios Konstantinidis:** Formal analysis; investigation. **Filipa Pereira:** Formal analysis; investigation. **Sergej Andrejev:** Resources. **Kristina Grkovska:** Investigation. **Sandra Castillo:** Software. **Payam Ghiaci:** Investigation. **Gemma Beltran:** Resources. **Eivind Almaas:** Conceptualization; supervision. **Albert Mas:** Resources; supervision. **Jonas Warringer:** Resources; supervision. **Ramon Gonzalez:** Resources; supervision. **Pilar Morales:** Formal analysis; investigation. **Kiran R Patil:** Conceptualization; resources; visualization; methodology; writing – original draft; writing – review and editing.

## Disclosure and competing interests statement

PJ and KRP are co‐inventors of a pending patent application of EvolveX algorithm.

## Supporting information



Table EV1

Table EV2

Table EV3

Table EV4

Table EV5

Table EV6

Table EV7

Table EV8

Table EV9

Table EV10

Table EV11

## Data Availability

The datasets and computer code produced in this study are available in the following databases: Modeling computer scripts: GitHub (https://github.com/ptjouhten/EvolveX). Whole genome sequencing data: European Nucleotide Archive (ENA: https://www.ebi.ac.uk/ena/browser/home) study PRJEB40761 with accession numbers ERS5457098 and ERS5290477–ERS5290502 for the parental and evolved samples and in study PRJEB41108 with accession numbers ERS5293678–ERS5293725 for the Panel of Normals (PoN)‐samples. GC–MS data: Metabolights database MTBLS2208 https://www.ebi.ac.uk/metabolights/
RNA‐sequencing data: ArrayExpress database E‐MTAB‐10019 https://www.ebi.ac.uk/arrayexpress/
Proteomics data: ProteomeXchange Consortium via the PRIDE (Perez‐Riverol *et al*, 2019) partner repository PXD023171 https://www.ebi.ac.uk/pride/
