# Author Correction: Predictive evolution of metabolic phenotypes using model-designed environments

**DOI:** 10.1038/s44320-024-00066-1

**Published:** 2024-09-25

**Authors:** Paula Jouhten, Dimitrios Konstantinidis, Filipa Pereira, Sergej Andrejev, Kristina Grkovska, Sandra Castillo, Payam Ghiaci, Gemma Beltran, Eivind Almaas, Albert Mas, Jonas Warringer, Ramon Gonzalez, Pilar Morales, Kiran R Patil

**Affiliations:** 1 European Molecular Biology Laboratory, Heidelberg, 69117 Germany; 2 VTT Technical Research Centre of Finland Ltd, Espoo, 02044 VTT Finland; 3 Department of Bioproducts and Biosystems, Aalto University, Espoo, 00076 Aalto Finland; 4 Department of Chemistry and Molecular Biology, University of Gothenburg, Gothenburg, 40530 Sweden; 5 Universitat Rovira i Virgili, Dept Bioquímica i Biotecnologia, Facultat d’Enologia, 43007 Tarragona, Spain; 6 Department of Biotechnology and Food Science, NTNU - Norwegian University of Science and Technology, Trondheim, Norway; 7 Instituto de Ciencias de la Vid y delVino (CSIC, Gobierno de la Rioja, Universidad de La Rioja), Finca La Grajera, Carretera de Burgos, km 6, 26071 Logroño, Spain; 8 Medical Research Council (MRC) Toxicology Unit, University of Cambridge, Cambridge, CB2 1QR UK

## Abstract

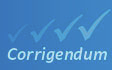

**Correction to:** *Molecular Systems Biology* (2022) 18:e10980. 10.15252/msb.202210980 | Published online 6 October 2022

**The 7th author’s name is corrected**.

The 7th author’s name is corrected from: Payam Ghiachi

To: (Changes in bold). Payam **Ghiaci**

