# Supplementary material for: Predictive evolution of metabolic phenotypes using model‐designed environments
Source: Mol Syst Biol. 2022 Oct 6;18(10):e10980. doi: 10.15252/msb.202210980 (PMC9536503; doi:10.15252/msb.202210980)
Supplement: Supplementary file 12 [file MSB-18-e10980-s004.pdf]

molecular systems biology

Predictive evolution of metabolic phenotypes using model-designed environments

Paula Jouhten, Dimitrios Konstantinidis, Filipa Pereira, Sergej Andrejev, Kristina Grkovska, Sandra Castillo, Payam Ghiaci, Gemma Beltran, Eivind Almaas, Albert Mas, Jonas Warringer, Ramon Gonzalez, Pilar Morales, and Kiran Patil
DOI: 10.15252/msb.202210980

Corresponding author(s): Kiran Patil (kp533@cam.ac.uk)

Review Timeline:

Submission Date:	16th Feb 22
Editorial Decision:	18th Mar 22
Revision Received:	29th Jun 22
Editorial Decision:	19th Aug 22
Revision Received:	24th Aug 22
Accepted:	26th Aug 22

Editor: Maria Polychronidou

Transaction Report:

(Note: With the exception of the correction of typographical or spelling errors that could be a source of ambiguity, letters and reports are not edited. Depending on transfer agreements, referee reports obtained elsewhere may or may not be included in this compilation. Referee reports are anonymous unless the Referee chooses to sign their reports.)

RE: MSB-2022-10980, Predictive evolution of metabolic phenotypes using model-designed selection environments

Thank you again for submitting your work to Molecular Systems Biology. We have now heard back from the three referees who agreed to evaluate your study. All three reviewers have positive remarks about the relevance of the presented approach for biotechnology. They raise however a series of concerns, which we would ask you to address in a revision.

I think that the recommendations of the reviewers are clear, and I therefore see no need to repeat any of the comments listed below. All issues raised need to be satisfactorily addressed. Please contact me in case you would like to discuss any of the issues raised.

On a more editorial level, we would ask you to address the following points:

Reviewer #1:

Summary

The authors present a novel method (EvolveX) to address the difficulties in adaptive evolution for increased secondary metabolite production that trades off with growth rate. EvolveX first predicts which fluxes are required for the production of the secondary metabolites within the application environment. Then selection environments are predicted which couple growth to a subset of the fluxes required for the secondary metabolite production. All evolution environments are scored and some were picked for an experimental test for adaptive evolution of secondary metabolites. Thereafter the strains were tested in micro vinification experiments for increased secondary metabolite production. At last, both proteomics and transcriptomics were measured within micro vinification experiments to link the evolved phenotype to the model predictions.

General remarks

In general the ideas presented are novel and very interesting and potentially very relevant for strain improvement through evolutionary engineering. The authors do show some evidence that supports their findings, but they should improve transparancy of the use and limitations of the approach by a more comprehensive analysis of the data. The paper now appears to be biased towards the successes they achieved, and seem to miss some key descriptions, analysis and results for a full assessment. In particular, the claim that the algorithm is truly predictive required more detailed analysis and is not yet fully convincing to this reviewer.

Major points

1. The explanation of the model used in sections "(Predicting) Evolution environment" is not very comprehensible. I find the underpinning with basic evolution theory distracting and not very useful, as it at most inspired the actual algorithm, which could be explained purely in metabolic network terms. E.g. it seems that R in eq 3 and eq 4 have different units (but they are not given). In addition, that algorithm is not very easily understood from the text. It could help tremendously if the main ideas of the method were illustrated with a toy model.

2. The authors claim that the metabolites predicted by the method are indeed increased. However, they do not show the complete analysis of all GC-MS analyzed secondary metabolites. Therefore, the analysis on whether they could actually use a specific environment to evolve for specific increased secondary metabolite production is incomplete. It is unclear if the increase in volatiles is specifically geared towards the predictions, or just aspecific changes picked from a larger list of changes that are the natural result of evolving under different conditions - especially if you add amino acids such flavors can be expected. The fact that no specific mutations could be found on glycerol, but rather full genome duplications also points to lack of specificity. In this respect I also miss some negative controls that indeed only in the specific (model-derived) selective environments are these compounds selected for. The authors could for example have strengthen their claim about non-intuitive evolution environments (page 9, sentence 32) by performing control evolutions with solely ethanol or glycerol in combination with ammonia, or with some other amino acid that would not lead to flavor according to the model.

The fact that isoamyl acetate production is not increased for the ethanol-evolved strain but is for the glycerol-evolved strain feeds the uncertainty of being able to predict specific increased secondary metabolite production. This discrepancy is not openly discussed in the main text.

3. Expression analysis was done in the application condition to test some phenotypic effects and find molecular mechanisms. However, comprehensive analysis of the proteomics and transcriptomics data is not shown, rather some highlights (successes) are discussed. I lack a statistical underpinning of the assessment that the changes were more specific to the flux bases that to any other gene set. So I am not convinced they can claim that (page 9 line 18): "Overall, the protein abundance changes in evolved cells were centered on the aroma synthesis pathways in accord with the model predictions."

4. In the methods, some details are missing or not made explicit. For example, in the characterization of evolved strains (page 24; sentence 11) the authors speak of the best growing strain without implying what this is or showing the growth improvement of the best growing strain. The authors state that at the end of the evolution experiment they picked "isolates performing the best" (page 24 sentence 1), without showing or explaining what this is exactly (e.g. increased growth rate). This is relevant information, as the number of generations used is relatively small compared to other studies where 300-500 generations is typically used.

Additionally, according to the Materials and Methods the conditions differ between the secondary compound cultivations and the small-scale fermentations for transcriptomics and proteomics, which is not discussed. These conditions may affect physiology.

Minor points
Figure 3 (a,b) contains a lot of clones annotated in a similar fashion (e.g. E1, G1, etc.). It is not made clear what the exact distinction is between these clones and if more data is available for them.
Page 6 line 9: Many of the aroma compounds mentioned contain only carbon. I understand they are often derived from amino acids but not all readers of MSB will understand this. Please explain.
Page 7, line 20 onwards: since you measured both populations and isolates, please make sure that in the paragraph that follows it is always clear which genetic variation belongs to which sample.
Page 9, sentence 22 "... rooted in the laws of thermodynamics..." . It is not clear where that comes from and is confusing.
Within the discussion (page 10 sentence 8), it is claimed "the need to know" is circumvented. Yet the authors use a well-curated genome-scale metabolic model and well-studied pathways. They could be more subtle and try to explain these potential hurdles in their discussion. They also came across the limitation of strain to strain differences in selection of wine strains for their experiments, which is mentioned in the materials and methods. In addition (page 10 sentence 15), the authors claim that their method could help to understand complex adaptive processed, yet mechanistic understanding of their own study is minimal.

Reviewer #2:

The authors present and validate a novel computational approach (EvolveX) to design laboratory evolution experiments to select microbial strains with enhanced metabolite secretion phenotypes. The idea of the method is to circumvent the trade-off between growth and secretion by adapting to an environment where growth becomes correlated with traits that underlie the desired secretion phenotypes. For this, they utilize the constrained-based framework of genome-scale metabolic modelling to predict specific environments where laboratory evolution should be carried out. By focusing on two groups of aroma compounds, they experimentally validate the method in budding yeast.

The work goes beyond the state of the art and is an important step towards employing model-based evolutionary predictions in biotechnology. More broadly, the work also has relevance to our understanding of trait-trait correlations and their environmental dependence. Thus, the work has a high potential to be of broad interest and utility.

However, I have a coupled of concerns surrounding the experimental validation of the method.

Major points:

1) The step-by-step description of EvolveX in the main text and the justification of each step are not crystal clear. For example, how is flux coupling, as defined by Maranas et al. (Burgard et al. 2004), actually used? It is often mentioned, but its usage was not clear to me from the 4 steps described. I first thought it was used to define the flux basis, but the Method section suggests otherwise.
Also, what does 'response to selection' mean in the computational model? Is it presence of flux coupling or extent of flux correlation? And what's exactly the justification of Eq 4? Why should higher flux per unit of growth correspond to higher relative (proportional?) response to selection? Isn't the covariance what matters for selection response?

2) To experimentally demonstrate that specific aroma compounds are secreted at increased rates after selection in the predicted environments, the authors compare the evolved lines to their ancestors and to each other. However, I couldn't find any negative controls for the evolutionary experiments. It might be possible that adaptation to various different nutrient environments enhances aroma compound production as pleiotropic effects and so the observed secretion phenotypes are not specific. While I wouldn't request to carry out new lab evolution, I encourage the authors to measure, if possible, the aroma production of strains that were evolved in the laboratory in similar studies but for other goals (i.e. better growth on glucose, increased heat tolerance, etc.). Alternatively, it would be informative to comparing the compound productions with those of other wine strains. Such comparisons could support the uniqueness of the aroma production profile achieved by lab evolution.

3) How similar are the aroma production profiles of the independently evolved parallel lines that were exposed to the same selection pressure? Are they more similar within evolution environment than lines evolved in different environments? I suggest to show all parallel lines on the PCA plot (Fig 2b).
Similar question applies to similarities in transcriptome and proteome profiles. Do the two selection regimes result in distinct omics changes that are repeatedly observed among parallel evolved lines? One would expect clear physiological changes that are specific to the selection regime.
Btw, I suggest to report full transcriptome and proteome profiles (i.e. including genes showing no differential expression) as Supplementary Tables.

4) I wonder whether the amount of increase in aroma compound production is biotechnologically relevant. Even if the obtained increase is relatively modest, it would be important to reflect upon this point.
Btw, can the AU units be interpreted as relative concentrations? If so, it would be useful to show them as relative values to parental strain. Also, are these values normalized to biomass?

5) It is unclear from reading the text how much fitness gain occurred in the evolution environment and how much fitness cost it incurred in the application environment. These would be important to show. Also, would a decreased growth rate or yield in the application environment not cancel any gain in the production of the volatile compounds? This should be clarified.

6) I couldn't find a systematic comparison of predicted flux-rerouting to measured omics changes in the evolved lines. The data shown on Fig 3d-e are convincing, but a statistical analysis would be needed to support the claim that abundance changes were centered on the aroma synthesis pathways. Transcript and proteome changes that occur in multiple parallel evolved lines would

be of special relevance as they likely reflect changes that are essential for the phenotype.

7) EvolveX is indeed novel but the authors haven't discussed alternative approaches, such as making metabolite production growth coupled, e.g. Kamp & Klamt Nat Comm 2016, or performing adaptive laboratory evolution on strains in which specific production fluxes have been growth coupled first (https://doi.org/10.1002/bit.21694).
It would be important to discuss how EvolveX compares to such methods in applicability. Besides, growth-coupling methods could potentially increase the scope of EvolveX by making the tacking traits growth coupled in a desired evolution environment.

Minor points:

page 6 , line ~ 15 Please cite some literature supporting that these particular aroma compound productions are desired traits in wine yeasts.

page 6 , line ~ 23 "assessed for literature evidence of feasibility of S. cerevisiae growth" Could you please specify how many of the top solutions seemed to be clearly unfeasible, this could give some insight how usable this approach generally is.

page 22 , line ~ 23-27. Could you please specify which strains were tested and used in the experiments? Were strain-specific models used (i.e. from the Nielsen lab)?

Table S3 needs more explanation. What are the units in the top table? What is weight loss on the plot? Ideally, supplementary tables and figures should be shown separately and with captions containing enough details to be understandable by the general reader.

Reviewer #3:

Whereas particular phenotypes such as (e.g.) oxidative stress tolerance can be readily evolved into an organism by using the appropriate evolution environment, it is difficult to use adaptive evolution to produce positive selection of phenotypes that trade-off with key fitness metric such as cell growth. Although artificial selection of maladaptive phenotypes is possible, this is usually done through combinatorial mutagenesis and is usually limited to single proteins due to the exponential demands of combinatorial programs. For complex maladaptive traits such as secretion of wine musk - sugar-rich compounds that the organism would rather eat than secrete! - such experiments are prohibited by the complexity and uncertainty of the underlying genotype.

Here, Patil and coworkers demonstrate how it is possible to positively select for otherwise neutral or maladaptive complex phenotypes by selecting for a 'tacking trait'. Here, the 'tacking trait' is an altered metabolic flux resulting from a modified growth media (the "evolution condition"). To eat the modified growth media more quickly, the organism (through natural mutation and selection over the course of several hundred cell passages within the laboratory) will gradually rewire its metabolic flux to process the modified nutrients more quickly. Hence, the 'tacking trait' is coupled to fitness (growth rate) and is therefore positively selected. When this evolved organism is then placed within a base growth media (the "application condition"), the altered metabolic flux is not preferable for cell growth but is preferable for the production of target compound(s) (in this case, wine musk). This process is suitably demonstrated in Figure 1. To predict the altered media required to produce the desirable 'tacking trait' within the 'application media', the authors devised an algorithm, called EvolveX, which identifies a tacking trait for the desirable phenotype and the set of nutrients that would be required to adaptively evolve that tacking trait.

The application of 'tacking trait'-based evolution was demonstrated in its entirety by producing a strain of S. cerevisiae with enhanced musk secretion. Although these results would have made a viable manuscript on their own standing, the authors went above and beyond by documenting the precise mutations that occurred and how these mutations resulted in the formation of both the 'tacking trait' and the desirable 'musk phenotype'. In my opinion, this manuscript is a beautiful synthesis of core evolutionary theory, computational and metabolic modelling, genomics, transcriptomics, and proteomics. Furthermore, the secretion of virtually any compound (unless a waste product) is maladaptive to the host organism, and is a considerable and persistent problem in industrial microbiology and metabolic engineering. The authors appear to be mobilizing their EvolveX program into a patentable and distributable platform. Therefore, the relevance of the manuscript is quite broad. Although prolonged exposure in the 'application condition' may result in adaptive evolution back to the original metabolic flux, the authors have emphasized that industrial scale-up is feasible in batch productions by maintaining a seed culture within the 'evolution environment'.

I am compelled to recommend 'Accept As Is' and offer my congratulations to the authors for this excellent manuscript!

I do have four comments. I will leave it to the authors' discretion whether they wish to incorporate these comments into the manuscript. This can be handled during the pre-print/proofing stage and should not delay publication:

1. For the Introduction, it could be hazardous to omit mentioning alternative works in the literature that also explore adaptive laboratory evolution for selecting maladaptive traits using metabolic modelling. For example, it's fairly common to apply adaptive evolution to a product-producing pathway by knocking out genes in the host to growth-couple product formation. These knockouts can be model-driven, from a metabolic model, somewhat similar to what was done in this manuscript. For example, I believe this functionality is now built directly into cobrapy, the most common flux balance analysis software.
2. I'm curious how the wine produced by your evolved yeast would actually taste. Yes, aroma molecules are enhanced, but it's also possible that the wine produced by these yeast either tastes or smells awful due to unanticipated negative consequences associated with changing the normal metabolic profile of yeast. Or, perhaps this will be the most delicious wine the world has ever seen! Are you following-up this work with a live application in a winery with qualitative taste tests?
3. For a future manuscript, consider incorporating a value function into EvolveX such that the goal is to minimize changes to metabolites other than the metabolites of interest. This could be useful if unintended negative consequences to yeast taste/smell are indeed observed due to off-target changes within the broader metabolome (per comment #3).
4. Typo; Page 6 line 25 should read "An evolutionary environment"

We are grateful to all reviewers for their positive comments and constructive suggestions. Please, find below our point-by-point response to all comments.

- Reviewer comments in *blue italics (font size 9)*
- Our response in black (font size 11)
- Revised manuscript texts reproduced here in black (font size 9)

Reviewer #1:

Summary

The authors present a novel method (EvolveX) to address the difficulties in adaptive evolution for increased secondary metabolite production that trades off with growth rate. EvolveX first predicts which fluxes are required for the production of the secondary metabolites within the application environment. Then selection environments are predicted which couple growth to a subset of the fluxes required for the secondary metabolite production. All evolution environments are scored and some were picked for an experimental test for adaptive evolution of secondary metabolites. Thereafter the strains were tested in micro vinification experiments for increased secondary metabolite production. At last, both proteomics and transcriptomics were measured within micro vinification experiments to link the evolved phenotype to the model predictions.

General remarks

In general the ideas presented are novel and very interesting and potentially very relevant for strain improvement through evolutionary engineering. The authors do show some evidence that supports their findings, but they should improve transparancy of the use and limitations of the approach by a more comprehensive analysis of the data. The paper now appears to be biased towards the successes they achieved, and seem to miss some key descriptions, analysis and results for a full assessment. In particular, the claim that the algorithm is truly predictive required more detailed analysis and is not yet fully convincing to this reviewer.

Major points

1. The explanation of the model used in sections "(Predicting) Evolution environment" is not very comprehensible. I find the underpinning with basic evolution theory distracting and not very useful, as it at most inspired the actual algorithm, which could be explained purely in metabolic network terms. E.g. it seems that R in eq 3 and eq 4 have different units (but they are not given). In addition, that algorithm is not very easily understood from the text. It could help tremendously if the main ideas of the method were illustrated with a toy model.

We thank the Reviewer for indicating this unclarity in our method description. We have improved the clarity by moving the basic evolution theory to a separate Box (Box 1, reproduced below). The Box text now also clarifies the difference between the units in Equations 3 and 4. Equation 3 describes the dependence of response to selection on the covariances between traits with unit of ‘trait unit multiplied with fitness unit’, whereas Equation 4 describes dimensionless flux coupling between a trait and the fitness. Covariances between metabolic fluxes follow from flux coupling and this we have now included in the text as a clarification with additional references (Box 1, please see below). We have also further improved the illustration of the method with the small toy model in Figure 1E-G by revising the description to indicate flux coupling when appropriate (Figure 1 legend, reproduced below).

Box1. Trait-fitness dependences predictable as flux couplings

The selection acting on a phenotypic trait is the covariance between the trait and the relative fitness, as described by Robertson-Price identity (Price, 1970; Rausher, 1992; Robertson, 1966, 1968) (Equation 1).

$$s = cov(w, z) \qquad \text{Equation 1}$$

where s is the selection differential, w fitness, and z the trait of interest.

When there is genetic covariance between the trait and relative fitness, evolutionary response to selection can occur (Equation 2, the secondary theorem of selection).

$$R = s_g = cov_a(w, z) \qquad \text{Equation 2}$$

where R is the response to selection with units of the trait and fitness multiplied, sg is the genetic selection differential, and $cov_a(w,z)$ is the additive genetic covariance.

Equation 2 generalizes to a multivariate form for multiple traits (Rausher, 1992).

$$\boldsymbol{R} = cov_a(w, \boldsymbol{z}) \qquad \text{Equation3}$$

We now consider the case of metabolic traits, which can be represented and modelled as a set of metabolic fluxes (net reaction rates). Metabolic trait interdependencies under a given chemical environment can then be predicted using genome-scale metabolic models as flux couplings (Burgard *et al*, 2004). Two metabolic reactions are coupled if a non-zero flux through one reaction implies a non-zero flux through the other. Flux covariance follows from flux coupling (Heinonen *et al*, 2019; Pradhan, 2019; Thommes *et al*, 2019). Importantly for modelling evolutionary adaptation, flux coupling implies genetic dependences between the corresponding enzyme-coding genes (Notebaart *et al*, 2008).

To predict relative responses of a metabolic trait to selection, we use its coupling to the specific growth rate (proxy for mean fitness). Analogous to the secondary theorem of selection (Equation 3), this gives:

$$\boldsymbol{F}_v = \frac{\boldsymbol{v}}{\mu} \qquad \text{Equation 4}$$

where $\mathbf{F}_v$ is the relative unitless responses of single-flux metabolic traits to selection, v the metabolic fluxes, and μ the specific growth rate. Thus, higher the flux per growth unit, stronger the selection.

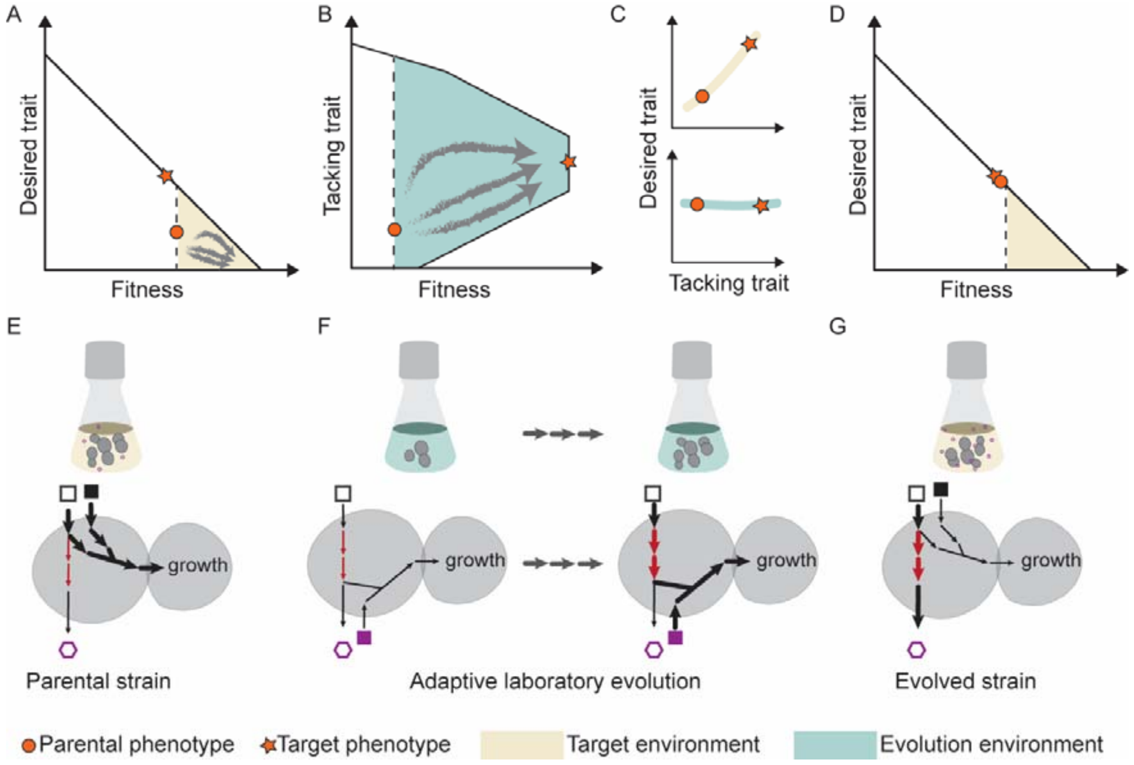

Figure 1. Darwinian selection in the absence of fitness advantage through an evolution environment and a tacking trait. Current phenotype is represented with an orange circle whereas the orange star represents the desired target phenotype. A) In the application environment (yellow), Darwinian selection (grey arrows) enriches cells with fitter phenotypes but with diminished desired trait. B) The tacking trait is chosen to be coupled with fitness in the evolution environment and can therefore be improved through Darwinian selection. C) The tacking trait is also characterized by direct coupling to the desired target trait in the application environment, even though not so in the evolution environment (green). D) Evolved cells with a strengthened tacking trait (through selection in the evolution environment) manifest an improved desired trait in the application environment. E-G) A simple metabolic network illustrating the evolution environment and the tacking trait. The desired trait is the production flux of a compound (open hexagon). The squares depict available nutrients, which differ between the target and evolution environments. The arrows represent metabolic fluxes, the thicker the arrow the higher the flux. The tacking trait (red arrows), which is part of the flux basis of the desired trait, is flux coupled to cell growth flux (i.e. proxy of mean fitness) in the evolution environment. Thus, the tacking trait can be improved through adaptive evolution in the evolution environment. Due to the flux coupling in the application environment, the improved tacking trait leads to the enhanced desired target trait (i.e. increased target compound secretion).

2. The authors claim that the metabolites predicted by the method are indeed increased. However, they do not show the complete analysis of all GC-MS analyzed secondary metabolites. Therefore, the analysis on whether they could actually use a specific environment to evolve for specific increased secondary metabolite production is incomplete. It is unclear if the increase in volatiles is specifically geared towards the predictions, or just aspecific changes picked from a larger list of changes that are the natural result of evolving under different conditions - especially if you add amino acids such flavors can be expected. The fact that no specific mutations could be found on glycerol, but rather full genome duplications also points to lack of specificity. In this respect I also miss some negative controls that indeed only in the specific (model-derived) selective environments are these compounds selected for. The authors could for example have strengthen their claim about non-intuitive evolution environments (page 9, sentence 32) by performing control evolutions with solely ethanol or glycerol in combination with ammonia, or with some other amino acid that would not lead to flavor according to the model.

We have now included the data on all the 28 volatile compounds, quantified in cultures grown on natural grape must, in the supplementary material and included a visualization of the PCA in Figure 2F (reproduced below, also accordingly revised Results section reproduced below). When all 28 compounds are considered, the parental and evolved strains do not cluster separately (Figure 2F); however, when considering the target compounds (Figure 2G), the variance leads to expected clustering driven by the respective compound profiles. This supports a degree of selectivity in the aroma changes. The changes

observed in other, non-target, compounds, are small with the evolved lineages being in the range of parental variation.

We observed changes shared between the isolates selected in the two different evolution environments. This is expected since the tacking traits of the two sets of target aroma were partially overlapping for the aromatic amino acids and branched chain amino acids derived target aromas, by 2 fluxes out of 7 and 11, respectively. We have clarified this in the revised manuscript (revised text reproduced below). We also note that in this study we did not directly optimize the evolution environments for specificity of the desired compound generation (but summed tacking trait flux couplings with growth). Thus, changes in target compounds as well as in other compounds is consistent with the design. We have clarified this in the revised manuscript text (please see the revised paragraph below).

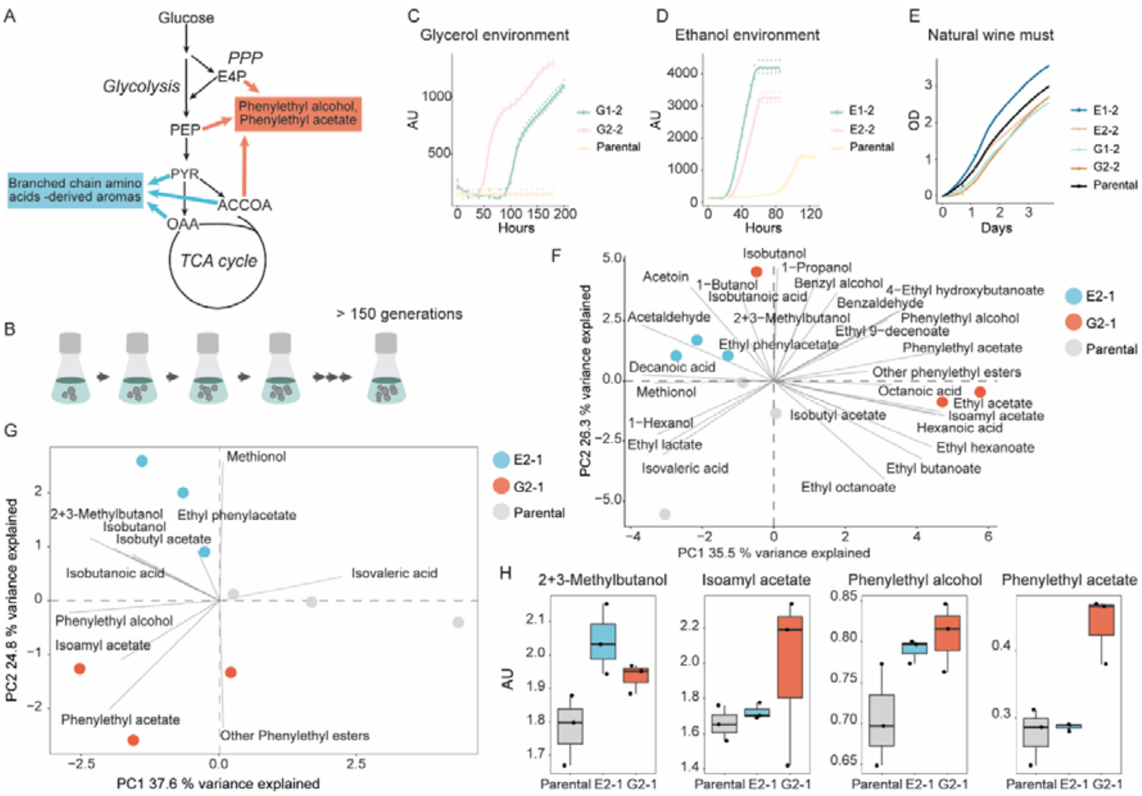

Figure 2. Aroma production changes detected in evolved yeast strains. A) Origin of aroma compounds in the yeast central metabolism: branched-chain amino acid derived compounds (esp. 2-methyl-1-butanol, 3-methyl-1-butanol, isoamyl acetate and 2-methylbutylacetate), and aromatic amino acid derived compounds (esp. phenylethyl alcohol and phenylethyl acetate). Acetate esters of higher alcohols share an acetyl-CoA (ACCOA) precursor. B) Parental wine strain of *S. cerevisiae* was adaptively evolved in both ethanol environment and glycerol environment for over 150 generations. C) Evolved single colony isolates had improved growth in glycerol environment compared to parental. The growth of isolates G1-2 and G2-2 and the parental characterized in three biological replicates as backscattered light (AU – arbitrary units). D) Evolved single colony isolates had improved growth in ethanol environment compared to parental. The growth of isolates E1-2 and E2-2 and the parental characterized in three biological replicates as backscattered light (AU – arbitrary units). E) Evolved single colony isolates maintained similar to parental growth ability characterized in single biological replicates as carbon loss in natural wine must fermentations. F) Principal components analysis of quantified 28 volatile aroma compounds in natural wine must fermentations, with the parental (grey) and evolved strains in three biological replicates. Evolved strain from the ethanol evolution environment (ethanol, arginine, glycine), E2-1, in light blue, and that from the glycerol evolution environment (glycerol, phenylalanine, threonine), G2-1, in orange. G) Principal components analysis of aromatic and branched amino acids-derived volatile compound profiles of natural wine must fermentations, with the parental (grey) and evolved strains (E2-1 in light blue, G2-1 in orange) in three biological replicates. H) Changes in selected aroma compound abundances in wine must fermentations. AU –

arbitrary units. E2-1 (light blue) was selected in the ethanol environment, and G2-1 (orange) was selected in the glycerol environment. 2+3-methylbutanol (a combined pool of 2-methyl-1-butanol and 3-methyl-1-butanol) and isoamyl acetate (acetate ester of 3-methyl-1-butanol) were the desired target aromas of the ethanol environment, deriving from branched-chain amino acids. Phenylethyl alcohol and its acetate ester, phenylethyl acetate, were the desired target aromas of the glycerol environment.

Revised text:
Mass-spectrometry analysis of the volatile compounds (28 quantified, supplementary information, Table S6) in wine must fermentations with the parental strain and evolved isolates provided a view on the changes in volatiles following evolution. In principal components analysis, the strains did not cluster by their history (Figure 2F), supporting that the volatile metabolite production was not universally impacted following laboratory evolution However, the principal components analysis considering only the target compounds, the aroma profiles clustered by the evolution environment and separately from the parental (Figure 2G). The first principal component (PC1, 37.6 % of total variance) distinguished parental from the evolved strains. In accordance with the model, this separation is driven by the overlap of the two tacking traits (transketolase and ribulose 5-phosphate 3-epimerase fluxes; supplementary information, Table S2). Further attesting the model, the isolates selected in the ethanol and glycerol evolution environments were separated mainly by the target aroma compounds (PC2, 24.8 % of total variance, Figure 2G-H). While for target aroma compound isoamyl acetate we could not validate the model predictions (i.e. level similar to parental in fermentations with E2-1), phenylethylacetate was specifically increased in the wine must fermentations with the isolates selected in the glycerol environment (Figure 2H). Similarly, the combined pool of branched-chain amino acid-derived aroma compounds 2-methyl-1-butanol and 3-methyl-1-butanol was increased only for the isolate selected in the ethanol environment. Together, evolved isolates featured increased aroma formation in wine must according to the EvolveX predictions.

We did not observe full genome duplications but aneuploidy in some chromosomes. We have now clarified this in the revised manuscript (text copied below). The aneuploidy could be counteracted at higher level regulation (e.g., protein degradation or other mechanisms) (Muenzner et al., 2022).

Revised text:
The clones and the populations selected in the glycerol environment had only few SNVs, and no genes showed recurrent SNVs. However, CNVs were prevalent, with multiple triplicated segments observed in several cases (Figure 3B; supplementary information, Table S4). This extensive variation meant that no particular genes or pathways could be directly linked with either growth or aroma production. Indeed, many of the duplicated genes could be dosage compensated (Muenzner et al, 2022). Therefore, we next resorted to analyzing changes at the transcriptomic and proteomic levels. The evolved cells were characterized both in the application environment (wine must, same batch as was used for determining the aroma profiles) and in their corresponding evolution environments (supplementary information, Table S6, Table S7). In all cases, the overlap between transcript-level and protein-level changes was below 6 %, indicating major role of post-transcriptional regulation in both the improved aroma generation in wine must and in the improved fitness in the evolution environments.

The phenotyping was performed in the natural grape must without any supplementation. The text is changed to clarify this (revised paragraph below).

Revised text:
In each of the two selected environments, three replicate populations of a diploid wine yeast strain were independently evolved asexually for over 150 generations (Figure 2B). Growth improvement was observed in both evolution environments (Figure 2C-D, supplementary information, Table S4). In the selected isolates evolved in ethanol environment an increase in maximum specific growth rate of over two-fold was estimated (Figure 2D, supplementary information, Table S4). Aroma production and growth physiology of single colony isolates were assessed in natural wine must fermentations (without any aroma precursor supplementation). All evolved isolates maintained their fermentation performance in the natural wine must (Figure 2E, supplementary information, Table S5), indicating their suitability for use in wine fermentations.

The two selected evolution environments have distinct sets of fluxes coupled to growth. Thus, they act as controls for each other for differential selection on fluxes. From a metabolic network perspective, they are as good controls as glycerol + ammonium and ethanol + ammonium. We have now clarified this in the revised manuscript text (below).

Revised text:
To identify a suitable evolution environment for enhancing the target aroma generation and corresponding tacking traits, we assessed all 1540 combinations of up to three carbon and nitrogen sources, chosen from 22 common constituents of yeast growth media. All combinations were ranked for their suitability for positively selecting the flux bases of the target aroma generation (via the tacking traits) using the EvolveX score

(supplementary information, Table S1). High-scoring environments were assessed for literature evidence of feasibility of *S. cerevisiae* growth. Two of the high-scoring environments, which were among the top 20 of 1171 growth-supporting solutions, were selected for experimental validation. Evolution environment containing glycerol, phenylalanine, and threonine as sole carbon and nitrogen sources was chosen for phenylethyl alcohol and phenylethylacetate production. In this environment, hereafter called glycerol environment (Figure 2A), 7 fluxes (out of 20 in the flux basis) formed the tacking trait of phenylethyl alcohol and phenylethylacetate production (supplementary information, Table S2). For branched-chain amino acid-derived aromas, ethanol environment (Figure 2A), containing ethanol, arginine, and glycine, was selected for experimental validation. In the ethanol environment 11 fluxes (out of 44 in the flux basis) formed the tacking trait (supplementary information, Table S2). The two tacking traits included two common fluxes (transketolase 1, ribulose 5-phosphate epimerase). However, only eight common fluxes were predicted to be positively selected in the two evolution environments while 57 fluxes were predicted to be selected only in one of the two evolution environments (supplementary information, Table S3). Notably, the glycerol environment and in the ethanol environment were predicted to expose positive selection on 17 (out of 29) and 20 (out of 44) common fluxes with intuitive control environments glycerol and ammonium and ethanol and ammonium, respectively (supplementary information, Table S3). Thus, the EvolveX designed glycerol and ethanol evolution environments act as appropriate controls to each other.

The fact that isoamyl acetate production is not increased for the ethanol-evolved strain but is for the glycerol-evolved strain feeds the uncertainty of being able to predict specific increased secondary metabolite production. This discrepancy is not openly discussed in the main text.

We thank the reviewer for pointing out the limitation of our discussion on the overlapping tacking traits. Indeed, that tacking traits of the two desired target compound sets were overlapping and therefore such phenotypic effects are to be expected. However, isoamyl acetate varied a lot between the biological replicate fermentations performed with the isolate evolved in glycerol environment (Figure 2H). Therefore, we could not for that compound (one in the group) validate the model prediction for ethanol environment. We have expanded the discussion on this in the revised manuscript text.

Revised text:
Mass-spectrometry analysis of the volatile compounds (28 quantified, supplementary information, Table S6) in wine must fermentations with the parental strain and evolved isolates provided a view on the changes in volatiles following evolution. In principal components analysis, the strains did not cluster by their history (Figure 2F), supporting that the volatile metabolite production was not universally impacted following laboratory evolution However, the principal components analysis considering only the target compounds, the aroma profiles clustered by the evolution environment and separately from the parental (Figure 2G). The first principal component (PC1, 37.6 % of total variance) distinguished parental from the evolved strains. In accordance with the model, this separation is driven by the overlap of the two tacking traits (transketolase and ribulose 5-phosphate 3-epimerase fluxes; supplementary information, Table S2). Further attesting the model, the isolates selected in the ethanol and glycerol evolution environments were separated mainly by the target aroma compounds (PC2, 24.8 % of total variance, Figure 2G-H). While for target aroma compound isoamyl acetate we could not validate the model predictions (i.e. level similar to parental in fermentations with E2-1), phenylethylacetate was specifically increased in the wine must fermentations with the isolates selected in the glycerol environment (Figure 2H). Similarly, the combined pool of branched-chain amino acid-derived aroma compounds 2-methyl-1-butanol and 3-methyl-1-butanol was increased only for the isolate selected in the ethanol environment. Together, evolved isolates featured increased aroma formation in wine must according to the EvolveX predictions.

3. Expression analysis was done in the application condition to test some phenotypic effects and find molecular mechanisms. However, comprehensive analysis of the proteomics and transcriptomics data is not shown, rather some highlights (successes) are discussed. I lack a statistical underpinning of the assessment that the changes were more specific to the flux bases that to any other gene set. So I am not convinced they can claim that (page 9 line 18): "Overall, the protein abundance changes in evolved cells were centered on the aroma synthesis pathways in accord with the model predictions."

We apologize for the insufficient description of the transcriptomics and proteomics results. We have clarified in the revised manuscript text that the predictions (flux bases, tacking traits, and selection) are performed for fluxes and not gene expression (please see the revised text below). Since the genetic underpinnings of the fluxes are more complex than just the expression of the genes encoding the enzymes that catalyze the corresponding reactions (and are sometimes unknown), changes in gene expression / protein levels are not in all cases necessary for flux change (the control could be, e.g., at substrate/co-factor availability level or at PTM level etc.). This point argues for the strength of the developed

method, viz., targeting the selection without knowing the genetic underpinnings of the desired traits.

Nevertheless, we used hypergeometric test to assess the overlap between proteins significantly higher in abundance and those predicted by the model to be positively selected. Significant overlaps were found; though the numbers of differentially abundant proteins, and metabolic enzymes, in the evolved strains were low. We have revised the manuscript to visualize comprehensively the numbers of differentially abundant proteins in evolved strains, and to indicate significant overlaps to support the statements on relevance of the model predictions (please, see the revised text below). Further, all RNA-seq and proteomics data is supplemented with the revised manuscript.

Revised text:
To search for a suitable evolution environment, i.e., a defined chemical environment in which the adaptive evolution is to take place, we use the basis provided by the selection response relation (Equations 3, 4). Ideally, the evolution environment would be chosen such that there is a direct selection for the desired trait through flux coupling with the cell growth. This, however, will only rarely be possible as most desired traits, such as metabolite secretion, are at a trade-off with cell growth due to competition for metabolic precursors and co-factors (Jouhten *et al*, 2016; Nielsen & Keasling, 2016) (Figure 1A). We therefore aim at growth coupling of a secondary trait, which we term tacking trait. Tacking trait is here defined as a set of fluxes that are flux coupled (Burgard *et al.*, 2004) to cell growth in the evolution environment, and with the desired trait in the application environment (Figure 1B). We note that it is neither necessary for the tacking trait to be coupled with the desired trait in the evolution environment, nor it is likely due to the trade-off with growth. Further, the tacking trait is necessarily a proper subset of fluxes that must increase or decrease for the desired trait enhancement in the application environment. Due to the environment-dependence of genetic correlations between traits (Equation 3), the tacking trait and the evolution environment are intrinsically linked and need to be identified simultaneously.

Revised text:
We targeted two main groups of aroma compounds: i) phenylethyl alcohol and its acetate ester, phenylethylacetate, which have a rose and honey scent and raspberry-like flavor; and ii) branched-chain amino acid-derived higher alcohols (2-methyl-1-butanol and 3-methyl-1-butanol) and their acetate esters (2-methylbutylacetate and isoamyl acetate) (Carpena *et al*, 2021; Swiegers *et al*, 2005), which have a banana and pear scent and fruity flavor. All these aroma compounds derive from amino acids' (L-phenylalanine and branched chain amino acids) carbon backbones and contain no nitrogen. The flux bases of the target aroma syntheses were defined as a minimum set of fluxes that have to increase for the particular target aroma generation to be enhanced. Similarly, flux bases could include fluxes that should be negatively selected for desired trait development.

Revised text:
To identify a suitable evolution environment for enhancing the target aroma generation and corresponding tacking traits, we assessed all 1540 combinations of up to three carbon and nitrogen sources, chosen from 22 common constituents of yeast growth media. All combinations were ranked for their suitability for positively selecting the flux bases of the target aroma generation (via the tacking traits) using the EvolveX score (supplementary information, Table S1). High-scoring environments were assessed for literature evidence of feasibility of *S. cerevisiae* growth*.* Two of the high-scoring environments, which were among the top 20 of 1171 growth-supporting solutions, were selected for experimental validation. Evolution environment containing glycerol, phenylalanine, and threonine as sole carbon and nitrogen sources was chosen for phenylethyl alcohol and phenylethylacetate production. In this environment, hereafter called glycerol environment (Figure 2A), 7 fluxes (out of 20 in the flux basis) formed the tacking trait of phenylethyl alcohol and phenylethylacetate production (supplementary information, Table S2). For branched-chain amino acid-derived aromas, ethanol environment (Figure 2A), containing ethanol, arginine, and glycine, was selected for experimental validation. In the ethanol environment 11 fluxes (out of 44 in the flux basis) formed the tacking trait (supplementary information, Table S2). The two tacking traits included two common fluxes (transketolase 1, ribulose 5-phosphate epimerase). However, only eight common fluxes were predicted to be positively selected in the two evolution environments while 57 fluxes were predicted to be selected only in one of the two evolution environments (supplementary information, Table S3). Notably, the glycerol environment and in the ethanol environment were predicted to expose positive selection on 17 (out of 29) and 20 (out of 44) common fluxes with intuitive control environments glycerol and ammonium and ethanol and ammonium, respectively (supplementary information, Table S3). Thus, the EvolveX designed glycerol and ethanol evolution environments act as appropriate controls to each other.

Revised text:
The fitness improvement in the glycerol environment was associated with a differential abundance of 48 and 78 metabolic enzymes in G2-1 and G2-2, respectively. In total 139 and 224 proteins were found in differential abundance compared to the parental strain (limma; n=3, P value > 0.01, -1 > log2fc >1) in G1-2 and G2-2, respectively. 66 of the proteins were shared (Figure 3C-D) marking the shared solutions in fitness improvement. Many protein down-regulations were shared between G2-1 and G2-2 (Figure 3C). The metabolic enzymes with

increased abundance were enriched in respiratory pathways in accord with the strong selection pressure predicted by EvolveX (supplementary information, Table S7). A significant overlap was found between the enzymes predicted to be positively selected and the proteins present in higher abundance in the clones evolved in the glycerol environment (hypergeometric test, G2-1 P value 0.000022, G2-2 P value 0.0024). The proteins present in higher abundance in G2-2 overlapped significantly also with the tacking trait (P value 0.021). In addition, glycolytic enzymes (Cdc19, Pdc6, and Tdh1) became less abundant in G2-1, suggesting increased respiratory activity relative to glycolysis.
The fitness improvement in the ethanol environment was also associated with few, focused, enzyme abundance changes (12 in E2-1 and 31 in E2-2; limma, n=3, P value > 0.01, -1 > log2fc >1, supplementary information, Table S7). In total 19 and 68 proteins were found in differential abundance compared to the parental strain in E1-2 and E2-2, respectively. Only eight of these were shared between E2-1 and E2-2 (Figure 3C-D), underscoring the multiple evolutionary solutions to fitness improvement. The metabolic enzymes present in higher abundance in E2-2 significantly overlapped with the enzymes predicted to be positively selected in the ethanol environment (hypergeometric test, P value 0.050). Consistent with arginine as the nitrogen source in this evolution environment, the changes included decreased abundance of arginine biosynthetic pathway enzymes (Arg1 and Arg8 in E2-1, and Arg5,7 in E2-2). Strain E2-2 further had decreased abundance of proline oxidase, Put1, involved in the utilization of one of the four nitrogen atoms in arginine. Several transporters had higher abundance in E2-2: arginine permease (Can1), monocarboxylate transporter (Jen1), methionine permease (Mup1), and hexose transporter (Hxt6). The endocytosis of all these transporters is mediated by Rsp5-Ldb19 (Becuwe & Leon, 2014; Guiney *et al*, 2016; Nikko & Pelham, 2009), which was mutated in the E2-2. Overall, in both evolution environments, the protein abundance changes were limited to the key growth-linked pathways predicted by EvolveX – respiration in the glycerol environment, and arginine metabolism in the ethanol environment.
In the application environment (wine must), the improved aroma generation was accompanied by changes in expression of around 50-200 genes (supplementary information, Table S9). Genes connected to the tacking traits and flux basis were affected, including chorismate synthesis, aromatic amino transferase, and the Ehrlich pathway (Table S2, Table S6). In G2-2, a significant overlap was detected between the corresponding flux basis of the desired trait and the genes found up-regulated (hypergeometric test, P value 0.0052). At protein level, abundance changes (limma; P value > 0.01, -1 > log2fc > 1) were observed in 9 to 32 proteins in the evolved isolates (Figure 3E). A few changes in metabolic enzymes centered on the supply of precursors to the target aroma compounds were observed (2 to 10 enzymes, Figure 3F-G). Significant overlap was detected in proteins found in higher abundance in the evolved clones and the tacking traits of the both aroma profiled evolved clones (G2-1 P value 0.011, G2-2 P value 0.017, E2-1 0.046). In E2-1 also the flux basis excluding the tacking trait overlapped significantly with the proteins in higher abundance than in the parental strain (P value 0.0064). All evolved strains exhibited increased levels of transketolase (Tkl1) consistent with increased precursor supply to aroma biosynthesis as per model prediction. The clones from the glycerol environment showed decreased levels of His1, which competes with Tkl1 for the precursor ribose 5-phosphate (Figure 3F). Another competing pathway, Orotidine-5'-phosphate decarboxylase (Ura3), involved in purine nucleotide synthesis, was also less abundant. In the ethanol environment, increased Tkl1 abundance was accompanied by those of dihydroxyacid dehydratase (Ilv3) and isopropylmalate isomerase (Leu1) (Figure 3G). Both Ilv3 and Leu1 are involved in branched-chain amino acid biosynthesis and higher activities were predicted by the model. Leu2, which follows Leu1 in the leucine biosynthesis pathway, had decreased abundance on one of the clones in accord with the model predictions (Figure 3G, supplementary information, Table S7). Overall, the protein abundance changes in evolved cells were centered on the aroma synthesis pathways consistent with the model predictions.

4. In the methods, some details are missing or not made explicit. For example, in the characterization of evolved strains (page 24; sentence 11) the authors speak of the best growing strain without implying what this is or showing the growth improvement of the best growing strain. The authors state that at the end of the evolution experiment they picked "isolates performing the best" (page 24 sentence 1), without showing or explaining what this is exactly (e.g. increased growth rate). This is relevant information, as the number of generations used is relatively small compared to other studies where 300-500 generations is typically used.

We have now included the growth characterization data (Figure 2C-E, copied below).

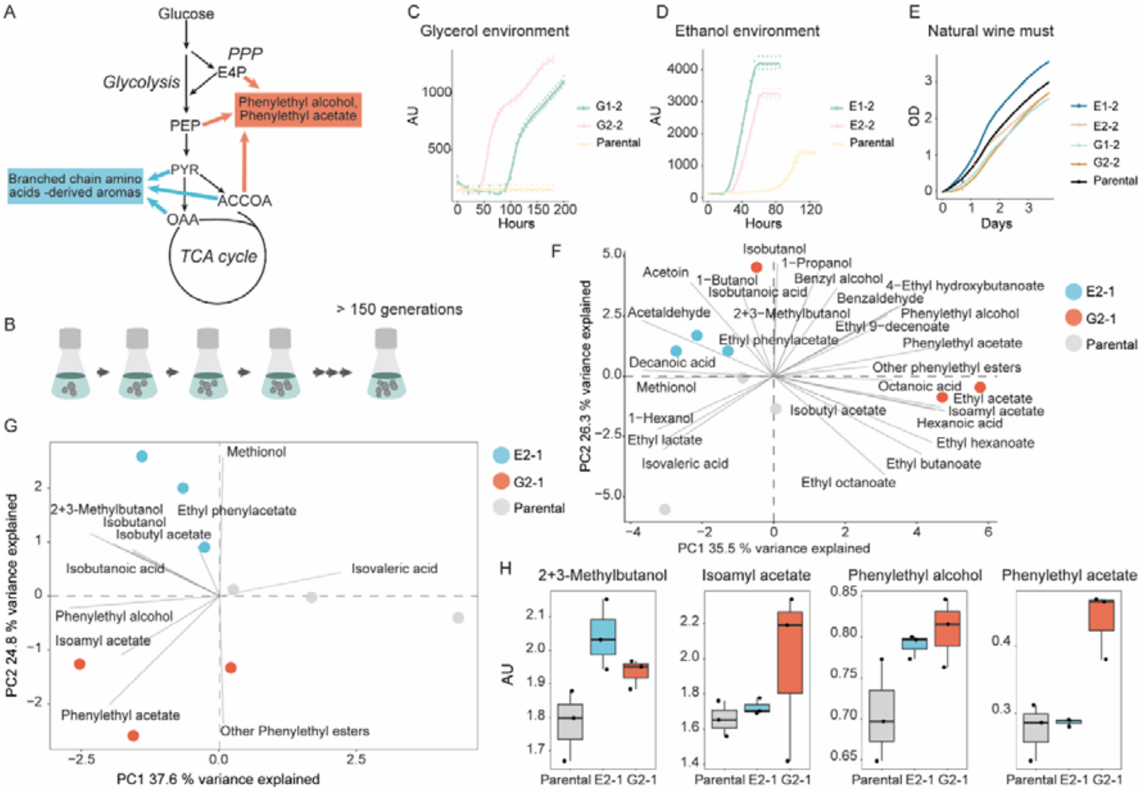

Figure 2. Aroma production changes detected in evolved yeast strains. A) Origin of aroma compounds in the yeast central metabolism: branched-chain amino acid derived compounds (esp. 2-methyl-1-butanol, 3-methyl-1-butanol, isoamyl acetate and 2-methylbutylacetate), and aromatic amino acid derived compounds (esp. phenylethyl alcohol and phenylethyl acetate). Acetate esters of higher alcohols share an acetyl-CoA (ACCOA) precursor. B) Parental wine strain of *S. cerevisiae* was adaptively evolved in both ethanol environment and glycerol environment for over 150 generations. C) Evolved single colony isolates had improved growth in glycerol environment compared to parental. The growth of isolates G1-2 and G2-2 and the parental characterized in three biological replicates as backscattered light (AU – arbitrary units). D) Evolved single colony isolates had improved growth in ethanol environment compared to parental. The growth of isolates E1-2 and E2-2 and the parental characterized in three biological replicates as backscattered light (AU – arbitrary units). E) Evolved single colony isolates maintained similar to parental growth ability characterized in single biological replicates as carbon loss in natural wine must fermentations. F) Principal components analysis of quantified 28 volatile aroma compounds in natural wine must fermentations, with the parental (grey) and evolved strains in three biological replicates. Evolved strain from the ethanol evolution environment (ethanol, arginine, glycine), E2-1, in light blue, and that from the glycerol evolution environment (glycerol, phenylalanine, threonine), G2-1, in orange. G) Principal components analysis of aromatic and branched amino acids-derived volatile compound profiles of natural wine must fermentations, with the parental (grey) and evolved strains (E2-1 in light blue, G2-1 in orange) in three biological replicates. H) Changes in selected aroma compound abundances in wine must fermentations. AU – arbitrary units. E2-1 (light blue) was selected in the ethanol environment, and G2-1 (orange) was selected in the glycerol environment. 2+3-methylbutanol (a combined pool of 2-methyl-1-butanol and 3-methyl-1-butanol) and isoamyl acetate (acetate ester of 3-methyl-1-butanol) were the desired target aromas of the ethanol environment, deriving from branched-chain amino acids. Phenylethyl alcohol and its acetate ester, phenylethyl acetate, were the desired target aromas of the glycerol environment.

Revised text:
In each of the two selected environments, three replicate populations of a diploid wine yeast strain were independently evolved asexually for over 150 generations (Figure 2B). Growth improvement was observed in both evolution environments (Figure 2C-D, supplementary information, Table S4). In the selected isolates evolved in ethanol environment an increase in maximum specific growth rate of over two-fold was estimated (Figure 2D, supplementary information, Table S4). Aroma production and growth physiology of single colony isolates were assessed in natural wine must fermentations (without any aroma precursor supplementation). All evolved isolates maintained their fermentation performance in the natural wine must (Figure 2E, supplementary information, Table S5), indicating their suitability for use in wine fermentations.

Additionally, according to the Materials and Methods the conditions differ between the secondary compound

cultivations and the small-scale fermentations for transcriptomics and proteomics, which is not discussed. These conditions may affect physiology.

The cultivations for aroma profiling and omics analysis were carried out in different laboratories but using the same batch of natural wine must and as non-shaking cultures. Please see the revised paragraphs below.

Revised text:
The clones and the populations selected in the glycerol environment had only few SNVs, and no genes showed recurrent SNVs. However, CNVs were prevalent, with multiple triplicated segments observed in several cases (Figure 3b; supplementary information, Table S4). This extensive variation meant that no particular genes or pathways could be directly linked with either growth or aroma production. Indeed, many of the duplicated genes could be dosage compensated (Muenzner *et al*, 2022). Therefore, we next resorted to analyzing changes at the transcriptomic and proteomic levels. The evolved cells were characterized both in the application environment (wine must, same batch as was used for determining the aroma profiles) and in their corresponding evolution environments (supplementary information, Table S6, Table S7). In all cases, the overlap between transcript-level and protein-level changes was below 6 %, indicating major role of post-transcriptional regulation in both the improved aroma generation in wine must and in the improved fitness in the evolution environments.

Revised text:
A single colony of the parental strain, two evolved isolates originating from the ethanol environment and two evolved isolates originating from the glycerol environment were grown overnight in 50 mL Falcon® tubes with 15 mL of YPD. The overnight grown cells were washed three times with PBS and diluted to an initial OD_{600} of 0.1 in 55 mL of natural white must from the 2017 harvest (see above, the same natural white must batch used as for aroma profiling). For the microvinification process, 50 mL Erlenmeyer flaks were used, filled to the maximum, in order to create microanaerobic conditions. Maintaining the anaerobic conditions meant that the growth could not be estimated based on changes in the optical density, but it was correlated with the observed weight loss, which occurs from the release of CO_2, the end product of carbon metabolism. Release of CO_2 is possible through a small needle which is pierced through rubber plugs, which in turn were sterilized and used to seal the Erlenmeyer flaks, while a small piece of gauge prevents anything from the environment to fall inside the flask through the needle. The growth stage of the cultures was estimated based on weight loss which correlates to the consumption of glucose and release of CO_2 as suggested by (Harsch *et al*, 2010). For this reason, the initial weight of the cultures was measured and followed once every day until no more weight loss was observed, at which point the cultures had entered stationary phase. After the establishment of the growth kinetics with weight loss, same cultures as described above were prepared, weight loss was once again followed and the cells were harvested at mid exponential phase for RNA-sequencing and proteomics analysis.

Minor points

Figure 3 (a,b) contains a lot of clones annotated in a similar fashion (e.g. E1, G1, etc.). It is not made clear what the exact distinction is between these clones and if more data is available for them.

We thank the reviewer for pointing out this unclarity in Figure 3. We have revised the description of the naming scheme and moved it up in the figure legend (copied below).

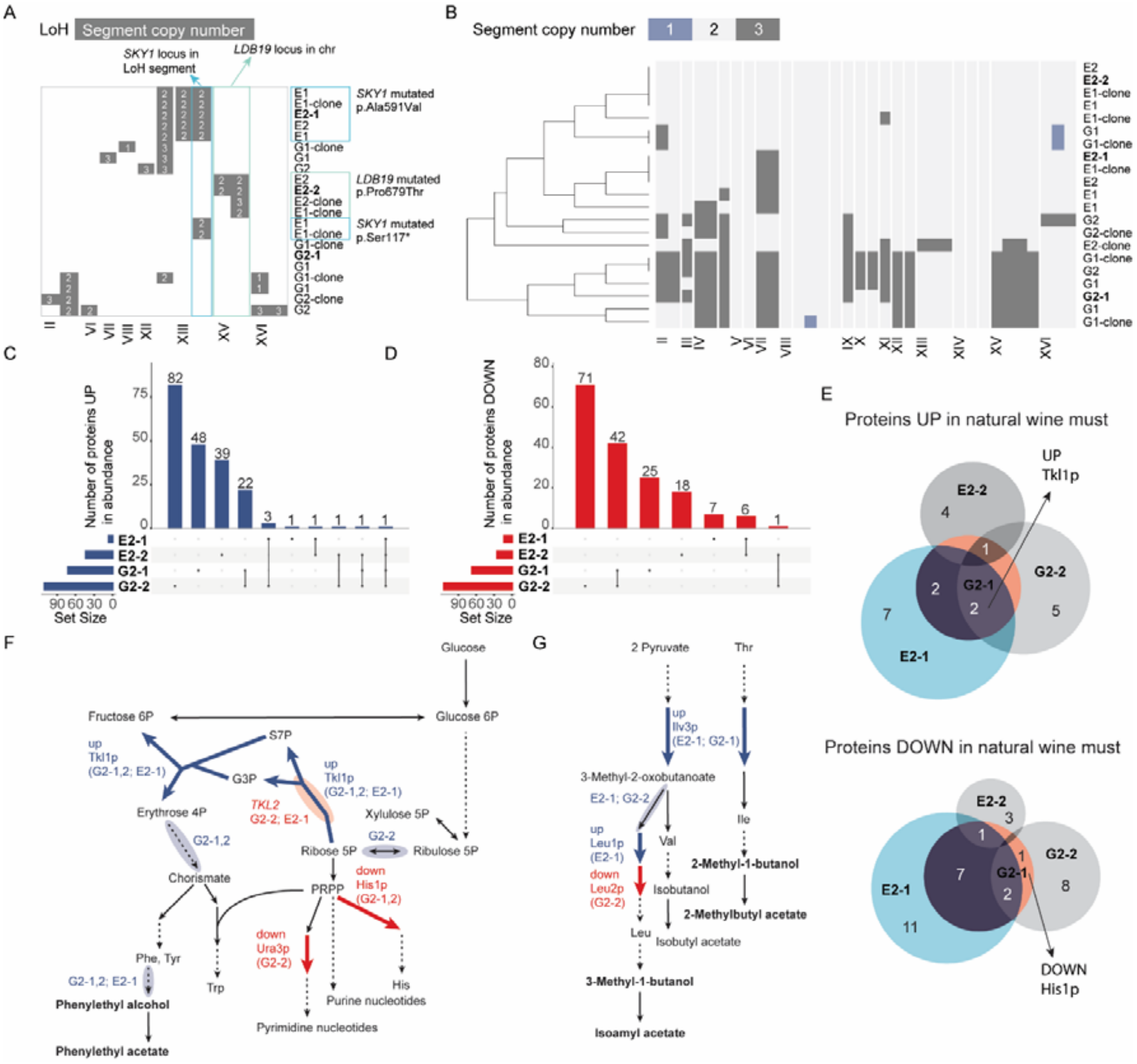

Figure 3. Molecular changes detected in evolved yeast strains. A) Loss-of-heterozygosity (LoH) coincided with single nucleotide variants (SNVs, marked on the top and right side of the panel) in evolved populations and clones from the ethanol environment, suggesting the necessity of the SNVs being homozygous for the evolved phenotype. The evolved clones ("-clone") and populations are named according to their selection environment: 'G' – glycerol selection environment. 'E' – ethanol selection environment. The number after the letter stands for the evolution status: 1 – the time of first isolation of clones, 2 – the time of second isolation of clones. The clones for which we determined protein and transcript alterations are indicated in bold. B) Evolved populations and clones from the glycerol environment exhibited large copy number variations. Shown are the genome segment copy numbers along the chromosomes. Vertical lines mark ends of contigs. C) Upset plot of sets of proteins higher in abundance (limma, n=3 (biological replicates), *P value* < 0.01, -1 > log2fc > 1) in the evolved isolates than in the parental strain in the respective evolution environments (G1-2, G2-2: glycerol environment; E1-2, E2-2 ethanol environment) shows partly shared solutions underlying improved fitness. D) Upset plot of sets of proteins lower in abundance (limma, n=3 (biological replicates), *P value* < 0.01, -1 > log2fc > 1) in the evolved isolates than in the parental strain in the respective evolution environments (G1-2, G2-2: glycerol environment; E1-2, E2-2 ethanol environment) shows proportionally large overlaps between the isolates evolved in the same environment. E) The evolved clones fermenting natural wine must (application environment) revealed both shared and evolution environment-specific protein abundance changes up and down in comparison to the parental strain (limma, n=3 (biological replicates), *P value* < 0.01, -1 > log2fc > 1). Clones for which we quantified the aroma production are shown in color (E2-1 in light blue, G2-1 in orange). Clones from the glycerol environment (G2-1, G2-2) featured higher abundance of Tkl1p (transketolase) and lower abundance of His1p (ATP phosphoribosyltransferase). F) Changes in protein (limma; n=3 (biological replicates), *P value* < 0.01, -1 > log2fc > 1) and transcript abundances (Wald test; n=3 (biological replicates), fdr < 0.05, -1 > log2 fc > 1) are centered on the pathways leading to the target aroma compounds phenylethyl alcohol and phenylethyl acetate. The changes consistent with the model predictions are indicated with colored arrows (protein-level) and clouds

around the arrows (transcript-level). G) Proteomic and transcriptomic changes in evolved clones, marked as in F), for pathways leading to the branched chain amino acids derived target aroma compounds.

Page 6 line 9: Many of the aroma compounds mentioned contain only carbon. I understand they are often derived from amino acids but not all readers of MSB will understand this. Please explain.

We agree and have added this explanation in the revised manuscript (revised text below).

Revised text:

We targeted two main groups of aroma compounds: i) phenylethyl alcohol and its acetate ester, phenylethylacetate, which have a rose and honey scent and raspberry-like flavor; and ii) branched-chain amino acid-derived higher alcohols (2-methyl-1-butanol and 3-methyl-1-butanol) and their acetate esters (2-methylbutylacetate and isoamyl acetate) (Carpena *et al*, 2021; Swiegers *et al*, 2005), which have a banana and pear scent and fruity flavor. All these aroma compounds derive from amino acids' (L-phenylalanine and branched chain amino acids) carbon backbones and contain no nitrogen. The flux bases of the target aroma syntheses were defined as a minimum set of fluxes that have to increase for the particular target aroma generation to be enhanced. Similarly, flux bases could include fluxes that should be negatively selected for desired trait development.

Page 7, line 20 onwards: since you measured both populations and isolates, please make sure that in the paragraph that follows it is always clear which genetic variation belongs to which sample.

We thank the reviewer for pointing this out. The corresponding text is now revised to indicate when the variants were detected in the populations and clones.

Revised text:

In the case of ethanol environment, copy number variants (CNVs) analysis revealed triplications of chromosome VII in several evolved populations and isolates (supplementary material, Table S7). Further, recurrently in several populations and clones single nucleotide variants (SNVs) were found in *SKY1*, which encodes a serine/threonine kinase involved in the regulation of polyamine transport (missense p.Ala591Val, frameshift p.Leu64fs, stop gain p.Ser117*) (supplementary information, Table S8). We also observed a loss-of-heterozygosity segment in the contig containing the *SKY1* locus (supplementary information, Table S7). *SKY1* deletion gives yeast tolerance to high spermine concentrations (Erez & Kahana, 2001), a degradation product of arginine, which was one of the three components in the ethanol environment. In clones where *SKY1* mutations were not detected, we found paired missense mutations in genes encoding the ubiquitin ligase Rsp5 (p.Arg355Gly) and its target-guide and adapter Ldb19 (p.Pro679Thr), which drive the endocytosis of plasma membrane-localized amino-acid transporters. Ldb19 variant was accompanied with a loss-of-heterozygosity in the contig containing the locus (Figure 3A; supplementary information, Table S7). Thus, in the ethanol environment, mutations in genes involved in arginine utilization were enriched in accord with the selection regime.

The clones and the populations selected in the glycerol environment had only few SNVs, and no genes showed recurrent SNVs. However, CNVs were prevalent, with multiple triplicated segments observed in several cases (Figure 3b; supplementary information, Table S4). This extensive variation meant that no particular genes or pathways could be directly linked with either growth or aroma production. Indeed, many of the duplicated genes could be dosage compensated (Muenzner *et al*, 2022). Therefore, we next resorted to analyzing changes at the transcriptomic and proteomic levels. The evolved cells were characterized both in the application environment (wine must, same batch as was used for determining the aroma profiles) and in their corresponding evolution environments (supplementary information, Table S6, Table S7). In all cases, the overlap between transcript-level and protein-level changes was below 6 %, indicating major role of post-transcriptional regulation in both the improved aroma generation in wine must and in the improved fitness in the evolution environments.

Page 9, sentence 22 "... rooted in the laws of thermodynamics..." . It is not clear where that comes from and is confusing.

We have revised the unclear sentence:

Revised text:

The EvolveX algorithm, with roots in the laws of thermodynamics as captured by genome-scale metabolic models, allowed us to predict the environment-dependent trait-fitness correlations. Our theory and results thus bring predictive evolution, which has yet mostly based on empirical correlations, in the realm of first-principles modelling. Previously, adaptive evolution of fitness-beneficial traits in one niche has been shown to facilitate exaptation, i.e., the predisposition to fitness improvement in another niche (Szappanos et al., 2016). In contrast, we propose and show that, in an appropriately chosen evolution environment, a trait without a fitness benefit in the application environment can adaptively evolve.

Within the discussion (page 10 sentence 8), it is claimed "the need to know" is circumvented. Yet the authors use a well-curated genome-scale metabolic model and well-studied pathways. They could be more subtle and try to explain these potential hurdles in their discussion. They also came across the limitation of strain to strain differences in selection of wine strains for their experiments, which is mentioned in the materials and methods. In

addition (page 10 sentence 15), the authors claim that their method could help to understand complex adaptive processed, yet mechanistic understanding of their own study is minimal.

Indeed, the knowledge of the metabolic network structure is a prerequisite for application of EvolveX. What we meant is that it is not necessary to know how the distribution of flux is regulated. Thus, the knowledge of the genetic/epigenetic underpinnings of the fluxes, such as transcriptional and post-translational or allosteric regulation or an interplay with other cellular regulation like osmoregulation, are not required. We have revised the discussion accordingly (revised paragraph reproduced below). We also included a clarification that genome-scale metabolic models can be automatically reconstructed from genome data but the parts of metabolism including less well characterized enzymes are likely to be inaccurately modelled due to the current limitations in protein functional annotations. In this study, we used the reference strain model to represent our wine yeast parental strain, but the model reconstruction could also be performed for each strain separately.

We chose a parental strain that grew in a reasonable timeframe in our selected evolution environments. However, the method can be applied to any strain that can grow, no matter how slow, in the evolution environment. We have clarified this in the revised manuscript text.

Finally, we respectfully disagree with the reviewer regarding the lack of mechanistic understanding from our study. Studies as ours that have resolved the evolved phenotypes at different levels of cellular regulation (i.e., genotype, gene expression, protein abundances, and metabolites) are rare. Thus, our method provides insights into mechanisms of flux regulation.

Revised text:
The use of model-designed evolution environment maintains the key advantage of adaptive laboratory evolution, viz. circumventing the need to know, except for the basic metabolic network structure, the genetic and regulatory basis of the traits of interest. Indeed, the omics analysis of the improved aroma generation traits in our case study revealed complex genotype-phenotype relationships. Improving these traits using rational strain improvement would currently be challenging (Hassing *et al*, 2019). As genome-scale metabolic models are becoming easier to reconstruct (Machado *et al*, 2018; Pitkanen *et al*, 2014; Seaver *et al*, 2021; Wang *et al*, 2018), our approach can be readily applied to any organism amenable for experimental evolution. Commonly a sufficient quality network is obtained in automatic model reconstruction though the accuracy is the most dependent on the success of protein functional annotation still challenging for less well characterized metabolic enzymes. In this study, we used the *S. cerevisiae* reference strain genome-scale metabolic model to represent our wine yeast parental strain. This demonstrates that the method is not sensitive to differences beyond central pathways when the target compounds originate from the central pathways too. While the choice of the parental wine strain was made based on growth in our selected evolution environments, the EvolveX method is applicable to any strain that can divide in the evolution environment.

Reviewer #2:
The authors present and validate a novel computational approach (EvolveX) to design laboratory evolution experiments to select microbial strains with enhanced metabolite secretion phenotypes. The idea of the method is to circumvent the trade-off between growth and secretion by adapting to an environment where growth becomes correlated with traits that underlie the desired secretion phenotypes. For this, they utilize the constrained-based framework of genome-scale metabolic modelling to predict specific environments where laboratory evolution should be carried out. By focusing on two groups of aroma compounds, they experimentally validate the method in budding yeast.
The work goes beyond the state of the art and is an important step towards employing model-based evolutionary predictions in biotechnology. More broadly, the work also has relevance to our understanding of trait-trait correlations and their environmental dependence. Thus, the work has a high potential to be of broad interest and utility.
However, I have a coupled of concerns surrounding the experimental validation of the method.

Major points:
1) The step-by-step description of EvolveX in the main text and the justification of each step are not crystal clear. For example, how is flux coupling, as defined by Maranas et al. (Burgard et al. 2004), actually used? It is often mentioned, but its usage was not clear to me from the 4 steps described. I first thought it was used to define the flux basis, but the Method section suggests otherwise.

We thank the reviewer for pointing to this unclarity. We have revised the corresponding paragraph in the methods section to clarify that the concept of flux coupling is used to identify which reactions in the flux bases are growth coupled in the evolution environment.

Revised text:
The total response to selection of the desired trait (in worst-case scenario) was predicted as the sum of flux couplings of its flux basis with growth. For the subsets of the flux basis associated with flux change directions up and down, the minimum and maximum growth coupled fluxes, respectively, were summed under the constraint of minimized total nutrient uptake flux for an arbitrary unit of growth (Equation 7). Thus, the concept of flux coupling (Burgard et al, 2004) was used to identify which reactions in the flux bases are growth coupled in the evolution environment.

Also, what does 'response to selection' mean in the computational model? Is it presence of flux coupling or extent of flux correlation? And what's exactly the justification of Eq 4? Why should higher flux per unit of growth correspond to higher relative (proportional?) response to selection? Isn't the covariance what matters for selection response?

Flux coupling is a measure of flux covariance. According to the theory, a trait's 'response to selection' equals the covariance between the trait and fitness. For the growth flux (a proxy for fitness) to increase, the coupled fluxes must increase relative to the strength of their coupling to growth. Since the different media would have different absolute growth rate, the comparison is possible only in a growth-normalized space. Since the flux coupling relations scale linearly, this normalization does not affect the relative coupling strengths. This is the justification for Equation 4, and thus, it is not only the presence of flux coupling but magnitude of it that determines the response to selection. We have revised the corresponding manuscript text to clarify this. The equations related to the evolution theory are in addition placed in a separate box. Please, see these revisions copied below.

Revised text:

Box1. Trait-fitness dependences predictable as flux couplings

The selection acting on a phenotypic trait is the covariance between the trait and the relative fitness, as described by Robertson-Price identity (Price, 1970; Rausher, 1992; Robertson, 1966, 1968) (Equation 1).

$$s = cov(w, z) \quad \text{Equation 1}$$

where s is the selection differential, w fitness, and z the trait of interest.

When there is genetic covariance between the trait and relative fitness, evolutionary response to selection can occur (Equation 2, the secondary theorem of selection).

$$R = s_g = cov_a(w, z) \quad \text{Equation 2}$$

where R is the response to selection with units of the trait and fitness multiplied, sg is the genetic selection differential, and $cov_a(w,z)$ is the additive genetic covariance.

Equation 2 generalizes to a multivariate form for multiple traits (Rausher, 1992).

$$\boldsymbol{R} = cov_a(w, \boldsymbol{z}) \quad \text{Equation3}$$

We now consider the case of metabolic traits, which can be represented and modelled as a set of metabolic fluxes (net reaction rates). Metabolic trait interdependencies under a given chemical environment can then be predicted using genome-scale metabolic models as flux couplings (Burgard *et al*, 2004). Two metabolic reactions are coupled if a non-zero flux through one reaction implies a non-zero flux through the other. Flux covariance follows from flux coupling (Heinonen *et al*, 2019; Pradhan, 2019; Thommes *et al*, 2019). Importantly for modelling evolutionary adaptation, flux coupling implies genetic dependences between the corresponding enzyme-coding genes (Notebaart *et al*, 2008).

To predict relative responses of a metabolic trait to selection, we use its coupling to the specific growth rate (proxy for mean fitness). Analogous to the secondary theorem of selection (Equation 3), this gives:

$$\boldsymbol{F}_v = \frac{\boldsymbol{v}}{\mu} \quad \text{Equation 4}$$

where $\mathbf{F}_v$ is the relative unitless responses of single-flux metabolic traits to selection, v the metabolic fluxes, and μ the specific growth rate. Thus, higher the flux per growth unit, stronger the selection.

To search for a suitable evolution environment, i.e., a defined chemical environment in which the adaptive evolution is to take place, we use the basis provided by the selection response relation (Equations 3, 4). Ideally, the evolution environment would be chosen such that there is a direct selection for the desired trait through flux coupling with the cell growth. This, however, will only rarely be possible as most desired traits, such as metabolite secretion, are at a trade-off with cell growth due to competition for metabolic precursors and co-factors (Jouhten *et al*, 2016; Nielsen & Keasling, 2016) (Figure 1A). We therefore aim at growth coupling of a secondary trait, which we term tacking trait. Tacking trait is here defined as a set of fluxes that are flux coupled (Burgard *et al.*, 2004) to cell growth in the evolution environment, and with the desired trait in the application environment (Figure

1B). We note that it is neither necessary for the tacking trait to be coupled with the desired trait in the evolution environment, nor it is likely due to the trade-off with growth. Further, the tacking trait is necessarily a proper subset of fluxes that must increase or decrease for the desired trait enhancement in the application environment. Due to the environment-dependence of genetic correlations between traits (Equation 3), the tacking trait and the evolution environment are intrinsically linked and need to be identified simultaneously.
A desired trait that does not pose a fitness advantage will not be under Darwinian selection in the application environment (Figure 1A). In our strategy, the evolution environment is designed such that the tacking trait becomes flux coupled to mean fitness (Figure 1B), allowing positive selection on *de novo* mutations enhancing the tacking trait. Upon switching to the application environment, in which the tacking trait is flux coupled with the desired trait, the latter is enhanced (Figure 1C, D). To illustrate this strategy, we consider a simple metabolic network (Figure 1E-G). The parental strain is well adapted to channel the nutrients to cell growth and thus produces only a little desired product (Figure 1E). In an appropriately selected evolution environment (Figure 1F), a different set of pathways are flux coupled with growth (Figure 1F). During the adaptive evolution, increased flux through these growth-coupled pathways is selected for. While there is no increase of production in the evolution environment, the evolved strain exhibits, due to the direct coupling between the tacking and the target trait, improved production in the application environment (Figure 1G).

2) To experimentally demonstrate that specific aroma compounds are secreted at increased rates after selection in the predicted environments, the authors compare the evolved lines to their ancestors and to each other. However, I couldn't find any negative controls for the evolutionary experiments. It might be possible that adaptation to various different nutrient environments enhances aroma compound production as pleiotropic effects and so the observed secretion phenotypes are not specific. While I wouldn't request to carry out new lab evolution, I encourage the authors to measure, if possible, the aroma production of strains that were evolved in the laboratory in similar studies but for other goals (i.e. better growth on glucose, increased heat tolerance, etc.). Alternatively, it would be informative to comparing the compound productions with those of other wine strains. Such comparisons could support the uniqueness of the aroma production profile achieved by lab evolution.

We thank the reviewer for raising these points. The two selected evolution environments do not have equal sets of fluxes coupled to growth. Thus, they act as controls for each other for differential selection on fluxes. We have now clarified this in the revised manuscript text (copied below).

We have now included the data on all the 28 volatile compounds, quantified in cultures grown on natural grape must, in the supplementary material and included a visualization of the PCA in Figure 2F (reproduced below, also accordingly revised Results section reproduced below). When all 28 compounds are considered, the parental and evolved strains do not cluster separately (Figure 2F); however, when considering the target compounds (Figure 2G), the variance leads to expected clustering driven by the respective compound profiles. This supports a degree of selectivity in the aroma changes. The changes observed in other, non-target, compounds, are small with the evolved lineages being in the range of parental variation.

We observed changes shared between the isolates selected in the two different evolution environments. This is expected since the tacking traits of the two sets of target aroma were partially overlapping for the aromatic amino acids and branched chain amino acids derived target aromas, by 2 fluxes out of 7 and 11, respectively. We have clarified this in the revised manuscript (revised text reproduced below). We also note that in this study we did not directly optimize the evolution environments for specificity of the desired compound generation (but summed tacking trait flux couplings with growth). Thus, changes in target compounds as well as in other compounds is consistent with the design. We have clarified this in the revised manuscript text (please see the revised paragraph below).

Revised text:
To identify a suitable evolution environment for enhancing the target aroma generation and corresponding tacking traits, we assessed all 1540 combinations of up to three carbon and nitrogen sources, chosen from 22 common constituents of yeast growth media. All combinations were ranked for their suitability for positively selecting the flux bases of the target aroma generation (via the tacking traits) using the EvolveX score (supplementary information, Table S1). High-scoring environments were assessed for literature evidence of feasibility of *S. cerevisiae* growth*.* Two of the high-scoring environments, which were among the top 20 of 1171 growth-supporting solutions, were selected for experimental validation. Evolution environment containing glycerol, phenylalanine, and threonine as sole carbon and nitrogen sources was chosen for phenylethyl alcohol

and phenylethylacetate production. In this environment, hereafter called glycerol environment (Figure 2A), 7 fluxes (out of 20 in the flux basis) formed the tacking trait of phenylethyl alcohol and phenylethylacetate production (supplementary information, Table S2). For branched-chain amino acid-derived aromas, ethanol environment (Figure 2A), containing ethanol, arginine, and glycine, was selected for experimental validation. In the ethanol environment 11 fluxes (out of 44 in the flux basis) formed the tacking trait (supplementary information, Table S2). The two tacking traits included two common fluxes (transketolase 1, ribulose 5-phosphate epimerase). However, only eight common fluxes were predicted to be positively selected in the two evolution environments while 57 fluxes were predicted to be selected only in one of the two evolution environments (supplementary information, Table S3). Notably, the glycerol environment and in the ethanol environment were predicted to expose positive selection on 17 (out of 29) and 20 (out of 44) common fluxes with intuitive control environments glycerol and ammonium and ethanol and ammonium, respectively (supplementary information, Table S3). Thus, the EvolveX designed glycerol and ethanol evolution environments act as appropriate controls to each other.

Revised text:
Mass-spectrometry analysis of the volatile compounds (28 quantified, supplementary information, Table S6) in wine must fermentations with the parental strain and evolved isolates provided a view on the changes in volatiles following evolution. In principal components analysis, the strains did not cluster by their history (Figure 2F), supporting that the volatile metabolite production was not universally impacted following laboratory evolution However, the principal components analysis considering only the target compounds, the aroma profiles clustered by the evolution environment and separately from the parental (Figure 2G). The first principal component (PC1, 37.6 % of total variance) distinguished parental from the evolved strains. In accordance with the model, this separation is driven by the overlap of the two tacking traits (transketolase and ribulose 5-phosphate 3-epimerase fluxes; supplementary information, Table S2). Further attesting the model, the isolates selected in the ethanol and glycerol evolution environments were separated mainly by the target aroma compounds (PC2, 24.8 % of total variance, Figure 2G-H). While for target aroma compound isoamyl acetate we could not validate the model predictions (i.e. level similar to parental in fermentations with E2-1), phenylethylacetate was specifically increased in the wine must fermentations with the isolates selected in the glycerol environment (Figure 2H). Similarly, the combined pool of branched-chain amino acid-derived aroma compounds 2-methyl-1-butanol and 3-methyl-1-butanol was increased only for the isolate selected in the ethanol environment. Together, evolved isolates featured increased aroma formation in wine must according to the EvolveX predictions.

3) How similar are the aroma production profiles of the independently evolved parallel lines that were exposed to the same selection pressure? Are they more similar within evolution environment than lines evolved in different environments? I suggest to show all parallel lines on the PCA plot (Fig 2b).

We evolved three lineages in each evolution environment but due to the constraints on vinification experiments, we characterized the aroma profiles only for a single clone from each evolution environment, that showed growth performance as the lineage. We have now included a visualization of the profiles of all quantified aroma compounds (Figure 2F, please, see the revised figure and the legend below). This visualization includes three biological replicates of the small-scale vinification experiment.

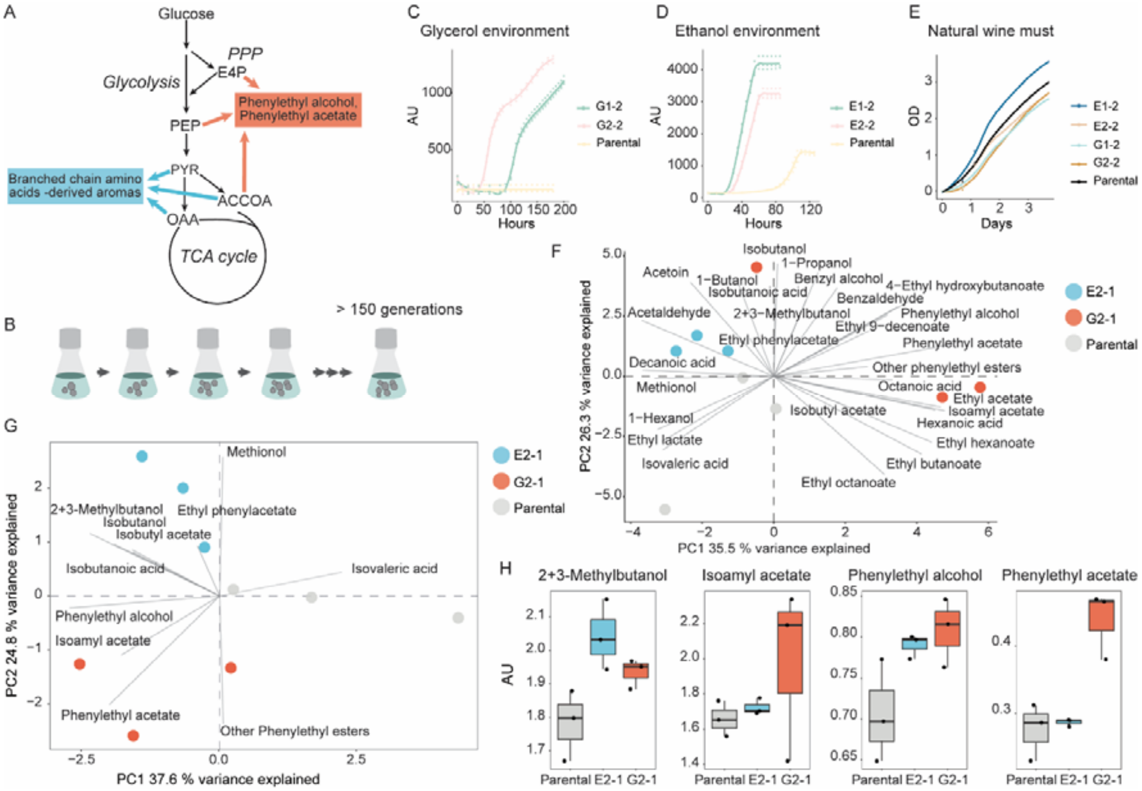

Figure 2. Aroma production changes detected in evolved yeast strains. A) Origin of aroma compounds in the yeast central metabolism: branched-chain amino acid derived compounds (esp. 2-methyl-1-butanol, 3-methyl-1-butanol, isoamyl acetate and 2-methylbutylacetate), and aromatic amino acid derived compounds (esp. phenylethyl alcohol and phenylethyl acetate). Acetate esters of higher alcohols share an acetyl-CoA (ACCOA) precursor. B) Parental wine strain of *S. cerevisiae* was adaptively evolved in both ethanol environment and glycerol environment for over 150 generations. C) Evolved single colony isolates had improved growth in glycerol environment compared to parental. The growth of isolates G1-2 and G2-2 and the parental characterized in three biological replicates as backscattered light (AU – arbitrary units). D) Evolved single colony isolates had improved growth in ethanol environment compared to parental. The growth of isolates E1-2 and E2-2 and the parental characterized in three biological replicates as backscattered light (AU – arbitrary units). E) Evolved single colony isolates maintained similar to parental growth ability characterized in single biological replicates as carbon loss in natural wine must fermentations. F) Principal components analysis of quantified 28 volatile aroma compounds in natural wine must fermentations, with the parental (grey) and evolved strains in three biological replicates. Evolved strain from the ethanol evolution environment (ethanol, arginine, glycine), E2-1, in light blue, and that from the glycerol evolution environment (glycerol, phenylalanine, threonine), G2-1, in orange. G) Principal components analysis of aromatic and branched amino acids-derived volatile compound profiles of natural wine must fermentations, with the parental (grey) and evolved strains (E2-1 in light blue, G2-1 in orange) in three biological replicates. H) Changes in selected aroma compound abundances in wine must fermentations. AU – arbitrary units. E2-1 (light blue) was selected in the ethanol environment, and G2-1 (orange) was selected in the glycerol environment. 2+3-methylbutanol (a combined pool of 2-methyl-1-butanol and 3-methyl-1-butanol) and isoamyl acetate (acetate ester of 3-methyl-1-butanol) were the desired target aromas of the ethanol environment, deriving from branched-chain amino acids. Phenylethyl alcohol and its acetate ester, phenylethyl acetate, were the desired target aromas of the glycerol environment.

Similar question applies to similarities in transcriptome and proteome profiles. Do the two selection regimes result in distinct omics changes that are repeatedly observed among parallel evolved lines? One would expect clear physiological changes that are specific to the selection regime. Btw, I suggest to report full transcriptome and proteome profiles (i.e. including genes showing no differential expression) as Supplementary Tables.

We have now supplemented the full transcriptome and proteome profiles of the evolved clones (two from each evolution environment) and the parental strain in three biological replicates. Though the phenotypic evolution, including physiological state, is expected to converge during adaptive evolution, the genotype evolution is stochastic. Therefore, overlap in the transcriptome or proteome levels is not necessarily expected (same flux state can be

obtained through different regulatory mechanisms). We characterized two evolved isolates from each evolution environment to understand how their phenotypes observed came about. We have revised the manuscript text to clarify this and augmented Figure 3 with upset plots (Figure 3C-D) for visualizing shared and specific protein changes in evolved clones (please, see the revised Figure 3 and paragraphs copied below).

Revised text:
The fitness improvement in the glycerol environment was associated with a differential abundance of 48 and 78 metabolic enzymes in G2-1 and G2-2, respectively. In total 139 and 224 proteins were found in differential abundance compared to the parental strain (limma; n=3, P value > 0.01, -1 > log2fc >1) in G1-2 and G2-2, respectively. 66 of the proteins were shared (Figure 3C-D) marking the shared solutions in fitness improvement. Many protein down-regulations were shared between G2-1 and G2-2 (Figure 3C). The metabolic enzymes with increased abundance were enriched in respiratory pathways in accord with the strong selection pressure predicted by EvolveX (supplementary information, Table S7). A significant overlap was found between the enzymes predicted to be positively selected and the proteins present in higher abundance in the clones evolved in the glycerol environment (hypergeometric test, G2-1 P value 0.000022, G2-2 P value 0.0024). The proteins present in higher abundance in G2-2 overlapped significantly also with the tacking trait (P value 0.021). In addition, glycolytic enzymes (Cdc19, Pdc6, and Tdh1) became less abundant in G2-1, suggesting increased respiratory activity relative to glycolysis.
The fitness improvement in the ethanol environment was also associated with few, focused, enzyme abundance changes (12 in E2-1 and 31 in E2-2; limma, n=3, P value > 0.01, -1 > log2fc >1, supplementary information, Table S7). In total 19 and 68 proteins were found in differential abundance compared to the parental strain in E1-2 and E2-2, respectively. Only eight of these were shared between E2-1 and E2-2 (Figure 3C-D), underscoring the multiple evolutionary solutions to fitness improvement. The metabolic enzymes present in higher abundance in E2-2 significantly overlapped with the enzymes predicted to be positively selected in the ethanol environment (hypergeometric test, P value 0.050). Consistent with arginine as the nitrogen source in this evolution environment, the changes included decreased abundance of arginine biosynthetic pathway enzymes (Arg1 and Arg8 in E2-1, and Arg5,7 in E2-2). Strain E2-2 further had decreased abundance of proline oxidase, Put1, involved in the utilization of one of the four nitrogen atoms in arginine. Several transporters had higher abundance in E2-2: arginine permease (Can1), monocarboxylate transporter (Jen1), methionine permease (Mup1), and hexose transporter (Hxt6). The endocytosis of all these transporters is mediated by Rsp5-Ldb19 (Becuwe & Leon, 2014; Guiney *et al*, 2016; Nikko & Pelham, 2009), which was mutated in the E2-2. Overall, in both evolution environments, the protein abundance changes were limited to the key growth-linked pathways predicted by EvolveX – respiration in the glycerol environment, and arginine metabolism in the ethanol environment.
In the application environment (wine must), the improved aroma generation was accompanied by changes in expression of around 50-200 genes (supplementary information, Table S9). Genes connected to the tacking traits and flux basis were affected, including chorismate synthesis, aromatic amino transferase, and the Ehrlich pathway (Table S2, Table S6). In G2-2, a significant overlap was detected between the corresponding flux basis of the desired trait and the genes found up-regulated (hypergeometric test, P value 0.0052). At protein level, abundance changes (limma; P value > 0.01, -1 > log2fc > 1) were observed in 9 to 32 proteins in the evolved isolates (Figure 3E). A few changes in metabolic enzymes centered on the supply of precursors to the target aroma compounds were observed (2 to 10 enzymes, Figure 3F-G). Significant overlap was detected in proteins found in higher abundance in the evolved clones and the tacking traits of the both aroma profiled evolved clones (G2-1 P value 0.011, G2-2 P value 0.017, E2-1 0.046). In E2-1 also the flux basis excluding the tacking trait overlapped significantly with the proteins in higher abundance than in the parental strain (P value 0.0064). All evolved strains exhibited increased levels of transketolase (Tkl1) consistent with increased precursor supply to aroma biosynthesis as per model prediction. The clones from the glycerol environment showed decreased levels of His1, which competes with Tkl1 for the precursor ribose 5-phosphate (Figure 3F). Another competing pathway, Orotidine-5'-phosphate decarboxylase (Ura3), involved in purine nucleotide synthesis, was also less abundant. In the ethanol environment, increased Tkl1 abundance was accompanied by those of dihydroxyacid dehydratase (Ilv3) and isopropylmalate isomerase (Leu1) (Figure 3G). Both Ilv3 and Leu1 are involved in branched-chain amino acid biosynthesis and higher activities were predicted by the model. Leu2, which follows Leu1 in the leucine biosynthesis pathway, had decreased abundance on one of the clones in accord with the model predictions (Figure 3G, supplementary information, Table S7). Overall, the protein abundance changes in evolved cells were centered on the aroma synthesis pathways consistent with the model predictions.

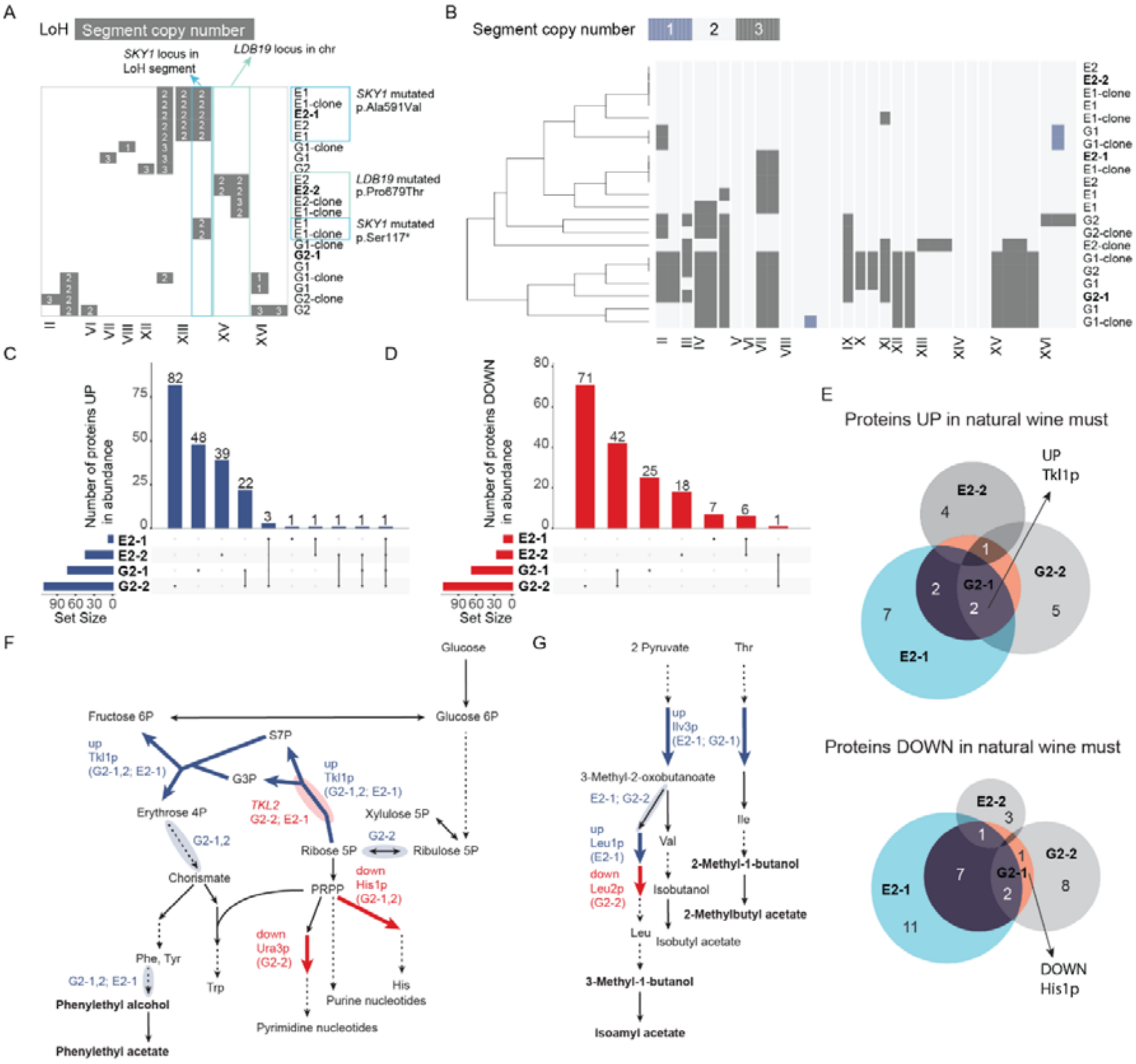

Figure 3. Molecular changes detected in evolved yeast strains. A) Loss-of-heterozygosity (LoH) coincided with single nucleotide variants (SNVs, marked on the top and right side of the panel) in evolved populations and clones from the ethanol environment, suggesting the necessity of the SNVs being homozygous for the evolved phenotype. The evolved clones ("-clone") and populations are named according to their selection environment: 'G' – glycerol selection environment. 'E' – ethanol selection environment. The number after the letter stands for the evolution status: 1 – the time of first isolation of clones, 2 – the time of second isolation of clones. The clones for which we determined protein and transcript alterations are indicated in bold. B) Evolved populations and clones from the glycerol environment exhibited large copy number variations. Shown are the genome segment copy numbers along the chromosomes. Vertical lines mark ends of contigs. C) Upset plot of sets of proteins higher in abundance (limma, n=3 (biological replicates), *P value* < 0.01, -1 > log2fc > 1) in the evolved isolates than in the parental strain in the respective evolution environments (G1-2, G2-2: glycerol environment; E1-2, E2-2 ethanol environment) shows partly shared solutions underlying improved fitness. D) Upset plot of sets of proteins lower in abundance (limma, n=3 (biological replicates), *P value* < 0.01, -1 > log2fc > 1) in the evolved isolates than in the parental strain in the respective evolution environments (G1-2, G2-2: glycerol environment; E1-2, E2-2 ethanol environment) shows proportionally large overlaps between the isolates evolved in the same environment. E) The evolved clones fermenting natural wine must (application environment) revealed both shared and evolution environment-specific protein abundance changes up and down in comparison to the parental strain (limma, n=3 (biological replicates), *P value* < 0.01, -1 > log2fc > 1). Clones for which we measured the aroma production are shown in color (E2-1 in light blue, G2-1 in orange). Clones from the glycerol environment (G2-1, G2-2) featured higher abundance of Tkl1p (transketolase) and lower abundance of His1p (ATP phosphoribosyltransferase). F) Changes in protein (limma; n=3 (biological replicates), *P value* < 0.01, -1 > log2fc > 1) and transcript abundances (Wald test; n=3 (biological replicates), fdr < 0.05, -1 > log2 fc > 1) are centered on the pathways leading to the target aroma compounds phenylethyl alcohol and phenylethyl acetate. The changes consistent with the model predictions are indicated with colored arrows (protein-level) and clouds

around the arrows (transcript-level). G) Proteomic and transcriptomic changes in evolved clones, marked as in F), for pathways leading to the branched chain amino acids derived target aroma compounds.

4) I wonder whether the amount of increase in aroma compound production is biotechnologically relevant. Even if the obtained increase is relatively modest, it would be important to reflect upon this point. Btw, can the AU units be interpreted as relative concentrations? If so, it would be useful to show them as relative values to parental strain. Also, are these values normalized to biomass?

In this study, our aim was to demonstrate the ability to guide the cellular evolution using metabolic models. We did not perform any sensory analysis in the study. However, once the chemical differences are observed, the winemaking protocols can be adapted to produce wines with enhanced levels of those compounds and improve the sensory impact of the evolved strains.
Absolute quantification is not possible without standards for all compounds; nevertheless, the relative comparison using AU units allowed assessing the changes from the parental strain.

5) It is unclear from reading the text how much fitness gain occurred in the evolution environment and how much fitness cost it incurred in the application environment. These would be important to show. Also, would a decreased growth rate or yield in the application environment not cancel any gain in the production of the volatile compounds? This should be clarified.

We thank the reviewer for raising this point. We had already supplemented (Table S5) the figures of major fermentation performance (i.e., ethanol, acetate, glycerol production, sugar consumption) and could, based on those, confirm that the fermentation performance was conserved including growth in the application environment (i.e., natural wine must). The growth ability of the evolved clones in wine must was ensured also by isolating the clones from synthetic wine must plates. We have now also included growth curves of the evolved isolates and the parental strain to visualize the fitness (i.e., growth as proxy) improvement (Figure 2E, please, see the revised figure with the legend below).

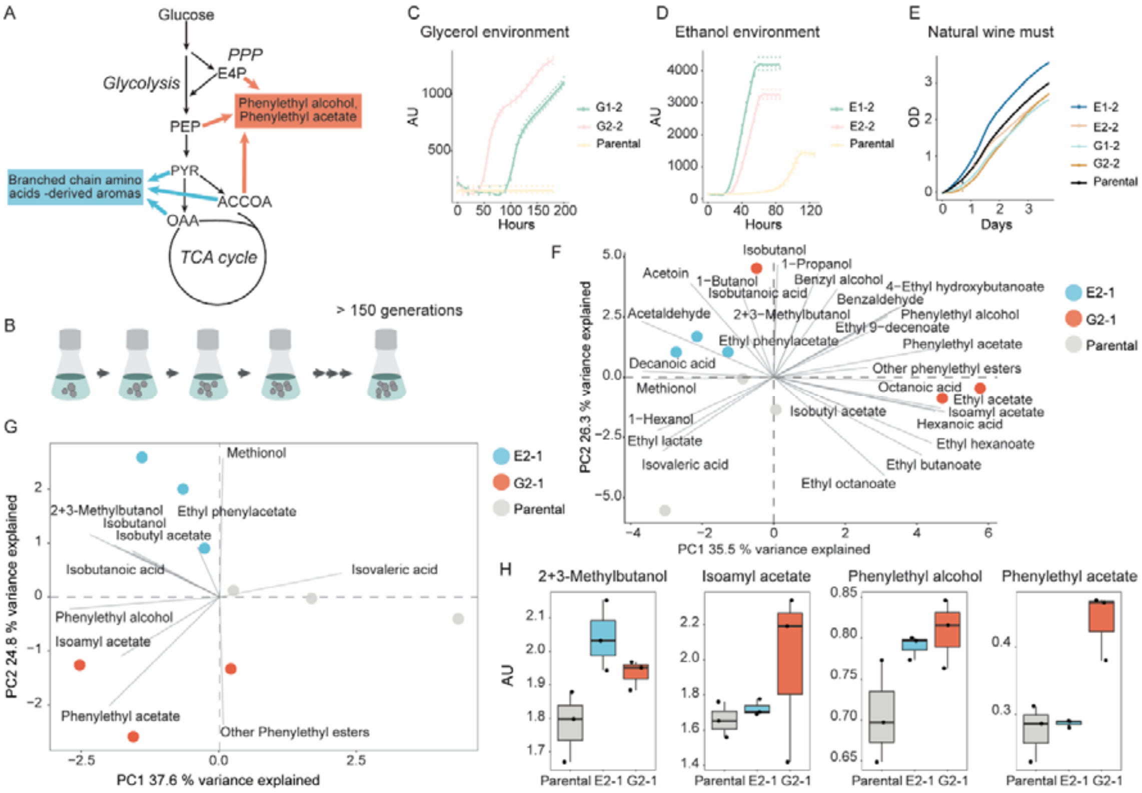

Figure 2. Aroma production changes detected in evolved yeast strains. A) Origin of aroma compounds in the yeast central metabolism: branched-chain amino acid derived compounds (esp. 2-methyl-1-butanol, 3-methyl-1-butanol, isoamyl acetate and 2-methylbutylacetate), and aromatic amino acid derived compounds (esp. phenylethyl alcohol and phenylethyl acetate). Acetate esters of higher alcohols share an acetyl-CoA (ACCOA) precursor. B) Parental wine strain of *S. cerevisiae* was adaptively evolved in both ethanol environment and glycerol environment for over 150 generations. C) Evolved single colony isolates had improved growth in glycerol environment compared to parental. The growth of isolates G1-2 and G2-2 and the parental characterized in three biological replicates as backscattered light (AU – arbitrary units). D) Evolved single colony isolates had improved growth in ethanol environment compared to parental. The growth of isolates E1-2 and E2-2 and the parental characterized in three biological replicates as backscattered light (AU – arbitrary units). E) Evolved single colony isolates maintained similar to parental growth ability characterized in single biological replicates as carbon loss in natural wine must fermentations. F) Principal components analysis of quantified 28 volatile aroma compounds in natural wine must fermentations, with the parental (grey) and evolved strains in three biological replicates. Evolved strain from the ethanol evolution environment (ethanol, arginine, glycine), E2-1, in light blue, and that from the glycerol evolution environment (glycerol, phenylalanine, threonine), G2-1, in orange. G) Principal components analysis of aromatic and branched amino acids-derived volatile compound profiles of natural wine must fermentations, with the parental (grey) and evolved strains (E2-1 in light blue, G2-1 in orange) in three biological replicates. H) Changes in selected aroma compound abundances in wine must fermentations. AU – arbitrary units. E2-1 (light blue) was selected in the ethanol environment, and G2-1 (orange) was selected in the glycerol environment. 2+3-methylbutanol (a combined pool of 2-methyl-1-butanol and 3-methyl-1-butanol) and isoamyl acetate (acetate ester of 3-methyl-1-butanol) were the desired target aromas of the ethanol environment, deriving from branched-chain amino acids. Phenylethyl alcohol and its acetate ester, phenylethyl acetate, were the desired target aromas of the glycerol environment.

6) I couldn't find a systematic comparison of predicted flux-rerouting to measured omics changes in the evolved lines. The data shown on Fig 3d-e are convincing, but a statistical analysis would be needed to support the claim that abundance changes were centered on the aroma synthesis pathways. Transcript and proteome changes that occur in multiple parallel evolved lines would be of special relevance as they likely reflect changes that are essential for the phenotype.

We performed hypergeometric tests to assess the significance of the overlaps between the protein abundance changes and the model predictions. We have revised the manuscript to support the statements on relevance of the model predictions (please, see the revised paragraphs below). Furthermore, complete RNAseq and proteomics data is included in the revised manuscript.

We have included upset plots to visualize the overlaps between differentially abundant proteins in two evolved clones from each evolution environment (compared to the parental strain grown in their respective evolution environment) (Figure 3C-D, please, see it copied below with the figure legend).

Revised text:
The fitness improvement in the glycerol environment was associated with a differential abundance of 48 and 78 metabolic enzymes in G2-1 and G2-2, respectively. In total 139 and 224 proteins were found in differential abundance compared to the parental strain (limma; n=3, P value > 0.01, -1 > log2fc >1) in G1-2 and G2-2, respectively. 66 of the proteins were shared (Figure 3C-D) marking the shared solutions in fitness improvement. Many protein down-regulations were shared between G2-1 and G2-2 (Figure 3C). The metabolic enzymes with increased abundance were enriched in respiratory pathways in accord with the strong selection pressure predicted by EvolveX (supplementary information, Table S7). A significant overlap was found between the enzymes predicted to be positively selected and the proteins present in higher abundance in the clones evolved in the glycerol environment (hypergeometric test, G2-1 P value 0.000022, G2-2 P value 0.0024). The proteins present in higher abundance in G2-2 overlapped significantly also with the tacking trait (P value 0.021). In addition, glycolytic enzymes (Cdc19, Pdc6, and Tdh1) became less abundant in G2-1, suggesting increased respiratory activity relative to glycolysis.
The fitness improvement in the ethanol environment was also associated with few, focused, enzyme abundance changes (12 in E2-1 and 31 in E2-2; limma, n=3, P value > 0.01, -1 > log2fc >1, supplementary information, Table S7). In total 19 and 68 proteins were found in differential abundance compared to the parental strain in E1-2 and E2-2, respectively. Only eight of these were shared between E2-1 and E2-2 (Figure 3C-D), underscoring the multiple evolutionary solutions to fitness improvement. The metabolic enzymes present in higher abundance in E2-2 significantly overlapped with the enzymes predicted to be positively selected in the ethanol environment (hypergeometric test, P value 0.050). Consistent with arginine as the nitrogen source in this evolution environment, the changes included decreased abundance of arginine biosynthetic pathway enzymes (Arg1 and Arg8 in E2-1, and Arg5,7 in E2-2). Strain E2-2 further had decreased abundance of proline oxidase, Put1,

involved in the utilization of one of the four nitrogen atoms in arginine. Several transporters had higher abundance in E2-2: arginine permease (Can1), monocarboxylate transporter (Jen1), methionine permease (Mup1), and hexose transporter (Hxt6). The endocytosis of all these transporters is mediated by Rsp5-Ldb19 (Becuwe & Leon, 2014; Guiney *et al*, 2016; Nikko & Pelham, 2009), which was mutated in the E2-2. Overall, in both evolution environments, the protein abundance changes were limited to the key growth-linked pathways predicted by EvolveX – respiration in the glycerol environment, and arginine metabolism in the ethanol environment.

In the application environment (wine must), the improved aroma generation was accompanied by changes in expression of around 50-200 genes (supplementary information, Table S9). Genes connected to the tacking traits and flux basis were affected, including chorismate synthesis, aromatic amino transferase, and the Ehrlich pathway (Table S2, Table S6). In G2-2, a significant overlap was detected between the corresponding flux basis of the desired trait and the genes found up-regulated (hypergeometric test, P value 0.0052). At protein level, abundance changes (limma; P value > 0.01, -1 > log2fc > 1) were observed in 9 to 32 proteins in the evolved isolates (Figure 3E). A few changes in metabolic enzymes centered on the supply of precursors to the target aroma compounds were observed (2 to 10 enzymes, Figure 3F-G). Significant overlap was detected in proteins found in higher abundance in the evolved clones and the tacking traits of the both aroma profiled evolved clones (G2-1 P value 0.011, G2-2 P value 0.017, E2-1 0.046). In E2-1 also the flux basis excluding the tacking trait overlapped significantly with the proteins in higher abundance than in the parental strain (P value 0.0064). All evolved strains exhibited increased levels of transketolase (Tkl1) consistent with increased precursor supply to aroma biosynthesis as per model prediction. The clones from the glycerol environment showed decreased levels of His1, which competes with Tkl1 for the precursor ribose 5-phosphate (Figure 3F). Another competing pathway, Orotidine-5'-phosphate decarboxylase (Ura3), involved in purine nucleotide synthesis, was also less abundant. In the ethanol environment, increased Tkl1 abundance was accompanied by those of dihydroxyacid dehydratase (Ilv3) and isopropylmalate isomerase (Leu1) (Figure 3G). Both Ilv3 and Leu1 are involved in branched-chain amino acid biosynthesis and higher activities were predicted by the model. Leu2, which follows Leu1 in the leucine biosynthesis pathway, had decreased abundance on one of the clones in accord with the model predictions (Figure 3G, supplementary information, Table S7). Overall, the protein abundance changes in evolved cells were centered on the aroma synthesis pathways consistent with the model predictions.

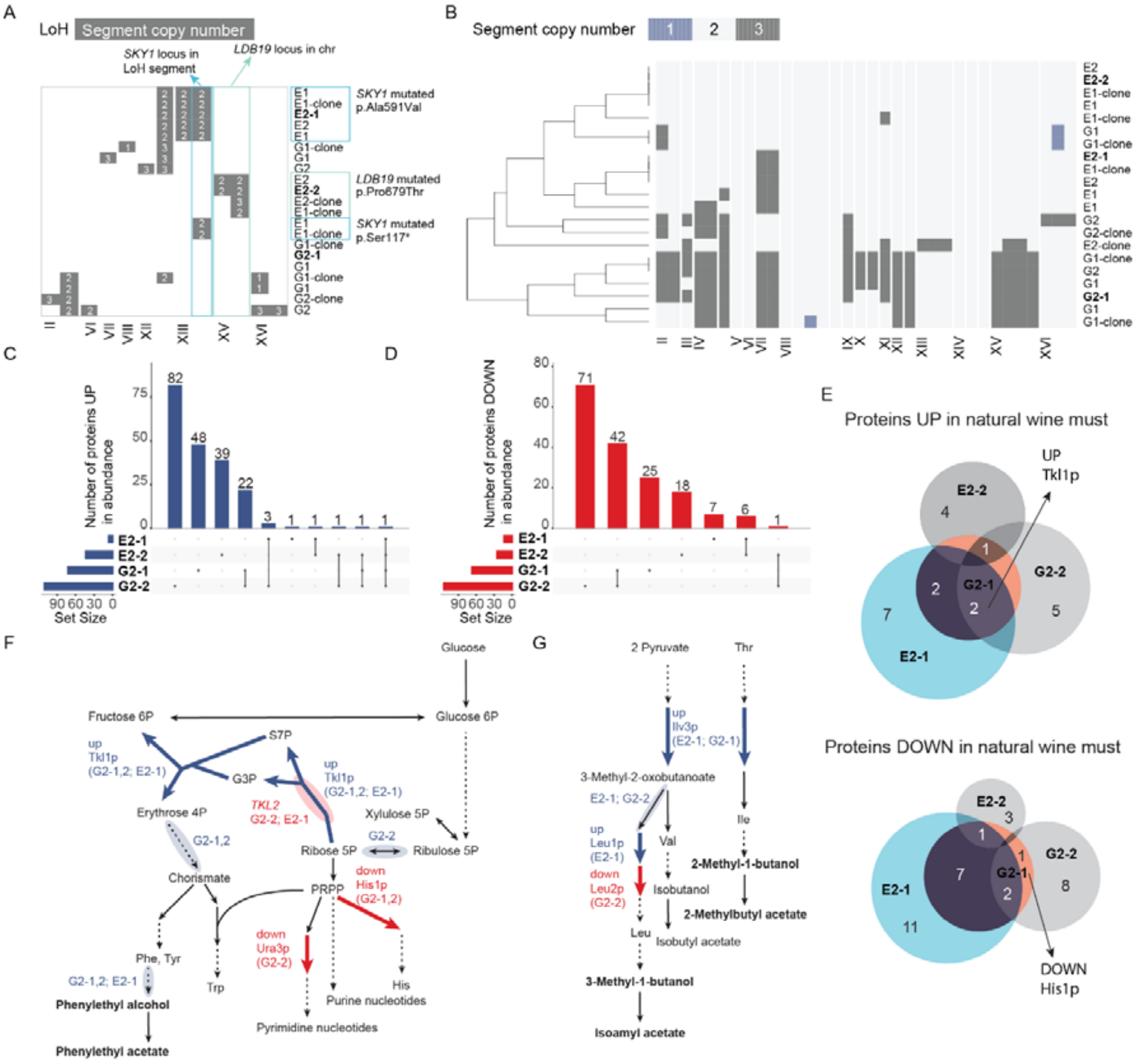

Figure 3. Molecular changes detected in evolved yeast strains. A) Loss-of-heterozygosity (LoH) coincided with single nucleotide variants (SNVs, marked on the top and right side of the panel) in evolved populations and clones from the ethanol environment, suggesting the necessity of the SNVs being homozygous for the evolved phenotype. The evolved clones ("-clone") and populations are named according to their selection environment: 'G' – glycerol selection environment. 'E' – ethanol selection environment. The number after the letter stands for the evolution status: 1 – the time of first isolation of clones, 2 – the time of second isolation of clones. The clones for which we determined protein and transcript alterations are indicated in bold. B) Evolved populations and clones from the glycerol environment exhibited large copy number variations. Shown are the genome segment copy numbers along the chromosomes. Vertical lines mark ends of contigs. C) Upset plot of sets of proteins higher in abundance (limma, n=3 (biological replicates), *P value* < 0.01, -1 > log2fc > 1) in the evolved isolates than in the parental strain in the respective evolution environments (G1-2, G2-2: glycerol environment; E1-2, E2-2 ethanol environment) shows partly shared solutions underlying improved fitness. D) Upset plot of sets of proteins lower in abundance (limma, n=3 (biological replicates), *P value* < 0.01, -1 > log2fc > 1) in the evolved isolates than in the parental strain in the respective evolution environments (G1-2, G2-2: glycerol environment; E1-2, E2-2 ethanol environment) shows proportionally large overlaps between the isolates evolved in the same environment. E) The evolved clones fermenting natural wine must (application environment) revealed both shared and evolution environment-specific protein abundance changes up and down in comparison to the parental strain (limma, n=3 (biological replicates), *P value* < 0.01, -1 > log2fc > 1). Clones for which we quantified the aroma production are shown in color (E2-1 in light blue, G2-1 in orange). Clones from the glycerol environment (G2-1, G2-2) featured higher abundance of Tkl1p (transketolase) and lower abundance of His1p (ATP phosphoribosyltransferase). F) Changes in protein (limma; n=3 (biological replicates), *P value* < 0.01, -1 > log2fc > 1) and transcript abundances (Wald test; n=3 (biological replicates), fdr < 0.05, -1 > log2 fc > 1) are centered on the pathways leading to the target aroma compounds phenylethyl alcohol and phenylethyl acetate. The changes consistent with the model predictions are indicated with colored arrows (protein-level) and clouds

around the arrows (transcript-level). G) Proteomic and transcriptomic changes in evolved clones, marked as in F), for pathways leading to the branched chain amino acids derived target aroma compounds.

7) EvolveX is indeed novel but the authors haven't discussed alternative approaches, such as making metabolite production growth coupled, e.g. Kamp & Klamt Nat Comm 2016, or performing adaptive laboratory evolution on strains in which specific production fluxes have been growth coupled first (https://doi.org/10.1002/bit.21694). It would be important to discuss how EvolveX compares to such methods in applicability. Besides, growth-coupling methods could potentially increase the scope of EvolveX by making the tacking traits growth coupled in a desired evolution environment.

We agree and have revised the introduction and discussion sections accordingly (please, see the revised paragraphs below).

Revised text:
Here, we ask whether first-principle models could enable predicting environments under which a desired trait could be adaptively selected. We base our strategy on genome-scale metabolic models, which allow predicting metabolic fluxes consistent both with the mass balance constraints and the fitness objectives of the cells (e.g. optimal growth) (O'Brien *et al*, 2015; Varma & Palsson, 1994). In the context of laboratory evolution, genome-scale metabolic models have well predicted fitness improvement and the associated metabolic flux changes (Guzman *et al*, 2019; Ibarra *et al*, 2002; Strucko *et al*, 2018; Szappanos *et al*, 2016). The genome-scale metabolic models can also be used for predicting metabolic gene deletions that couple a desired production trait to growth (Burgard *et al*, 2003; Patil *et al*, 2005). After such model-guided genome editing adaptive laboratory evolution has successfully been used to improve the growth-coupled production rates (Brochado & Patil, 2013; Burgard *et al.*, 2003; Jantama *et al*, 2008; Jensen *et al*, 2019; Pereira *et al*, 2021). We use these genome-scale metabolic models to predict environment-dependence of the coupling between metabolic traits, and that between metabolic traits and the cell fitness. This allowed us to generalize the design of evolution environments and Darwinian selection of target phenotypes.

Revised text:
A key question for generalization of the proposed strategy is which traits will be accessible through changing the growth environment. By applying the EvolveX algorithm to all reactions in of the yeast metabolism, we predict that 149 reactions can be targeted through environments composed of common nutrients; this coverage can be substantially expanded (to 273) by using enzyme inhibitors in the evolution environment. Instead of enzyme inhibitors, possible metabolic gene deletions/mutants can be used to expand the coverage. EvolveX thus extends the design of growth-product coupling (Brochado & Patil, 2013; Burgard *et al.*, 2003; Jantama *et al.*, 2008; Jensen *et al.*, 2019; Pereira *et al.*, 2021) from genotype-dependent trait-fitness dependences to also considering environment-dependence of the trait selection.

Minor points:
page 6 , line ~ 15 Please cite some literature supporting that these particular aroma compound productions are desired traits in wine yeasts.

We have added two references accordingly.

Revised text:
We targeted two main groups of aroma compounds: i) phenylethyl alcohol and its acetate ester, phenylethylacetate, which have a rose and honey scent and raspberry-like flavor; and ii) branched-chain amino acid-derived higher alcohols (2-methyl-1-butanol and 3-methyl-1-butanol) and their acetate esters (2-methylbutylacetate and isoamyl acetate) (Carpena *et al*, 2021; Swiegers *et al*, 2005), which have a banana and pear scent and fruity flavor. All these aroma compounds derive from amino acids' (L-phenylalanine and branched chain amino acids) carbon backbones and contain no nitrogen. The flux bases of the target aroma syntheses were defined as a minimum set of fluxes that have to increase for the particular target aroma generation to be enhanced. Similarly, flux bases could include fluxes that should be negatively selected for desired trait development.

In general, any environment that can sustain growth, no matter how slow, would be feasible to use. We chose for EvolveX demonstration such evolution environments among the top-scoring ones that we could, based on literature evidence, expect to sustain growth in a reasonable timeframe. How big proportion of the solutions could be considered feasible depends on what kind of and how many nutrients would be considered as potential components of an evolution environment.

page 22 , line ~ 23-27. Could you please specify which strains were tested and used in the experiments? Were strain-specific models used (i.e. from the Nielsen lab)?

We used the *S. cerevisiae* reference strain S288C model and not strain specific models. This is reported in the Material and Methods and is now included also in the Discussion (revised paragraph reproduced below). This also demonstrates that the method is not sensitive to differences beyond central pathways when the target compounds originate from the central pathways too.
The parental wine yeast strain was obtained from collaborators and the genome sequence data is deposited to ENA database (study PRJEB40761 with an accession number ERS5457098).

Revised text:
The use of model-designed evolution environment maintains the key advantage of adaptive laboratory evolution, viz. circumventing the need to know, except for the basic metabolic network structure, the genetic and regulatory basis of the traits of interest. Indeed, the omics analysis of the improved aroma generation traits in our case study revealed complex genotype-phenotype relationships. Improving these traits using rational strain improvement would currently be challenging (Hassing *et al*, 2019). As genome-scale metabolic models are becoming easier to reconstruct (Machado *et al*, 2018; Pitkanen *et al*, 2014; Seaver *et al*, 2021; Wang *et al*, 2018), our approach can be readily applied to any organism amenable for experimental evolution. Commonly a sufficient quality network is obtained in automatic model reconstruction though the accuracy is the most dependent on the success of protein functional annotation still challenging for less well characterized metabolic enzymes. In this study, we used the *S. cerevisiae* reference strain genome-scale metabolic model to represent our wine yeast parental strain. This demonstrates that the method is not sensitive to differences beyond central pathways when the target compounds originate from the central pathways too. While the choice of the parental wine strain was made based on growth in our selected evolution environments, the EvolveX method is applicable to any strain that can divide in the evolution environment.

Table S3 needs more explanation. What are the units in the top table? What is weight loss on the plot? Ideally, supplementary tables and figures should be shown separately and with captions containing enough details to be understandable by the general reader.

We have revised the supplementary information and provide it according to the journal guidelines.

Reviewer #3:
Whereas particular phenotypes such as (e.g.) oxidative stress tolerance can be readily evolved into an organism by using the appropriate evolution environment, it is difficult to use adaptive evolution to produce positive selection of phenotypes that trade-off with key fitness metric such as cell growth. Although artificial selection of maladaptive phenotypes is possible, this is usually done through combinatorial mutagenesis and is usually limited to single proteins due to the exponential demands of combinatorial programs. For complex maladaptive traits such as secretion of wine musk - sugar-rich compounds that the organism would rather eat than secrete! - such experiments are prohibited by the complexity and uncertainty of the underlying genotype.
Here, Patil and coworkers demonstrate how it is possible to positively select for otherwise neutral or maladaptive complex phenotypes by selecting for a 'tacking trait'. Here, the 'tacking trait' is an altered metabolic flux resulting from a modified growth media (the "evolution condition"). To eat the modified growth media more quickly, the organism (through natural mutation and selection over the course of several hundred cell passages within the laboratory) will gradually rewire its metabolic flux to process the modified nutrients more quickly. Hence, the 'tacking trait' is coupled to fitness (growth rate) and is therefore positively selected. When this evolved organism is then placed within a base growth media (the "application condition"), the altered metabolic flux is not preferable for cell growth but is preferable for the production of target compound(s) (in this case, wine musk). This process is suitably demonstrated in Figure 1. To predict the altered media required to produce the desirable 'tacking trait' within the 'application media', the authors devised an algorithm, called EvolveX, which identifies a tacking trait for the desirable phenotype and the set of nutrients that would be required to adaptively evolve that tacking trait.
The application of 'tacking trait'-based evolution was demonstrated in its entirety by producing a strain of S. cerevisiae with enhanced musk secretion. Although these results would have made a viable manuscript on their own standing, the authors went above and beyond by documenting the precise mutations that occurred and how these mutations resulted in the formation of both the 'tacking trait' and the desirable 'musk phenotype'. In my opinion, this manuscript is a beautiful synthesis of core evolutionary theory, computational and metabolic modelling, genomics, transcriptomics, and proteomics. Furthermore, the secretion of virtually any compound (unless a waste product) is maladaptive to the host organism, and is a considerable and persistent problem in industrial microbiology and metabolic engineering. The authors appear to be mobilizing their EvolveX program into a patentable and distributable platform. Therefore, the relevance of the manuscript is quite broad. Although prolonged exposure in the 'application condition' may result in adaptive evolution back to the original metabolic flux, the authors have emphasized that industrial scale-up is feasible in batch productions by maintaining a seed culture within the 'evolution environment'.

I am compelled to recommend 'Accept As Is' and offer my congratulations to the authors for this excellent manuscript!

I do have four comments. I will leave it to the authors' discretion whether they wish to incorporate these comments into the manuscript. This can be handled during the pre-print/proofing stage and should not delay publication:

1. For the Introduction, it could be hazardous to omit mentioning alternative works in the literature that also explore adaptive laboratory evolution for selecting maladaptive traits using metabolic modelling. For example, it's fairly common to apply adaptive evolution to a product-producing pathway by knocking out genes in the host to growth-couple product formation. These knockouts can be model-driven, from a metabolic model, somewhat similar to what was done in this manuscript. For example, I believe this functionality is now built directly into cobrapy, the most common flux balance analysis software.

We thank the reviewer for raising this point. We have revised the introduction and discussion sections accordingly (please, see the revised paragraphs below).

Revised text:
Here, we ask whether first-principle models could enable predicting environments under which a desired trait could be adaptively selected. We base our strategy on genome-scale metabolic models, which allow predicting metabolic fluxes consistent both with the mass balance constraints and the fitness objectives of the cells (e.g. optimal growth) (O'Brien *et al*, 2015; Varma & Palsson, 1994). In the context of laboratory evolution, genome-scale metabolic models have well predicted fitness improvement and the associated metabolic flux changes (Guzman *et al*, 2019; Ibarra *et al*, 2002; Strucko *et al*, 2018; Szappanos *et al*, 2016). The genome-scale metabolic models can also be used for predicting metabolic gene deletions that couple a desired production trait to growth (Burgard *et al*, 2003; Patil *et al*, 2005). After such model-guided genome editing adaptive laboratory evolution has successfully been used to improve the growth-coupled production rates (Brochado & Patil, 2013; Burgard *et al.*, 2003; Jantama *et al*, 2008; Jensen *et al*, 2019; Pereira *et al*, 2021). We use these genome-scale metabolic models to predict environment-dependence of the coupling between metabolic traits, and that between metabolic traits and the cell fitness. This allowed us to generalize the design of evolution environments and Darwinian selection of target phenotypes.

Revised text:
A key question for generalization of the proposed strategy is which traits will be accessible through changing the growth environment. By applying the EvolveX algorithm to all reactions in of the yeast metabolism, we predict that 149 reactions can be targeted through environments composed of common nutrients; this coverage can be substantially expanded (to 273) by using enzyme inhibitors in the evolution environment. Instead of enzyme inhibitors, possible metabolic gene deletions/mutants can be used to expand the coverage. EvolveX thus extends the design of growth-product coupling (Brochado & Patil, 2013; Burgard *et al.*, 2003; Jantama *et al.*, 2008; Jensen *et al.*, 2019; Pereira *et al.*, 2021) from genotype-dependent trait-fitness dependences to also considering environment-dependence of the trait selection.

2. I'm curious how the wine produced by your evolved yeast would actually taste. Yes, aroma molecules are enhanced, but it's also possible that the wine produced by these yeast either tastes or smells awful due to unanticipated negative consequences associated with changing the normal metabolic profile of yeast. Or, perhaps this will be the most delicious wine the world has ever seen! Are you following-up this work with a live application in a winery with qualitative taste tests?

In this study, our aim was to demonstrate the ability to guide the cellular evolution using metabolic models. We did not perform any sensory analysis in the study. However, once the chemical differences are observed, the winemaking protocols can be adapted to produce wines with enhanced levels of those compounds and improve the sensory impact of the evolved strains. We are following this up in a broader context of wine yeast evolution: https://www.cobiotech.eu/funded-projects/1st-call/coolwine

3. For a future manuscript, consider incorporating a value function into EvolveX such that the goal is to minimize changes to metabolites other than the metabolites of interest. This could be useful if unintended negative consequences to yeast taste/smell are indeed observed due to off-target changes within the broader metabolome (per comment #3).

We thank the reviewer for this excellent suggestion. The method indeed allows this flexibility. We have added this into the discussion (please, see the revised paragraph below).

Revised text:
The increased phenylethyl alcohol and phenylethyl acetate generation we observed occurred in an evolution environment containing the direct aroma precursor phenylalanine. In contrast, no target aroma precursor was

included in the evolution environment for the branched-chain amino acid-derived aroma compounds. This demonstrates the utility of metabolic modelling in identifying non-intuitive evolution environments. While we designed the evolution environments in this study using carbon and nitrogen sources, enzyme inhibitors and substrate analogs can also be used to expand the search space for the evolution environments. Furthermore, while the flux bases in this study included only fluxes that should be positively selected, fluxes that should be negatively selected for developing a desired trait could also be included in the design of evolution environments. The evolution environments were here also not optimized for specificity to changes in the target compounds. While the variance in aromatic and branched-chain amino acids derived aromas (including the target compounds) reflected the evolution environment of the strains, the variance in other volatile compounds can be thought to exemplify both the metabolic couplings and redundant solutions of adaptive evolution. Optimizing the evolution environment choice for the specificity of the desired trait development could be achieved by extending the EvolveX scoring scheme to flux bases that include also fluxes that should not be change in either direction.

4. Typo; Page 6 line 25 should read "An evolutionary environment"

Thank you, corrected.

1st Revision - Editorial Decision 19th Aug 2022

Manuscript Number: MSB-2022-10980R
Title: Predictive evolution of metabolic phenotypes using model-designed environments

Thank you for sending us your revised manuscript. We have now heard back from the two reviewers who were asked to evaluate your revised study. As you will see below, the reviewers think that the study has improved as a result of the performed revisions and they are supportive of publication. Reviewer #2 raises a few remaining concerns, which we would ask you to address in a minor revision.

We would also ask you to address some remaining editorial issues listed below.

Reviewer #1:

The authors have substantially improved the manuscript and addressed the main concerns I had, especially about specificity and transparency. I believe the idea is very interesting and of general interest, and now much more clearly explained. The current presentation of the results is convincing enough as a proof of concept that the idea can work. So I am satisfied.

Reviewer #2:

The authors made substantial changes to address the criticisms and, as a result, the manuscript has been greatly improved. However, I still have a couple of comments that would help to clarify certain points for the readers.

1) It might be useful to briefly mention in the Results that the diploid wine yeast chosen for evolutionary experiment was selected specifically for its ability to grow in both evolution environments.

2) Fig 2C-D appear to show only two parallel evolved populations for each selection regime. Is there a particular reason not showing the 3rd replicate lines? Btw, I found it confusing that the growth phenotypes of E2-1 and G2-1 are not shown here despite these genotypes being analyzed in-depth in the next panels.

3) How were E2-1 and G2-1 the single evolved lines selected for metabolomic analysis?

4) The authors interpret the aroma profile PCA plot on Fig 2G as follows:
'The first principal component (PC1, 37.6 % of total variance) distinguished parental from the evolved strains. In accordance with the model, this separation is driven by the overlap of the two tacking traits (transketolase and ribulose 5-phosphate 3-epimerase fluxes; supplementary information, Table S2).'
Can the authors explain how these two shared tacking traits explain the specific aroma profiles shared by the two evolved lines (e.g. enhanced phenylethyl alcohol production)? The text now implies that the model offers a mechanistic explanation for this. Or do they simply wish to say that some overlap in arome profiles are expected because there were overlap in tacking traits?

5) The authors interpret the first panel of Fig 2H as follows:
"Similarly, the combined pool of branched-chain amino acid-derived aroma compounds 2-methyl-1-butanol and 3-methyl-1-butanol was increased only for the isolate selected in the ethanol environment."
While no p-values are provided, my reading of this plot is that the G2-1 line selected in glycerol environment also increased methyl-butanols over the parental strain.

6) It would be desirable to show p-values on Fig 2H plots, especially given the low replicate numbers and high variation for some compounds. Btw, due to the low number of data points (3), instead of boxplots, the authors may consider simply showing the data points and their medians.

Thank you for sending us your revised manuscript. We have now heard back from the two reviewers who were asked to evaluate your revised study. As you will see below, the reviewers think that the study has improved as a result of the performed revisions and they are supportive of publication. Reviewer #2 raises a few remaining concerns, which we would ask you to address in a minor revision.

We would also ask you to address some remaining editorial issues listed below.

- The 11 S Tables need to be updated to EV Tables. The labelling should be Table EV1, Table EV2 etc. also in the files themselves. Please make sure that all callouts are updated in the manuscript text. The descriptions of the EV Tables should be removed from the manuscript text. Please make sure that the description of each EV Table is included in the respective .xls file (in a separate tab).

→ Tables names have been changed accordingly in the supplementary files and in the corresponding citations in the manuscript text.

Reviewer #2:

The authors made substantial changes to address the criticisms and, as a result, the manuscript has been greatly improved. However, I still have a couple of comments that would help to clarify certain points for the readers.

1) It might be useful to briefly mention in the Results that the diploid wine yeast chosen for evolutionary experiment was selected specifically for its ability to grow in both evolution environments.

→ The manuscript text is revised as suggested. Please, see the revised sentence copied below.

"In each of the two selected environments, three replicate populations of a diploid wine yeast strain (selected based on capability of growing in both environments) were independently evolved asexually for over 150 generations (Figure 2B)."

2) Fig 2C-D appear to show only two parallel evolved populations for each selection regime. Is there a particular reason not showing the 3rd replicate lines? Btw, I found it confusing that the growth phenotypes of E2-1 and G2-1 are not shown here despite these genotypes being analyzed in-depth in the next panels.

→ Figure 2C-D shows the growth performance of the isolates that were characterized using RNA-sequencing and proteomics. The growth curves were characterized in connection to the corresponding experiments. We had mistakenly swapped the numbers of the labels of two isolates G2-1 and E2-1, as G1-2 and E1-2, respectively. The swap is now corrected in Figure 2C-E and its legend. They revised figure is shown below.

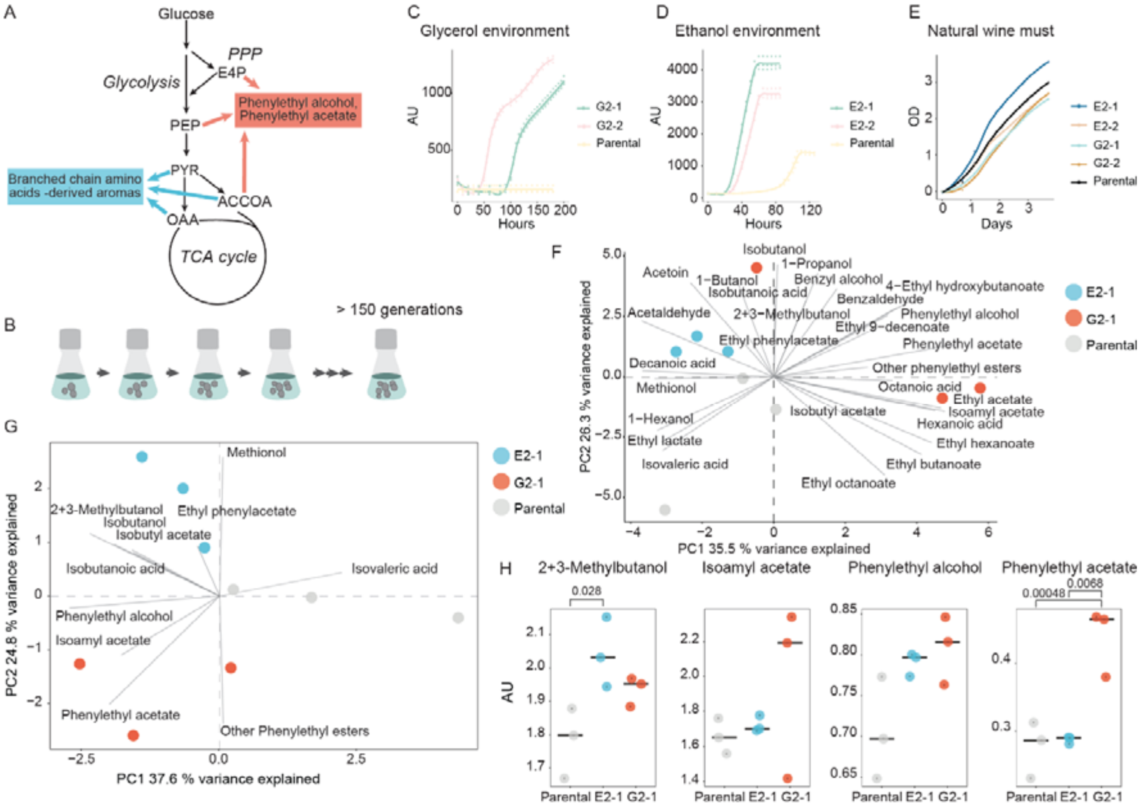

Figure 2. Aroma production changes detected in evolved yeast strains. A) Origin of aroma compounds in the yeast central metabolism: branched-chain amino acid derived compounds (esp. 2-methyl-1-butanol, 3-methyl-1-butanol, isoamyl acetate and 2-methylbutylacetate), and aromatic amino acid derived compounds (esp. phenylethyl alcohol and phenylethyl acetate). Acetate esters of higher alcohols share an acetyl-CoA (ACCOA) precursor. B) Parental wine strain of *S. cerevisiae* was adaptively evolved in both ethanol environment and glycerol environment for over 150 generations. C) Evolved single colony isolates had improved growth in glycerol environment compared to parental. The growth of isolates G2-1 and G2-2 and the parental characterized in three biological replicates as backscattered light (AU – arbitrary units). D) Evolved single colony isolates had improved growth in ethanol environment compared to parental. The growth of isolates E2-1 and E2-2 and the parental characterized in three biological replicates as backscattered light (AU – arbitrary units). E) Evolved single colony isolates maintained similar to parental growth ability characterized in single biological replicates as carbon loss in natural wine must fermentations. F) Principal components analysis of quantified 28 volatile aroma compounds in natural wine must fermentations, with the parental (grey) and evolved strains in three biological replicates. Evolved strain from the ethanol evolution environment (ethanol, arginine, glycine), E2-1, in light blue, and that from the glycerol evolution environment (glycerol, phenylalanine, threonine), G2-1, in orange. G) Principal components analysis of aromatic and branched amino acids-derived volatile compound profiles of natural wine must fermentations, with the parental (grey) and evolved strains (E2-1 in light blue, G2-1 in orange) in three biological replicates. H) Changes in selected aroma compound abundances in wine must fermentations. AU – arbitrary units. E2-1 (light blue) was selected in the ethanol environment, and G2-1 (orange) was selected in the glycerol environment. 2+3-methylbutanol (a combined pool of 2-methyl-1-butanol and 3-methyl-1-butanol) and isoamyl acetate (acetate ester of 3-methyl-1-butanol) were the desired target aromas of the ethanol environment, deriving from branched-chain amino acids. Phenylethyl alcohol and its acetate ester, phenylethyl acetate, were the desired target aromas

of the glycerol environment. Medians over three biological replicates are shown with black lines. Significant differences in means (Tukey's test; n=3; *P value* < 0.05) are indicated with *P values*.

3) How were E2-1 and G2-1 the single evolved lines selected for metabolomic analysis?

→ One strain was arbitrarily selected from each evolution environment for the natural wine must fermentation and aroma profiling. Then, these strains and one additional from each evolution environment were further characterized using RNA sequencing and proteomics. In future work, it would be interesting to characterize many more isolates evolved in different designed evolution environments and assess how the desired traits correlate with growth parameters in the evolution environment.

Please, see the revised manuscript text copied below.

"For discarding non-genetic adaptation and for ensuring wine fermentation performance, single colonies were picked from the evolved lineages following growth on WMM + 2% agar plates for 48 h. Nine single colonies were isolated from each lineage and cultured overnight in liquid cultures on WMM. From these overnight cultures, stocks were prepared to 30% w/v Glycerol and stored at -80 oC. The overnight cultures on WMM were also used to inoculate corresponding evolution environment as in adaptive laboratory evolution. Cell growth was monitored with turbidity (OD600) measurements. One arbitrarily selected strain from each evolution environment was characterized in wine fermentation mimicking conditions."

4) The authors interpret the aroma profile PCA plot on Fig 2G as follows:

'The first principal component (PC1, 37.6 % of total variance) distinguished parental from the evolved strains. In accordance with the model, this separation is driven by the overlap of the two tacking traits (transketolase and ribulose 5-phosphate 3-epimerase fluxes; supplementary information, Table S2).'

Can the authors explain how these two shared tacking traits explain the specific aroma profiles shared by the two evolved lines (e.g. enhanced phenylethyl alcohol production)? The text now implies that the model offers a mechanistic explanation for this. Or do they simply wish to say that some overlap in arome profiles are expected because there were overlap in tacking traits?

→ Common changes in the aroma profiles with respect to the parental strain were expected because the model predicted tacking traits, and the fluxes under selection in the two evolution environments, were partially overlapping. We have revised the manuscript text to clarify this as below.

"The first principal component (PC1, 37.6 % of total variance) distinguished parental from the evolved strains. In accordance with the model predicted overlap of the tacking traits (transketolase and ribulose 5-phosphate 3-epimerase fluxes; supplementary information, Table EV2) and fluxes under selection (supplementary information, Table EV3), common separation from the parental aroma profile was expected."

5) The authors interpret the first panel of Fig 2H as follows:

"Similarly, the combined pool of branched-chain amino acid-derived aroma compounds 2-methyl-1-butanol and 3-methyl-1-butanol was increased only for the isolate selected in the ethanol environment."

While no p-values are provided, my reading of this plot is that the G2-1 line selected in glycerol environment also increased methyl-butanols over the parental strain.

→ Please see response to the next point.

6) It would be desirable to show p-values on Fig 2H plots, especially given the low replicate numbers and high variation for some compounds. Btw, due to the low number of data points (3), instead of boxplots, the authors may consider simply showing the data points and their medians.

→ We performed Tukey's tests for assessing the statistical significance of the differences between the aroma production. We have revised the Figure 2H to indicate the P values where below threshold. The revised figure and the legend are copied below.

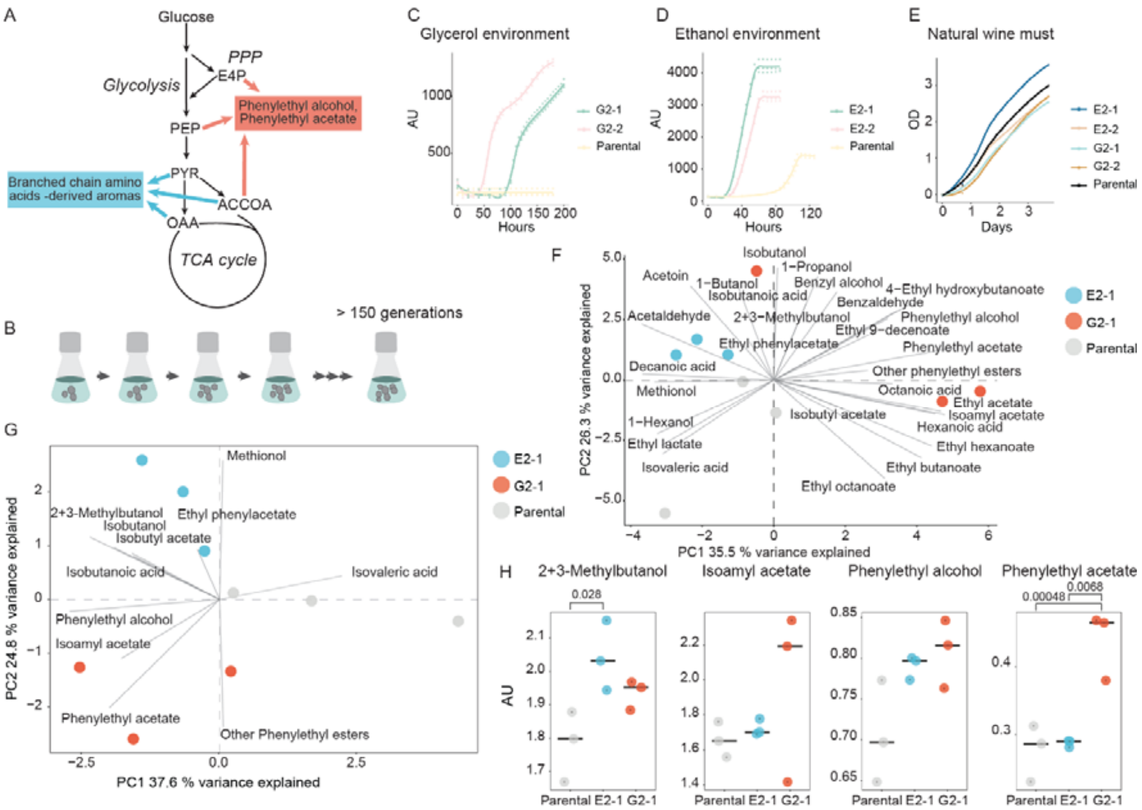

Figure 2. Aroma production changes detected in evolved yeast strains. A) Origin of aroma compounds in the yeast central metabolism: branched-chain amino acid derived compounds (esp. 2-methyl-1-butanol, 3-methyl-1-butanol, isoamyl acetate and 2-methylbutylacetate), and aromatic amino acid derived compounds (esp. phenylethyl alcohol and phenylethyl acetate). Acetate esters of higher alcohols share an acetyl-CoA (ACCOA) precursor. B) Parental wine strain of *S. cerevisiae* was adaptively evolved in both ethanol environment and glycerol environment for over 150 generations. C) Evolved single colony isolates had improved growth in glycerol environment compared to parental. The growth of isolates G2-1 and G2-2 and the parental characterized in three biological replicates as backscattered light (AU – arbitrary units). D) Evolved single colony isolates had improved growth in ethanol

environment compared to parental. The growth of isolates E2-1 and E2-2 and the parental characterized in three biological replicates as backscattered light (AU – arbitrary units). E) Evolved single colony isolates maintained similar to parental growth ability characterized in single biological replicates as carbon loss in natural wine must fermentations. F) Principal components analysis of quantified 28 volatile aroma compounds in natural wine must fermentations, with the parental (grey) and evolved strains in three biological replicates. Evolved strain from the ethanol evolution environment (ethanol, arginine, glycine), E2-1, in light blue, and that from the glycerol evolution environment (glycerol, phenylalanine, threonine), G2-1, in orange. G) Principal components analysis of aromatic and branched amino acids-derived volatile compound profiles of natural wine must fermentations, with the parental (grey) and evolved strains (E2-1 in light blue, G2-1 in orange) in three biological replicates. H) Changes in selected aroma compound abundances in wine must fermentations. AU – arbitrary units. E2-1 (light blue) was selected in the ethanol environment, and G2-1 (orange) was selected in the glycerol environment. 2+3-methylbutanol (a combined pool of 2-methyl-1-butanol and 3-methyl-1-butanol) and isoamyl acetate (acetate ester of 3-methyl-1-butanol) were the desired target aromas of the ethanol environment, deriving from branched-chain amino acids. Phenylethyl alcohol and its acetate ester, phenylethyl acetate, were the desired target aromas of the glycerol environment. Medians over three biological replicates are shown with black lines. Significant differences in means (Tukey's test; n=3; *P value* < 0.05) are indicated with *P values*.

2nd Revision - Editorial Decision 26th Aug 2022

RE: MSB-2022-10980RR, Predictive evolution of metabolic phenotypes using model-designed environments

Thank you again for sending us your revised manuscript. We are now satisfied with the modifications made and I am pleased to inform you that your paper has been accepted for publication.

EMBO Press Author Checklist

Corresponding Author Name: Kiran R. Patil
Journal Submitted to: Molecular Systems Biology
Manuscript Number: MSB-2022-10980

USEFUL LINKS FOR COMPLETING THIS FORM
The EMBO Journal - Author Guidelines
EMBO Reports - Author Guidelines
Molecular Systems Biology - Author Guidelines
EMBO Molecular Medicine - Author Guidelines

Reporting Checklist for Life Science Articles (updated January
This checklist is adapted from Materials Design Analysis Reporting (MDAR) Checklist for Authors. MDAR establishes a minimum set of requirements in transparent reporting in the life sciences (see Statement of Task: 10.31222/osf.io/9sm4x). Please follow the journal's guidelines in preparing your manuscript.
Please note that a copy of this checklist will be published alongside your article.

Abridged guidelines for figures
1. Data
The data shown in figures should satisfy the following conditions:
- the data were obtained and processed according to the field's best practice and are presented to reflect the results of the experiments in an accurate and unbiased manner.
- ideally, figure panels should include only measurements that are directly comparable to each other and obtained with the same assay.
- plots include clearly labeled error bars for independent experiments and sample sizes. Unless justified, error bars should not be shown for technical
- if n<5, the individual data points from each experiment should be plotted. Any statistical test employed should be justified.
- Source Data should be included to report the data underlying figures according to the guidelines set out in the authorship guidelines on Data

2. Captions
Each figure caption should contain the following information, for each panel where they are relevant:
- a specification of the experimental system investigated (eg cell line, species name).
- the assay(s) and method(s) used to carry out the reported observations and measurements.
- an explicit mention of the biological and chemical entity(ies) that are being measured.
- an explicit mention of the biological and chemical entity(ies) that are altered/varied/perturbed in a controlled manner.
- the exact sample size (n) for each experimental group/condition, given as a number, not a range;
- a description of the sample collection allowing the reader to understand whether the samples represent technical or biological replicates (including how many animals, litters, cultures, etc.).
- a statement of how many times the experiment shown was independently replicated in the laboratory.
- definitions of statistical methods and measures:
  - common tests, such as t-test (please specify whether paired vs. unpaired), simple χ2 tests, Wilcoxon and Mann-Whitney tests, can be unambiguously identified by name only, but more complex techniques should be described in the methods section;
  - are tests one-sided or two-sided?
  - are there adjustments for multiple comparisons?
  - exact statistical test results, e.g., P values = x but not P values < x;
  - definition of 'center values' as median or average;
  - definition of error bars as s.d. or s.e.m.

Please complete ALL of the questions below.
Select "Not Applicable" only when the requested information is not relevant for your study.

Materials

**Newly Created Materials**	**Information included in the manuscript?**	**In which section is the information available?** (Reagents and Tools Table, Materials and Methods, Figures, Data Availability Section)
New materials and reagents need to be available; do any restrictions apply?	Not Applicable	

**Antibodies**	**Information included in the manuscript?**	**In which section is the information available?** (Reagents and Tools Table, Materials and Methods, Figures, Data Availability Section)
For **antibodies** provide the following information:<br>- Commercial antibodies: RRID (if possible) or supplier name, catalogue number and or/clone number<br>- Non-commercial: RRID or citation	Not Applicable	

**DNA and RNA sequences**	**Information included in the manuscript?**	**In which section is the information available?** (Reagents and Tools Table, Materials and Methods, Figures, Data Availability Section)
**Short novel DNA or RNA including primers, probes**: provide the sequences.	Not Applicable	

**Cell materials**	**Information included in the manuscript?**	**In which section is the information available?** (Reagents and Tools Table, Materials and Methods, Figures, Data Availability Section)
**Cell lines:** Provide species information, strain. Provide accession number in repository **OR** supplier name, catalog number, clone number, and/**OR** RRID.	Not Applicable	
**Primary cultures:** Provide species, strain, sex of origin, genetic modification status.	Not Applicable	
Report if the cell lines were recently **authenticated** (e.g., by STR profiling) and tested for mycoplasma contamination.	Not Applicable	

**Experimental animals**	**Information included in the manuscript?**	**In which section is the information available?** (Reagents and Tools Table, Materials and Methods, Figures, Data Availability Section)
**Laboratory animals or Model organisms:** Provide species, strain, sex, age, genetic modification status. Provide accession number in repository **OR** supplier name, catalog number, clone number, **OR** RRID.	Not Applicable	
**Animal observed in or captured from the field:** Provide species, sex, and age where possible**.**	Not Applicable	
Please detail **housing and husbandry conditions**.	Not Applicable	

**Plants and microbes**	**Information included in the manuscript?**	**In which section is the information available?** (Reagents and Tools Table, Materials and Methods, Figures, Data Availability Section)
**Plants:** provide species and strain, ecotype and cultivar where relevant, unique accession number if available, and source (including location for collected wild specimens).	Not Applicable	
**Microbes:** provide species and strain, unique accession number if available, and source.	Yes	Material and Methods section describes the organism studied. Data Availability Section provides information where to obtain the genome sequences of the strains used in the study: Whole genome sequencing

**Human research participants**	**Information included in the manuscript?**	**In which section is the information available?** (Reagents and Tools Table, Materials and Methods, Figures, Data Availability Section)
If collected and within the bounds of privacy constraints report on age, sex and gender or ethnicity for all study participants.	Not Applicable	

**Core facilities**	**Information included in the manuscript?**	**In which section is the information available?** (Reagents and Tools Table, Materials and Methods, Figures, Data Availability Section)
If your work benefited from core facilities, was their service mentioned in the acknowledgments section?	Yes	Acknowledgements section: We acknowledge the support of the following core facilities at the European Molecular Biology Laboratory (Heidelberg, Germany): Genomics (V. Benes and R. Hercog), and Proteomics (M. Rettel and F. Stein).

Design

Study protocol	Information included in the manuscript?	In which section is the information available? (Reagents and Tools Table, Materials and Methods, Figures, Data Availability Section)
If study protocol has been **pre-registered, provide DOI in the manuscript**. For clinical trials, provide the trial registration number **OR** cite DOI.	Not Applicable	
Report the **clinical trial registration number** (at ClinicalTrials.gov or equivalent), where applicable.	Not Applicable	

Laboratory protocol	Information included in the manuscript?	In which section is the information available? (Reagents and Tools Table, Materials and Methods, Figures, Data Availability Section)
Provide DOI OR other citation details if **external detailed step-by-step protocols** are available.	Not Applicable	

Experimental study design and statistics	Information included in the manuscript?	In which section is the information available? (Reagents and Tools Table, Materials and Methods, Figures, Data Availability Section)
Include a statement about **sample size** estimate even if no statistical methods were used.	Yes	Material and Methods section: All experiments were performed using biological replicates. No prior assumptions were made regarding effect size to choose the sample size.
Were any steps taken to minimize the effects of subjective bias when allocating animals/samples to treatment (e.g. **randomization procedure**)? If yes, have they been described?	Yes	Materials and Methods section: Experimental study design did not include randomization.
Include a statement about **blinding** even if no blinding was done.	Yes	Materials and Methods section: Experimental study design did not include blinding.
Describe **inclusion/exclusion criteria** if samples or animals were excluded from the analysis. Were the criteria pre-established?<br><br>If sample or data points were omitted from analysis, report if this was due to attrition or intentional exclusion and provide justification.	Yes	Material and Methods section: Evolved clones were selected for phenotyping based on single replicate growth screening. No samples were excluded from further analysis.
For every figure, are **statistical tests** justified as appropriate? Do the data meet the assumptions of the tests (e.g., normal distribution)? Describe any methods used to assess it. Is there an estimate of variation within each group of data? Is the variance similar between the groups that are being statistically compared?	Yes	Material and Methods section, Assumption of normally distributed data was not tested as number of samples in each group are small. Variance in the data is shown in figures and can be estimated from the raw data. Similarity of variance between the groups being compared is case-dependent. The data variance is shown in Figures and can be estiamted in the raw data.

Sample definition and in-laboratory replication	Information included in the manuscript?	In which section is the information available? (Reagents and Tools Table, Materials and Methods, Figures, Data Availability Section)
In the figure legends: state number of times the experiment was **replicated** in laboratory.	Yes	Figure legends, Material and Methods
In the figure legends: define whether data describe **technical or biological replicates**.	Yes	Figure legends, Material and Methods

Ethics

Ethics	Information included in the manuscript?	In which section is the information available? (Reagents and Tools Table, Materials and Methods, Figures, Data Availability Section)
Studies involving **human participants**: State details of **authority granting ethics approval** (IRB or equivalent committee(s), provide reference number for approval.	Not Applicable	
Studies involving **human participants**: Include a statement confirming that **informed consent** was obtained from all subjects and that the experiments conformed to the principles set out in the WMA Declaration of Helsinki and the Department of Health and Human Services Belmont Report.	Not Applicable	
Studies involving **human participants:** For publication of **patient photos**, include a statement confirming that consent to publish was obtained.	Not Applicable	
Studies involving experimental **animals**: State details of **authority granting ethics approva**l (IRB or equivalent committee(s), provide reference number for approval. Include a statement of compliance with ethical regulations.	Not Applicable	
Studies involving **specimen and field samples:** State if relevant **permits** obtained, provide details of authority approving study; if none were required, explain why.	Not Applicable	

Dual Use Research of Concern (DURC)	Information included in the manuscript?	In which section is the information available? (Reagents and Tools Table, Materials and Methods, Figures, Data Availability Section)
Could your study fall under dual use research restrictions? Please check biosecurity documents and list of **select agents and toxins** (CDC): https://www.selectagents.gov/sat/list.htm	Not Applicable	
If you used a select agent, is the security level of the lab appropriate and reported in the manuscript?	Not Applicable	
If a study is subject to dual use research of concern regulations, is the name of the **authority granting approval and reference number** for the regulatory approval provided in the manuscript?	Not Applicable	

Reporting

The MDAR framework recommends adoption of discipline-specific guidelines, established and endorsed through community initiatives. Journals have their own policy about requiring specific guidelines and recommendations to complement MDAR.

Adherence to community standards	Information included in the manuscript?	In which section is the information available? (Reagents and Tools Table, Materials and Methods, Figures, Data Availability Section)
State if relevant guidelines or checklists (e.g., **ICMJE, MIBBI, ARRIVE, PRISMA**) have been followed or provided.	Not Applicable	
For **tumor marker prognostic studies,** we recommend that you follow the **REMARK** reporting guidelines (see link list at top right). See author guidelines, under 'Reporting Guidelines'. Please confirm you have followed these guidelines.	Not Applicable	
For **phase II and III randomized controlled trials**, please refer to the **CONSORT** flow diagram (see link list at top right) and submit the CONSORT checklist (see link list at top right) with your submission. See author guidelines, under 'Reporting Guidelines'. Please confirm you have submitted this list.	Not Applicable	

Data Availability

Data availability	Information included in the manuscript?	In which section is the information available? (Reagents and Tools Table, Materials and Methods, Figures, Data Availability Section)
Have **primary datasets** been deposited according to the journal's guidelines (see 'Data Deposition' section) and the respective accession numbers provided in the Data Availability Section?	Yes	Data Availability Section: - Whole genome sequencing data: European Nucleotide Archive (ENA) study PRJEB40761 with accession numbers ERS5457098 and ERS5290477-ERS5290502 for the parental and evolved samples and in study PRJEB41108 with accession numbers ERS5293678-
Were **human clinical and genomic datasets** deposited in a public access-controlled repository in accordance to ethical obligations to the patients and to the applicable consent agreement?	Not Applicable	
Are **computational models** that are central and integral to a study available without restrictions in a machine-readable form? Were the relevant accession numbers or links provided?	Yes	Data Availability Section: Modeling computer scripts: GitHub (https://github.com/ptjouhten/EvolveX)
If publicly available data were reused, provide the respective **data citations in the reference list**.	Not Applicable	
